# Trust in Context: A Three-Factor Experimental Study

**DOI:** 10.3390/bs16061001

**Published:** 2026-06-15

**Authors:** Jiayin Guo, Jun Liu

**Affiliations:** School of Humanities and Social Sciences, Harbin Engineering University, No. 145, Nantong Street, Nangang District, Harbin 150001, China; gjy@hrbeu.edu.cn

**Keywords:** trust, entrusted matters, interaction effect, contextual experiment

## Abstract

Existing studies on trust are mainly based on rational choice theory or the relational logic of the “differential mode of association,” while neglecting the contextuality of trust and the interaction of multiple factors. This study used a within-subjects situational experiment involving 252 participants, manipulating three variables: relationship type (kin, acquaintance, general other), entrusted matter (loans of 2000 yuan, 20,000 yuan, and 200,000 yuan), and trustee attributes (high ability and integrity vs. low ability and integrity). The Friedman test and Generalized Estimating Equations (GEE) were used to examine the effects of these three factors on trust intention and the mechanisms of their interaction. The results indicate that trust intention is influenced by relationship type, the importance of the entrusted matter, and trustee attributes, with significant interactions among the three. This indicates that trust is a contextual outcome shaped by multiple interacting factors rather than a linear result. This study provides contextualized evidence that relationship type, entrusted matter, and trustee attributes jointly shape trust intention.

## 1. Introduction

With the increasing specialization of the social division of labor and the growing complexity of social relations, trust has become an increasingly important foundation for social cooperation. Simmel (2011, p. 191) pointed out that society itself would disintegrate without trust. Luhmann et al. (2017) also suggested that everyday life could not function without trust. However, along with the acceleration of modernization, social differentiation and intensified population mobility have made individuals’ social interactions increasingly complex and changeable, and trust risks and uncertainties have increased accordingly. In daily life, phenomena such as fraud, falsification, deception, and “friendship pricing” continue to emerge. These problems not only damage interpersonal relationships but also threaten the stability of social order. Therefore, trust has become an important topic in social science research.

Overall, existing studies on trust can be broadly classified into two main perspectives: the rational choice perspective and the relational perspective. The rational choice perspective conceptualizes trust as a calculated decision based on cost–benefit considerations (Berg et al., 1995; Coleman, 1990; Hardin, 2001). For example, when lending money, people tend to evaluate the borrower’s repayment capacity, financial situation, and risk of default. If the potential losses are excessively high, people may become cautious or unwilling to trust, even when they have a close relationship with the borrower.

In contrast, the relational perspective emphasizes that social relationships (guanxi) are central to understanding trust in the Chinese sociocultural context. Based on Fei Xiaotong’s theory of the “differential mode of association (Fei, 1992, pp. 67–68),” scholars argue that trust follows a hierarchical pattern centered on kinship, namely “kin > acquaintances > strangers”. This pattern has been widely supported by empirical evidence (Bai et al., 2019; H. Liu et al., 2023). This perspective holds that in routine matters (such as pet-sitting or key-keeping), people are more likely to trust relatives and acquaintances than strangers.

In fact, trust is far more complex than theoretical assumptions. On the one hand, the rational choice perspective overemphasizes rational calculation and neglects the fact that trust is always embedded in specific relationships (such as parent–child, friends, business partners), where emotion and reason coexist. In daily life, the formation of trust does not always require a careful assessment of costs and benefits. For example, within harmonious parent–child relationships, parents and children rarely calculate whether their contributions and returns are equivalent.

On the other hand, although the relational perspective recognizes the importance of social ties, it fails to acknowledge that trust remains selective even within established relationships. For example, although A maintains friendships with both B and C, A may be more willing to trust B than C in some situations, such as lending money, while trusting C more than B in other matters, such as keeping secrets. Moreover, numerous real-world cases demonstrate that relatives and acquaintances are not always trustworthy. These cases suggest that relationships do not automatically guarantee trust. In specific situations, emotional and moral obligations may also be replaced by considerations of interests and risks.

This study adopts a situational experiment to examine trust intentions as shaped by relationship type, entrusted matter, and trustee attributes. Four research questions are addressed: (1) Do relationship type and the importance of the entrusted matter each exert significant effects on trust intention? (2) Is there an interaction between relationship type and the entrusted matter, such that trust intention within the same relationship changes as the importance of the entrusted matter varies? (3) Do trustee attributes, namely competence and integrity, influence the trustor’s trust intention? (4) Is there a three-way interaction among relationship type, the entrusted matter, and trustee attributes? Through these analyses, this study aims to provide situational evidence that relationship type, entrusted matter, and trustee attributes jointly influence trust intention, thereby deepening our understanding of trust in specific contexts and offering new perspectives for the measurement and modeling of trust.

## 2. Literature Review and Theoretical Framework

### 2.1. Literature Review

(1) Methods for Measuring Trust

At present, questionnaire surveys and experimental methods constitute the two main approaches used to measure trust in academic research. Among questionnaire-based approaches, two major measurement strategies are commonly used. One is drawing on multi-item trust scales, such as Rotter’s Interpersonal Trust Scale (ITS), Johnson-George’s Specific Interpersonal Trust Scale (SITS), and the Trust Scale developed by Rempel and Holmes. The other relies on single-item measures used in large-scale social surveys, including CSS, CGSS, and WVS (Newton & Zmerli, 2011; H. Xu et al., 2023). Typical items include questions such as “Generally speaking, would you say that most people can be trusted, or that you cannot be too careful in dealing with people?” Respondents usually answer on a scale ranging from 1 to 10 to indicate their level of trust.

In practice, both single-item and multi-item questionnaire measures of trust suffer from several design problems. First, the object of trust is unclear. For example, the widely used question “Generally speaking, do you think most people can be trusted?” contains an ambiguous concept. The phrase “most people” is vague and may be understood differently by different respondents (Nannestad, 2008; Sturgis & Smith, 2010). Bauer and Freitag (2017) asked respondents whom they had in mind when answering questions about “most people.” Many respondents reported imagining specific situations, such as someone they encountered on a train or in the street, or people they personally knew. Therefore, differences in survey results may reflect variations in respondents’ interpretations of the question rather than actual differences in their levels of trust. As a result, the comparability of the data is greatly reduced.

Second, when researchers use trust survey questionnaires, they often fail to consider the specific scenarios on which respondents rely when making trust decisions. For example, although respondents may be asked the same question about “whether most people can be trusted,” their actual trust intention may vary substantially across different situations, such as asking strangers for directions, lending money to others, or entrusting personal privacy to others.

Third, measurement inherently involves a relationship between “language” and “reality”. In conventional measurements of interpersonal trust, once researchers operationalize trust, what is actually measured is no longer trust itself but only selected attributes of trust. As a result, the findings inevitably reflect the researchers’ subjective constructions to some extent. In previous studies, we argued that trust in the Chinese context is a multidimensional phenomenon involving the trustor, the trustee, the relationship type, and the entrusted matter (e.g., pet care, lending money, or job information), rather than a simple correspondence between numbers and measured objects defined by specific rules (J. Liu & Guo, 2024). Moreover, when research subjects are transformed into abstract objects of measurement, important elements such as thoughts, beliefs, consciousness, language, social relations, and cultural backgrounds are often overlooked. Consequently, measurements detached from concrete contexts cannot fully capture or reflect the phenomenon of trust.

To better capture the contextual aspects of trust, many scholars have adopted experimental methods and developed two main paradigms based on game theory: the Prisoner’s Dilemma and the Trust Game (Tedeschi et al., 1969; Berg et al., 1995; Suneja & Das, 2024). Although these paradigms, grounded in rational choice theory, offer some explanatory power in analyzing how individuals make behavioral decisions, they still have some limitations. For example, variations in experimental rules can significantly affect research findings. In trust game experiments, there is no standardized multiplier for the amount transferred by the trustor. Some studies follow Berg’s original design and use a multiplier of three, while others adopt a multiplier of two or other values (Glaeser et al., 2000). Consequently, results across studies become difficult to compare. Moreover, although trust game experiments incorporate risk into their design, they do not fully account for the complex situational factors involved in trust in real-life situations.

(2) Conceptual Definition of Trust

Although trust has been widely studied in academia, there is still no clear consensus regarding its conceptual definition. On the one hand, many scholars treat interpersonal trust as a “self-evident” concept and thus do not provide an explicit definition. For example, some studies measure interpersonal trust by asking respondents to rate their level of trust toward various social groups, such as relatives, neighbors, colleagues, classmates, friends, online contacts, and people from the same hometown (Zhang, 2009).

On the other hand, even when scholars provide explicit definitions of trust, these definitions often focus on categorizing trust targets rather than examining the underlying meaning of trust itself. For example, some studies conceptualize interpersonal trust as “the degree of trust that a trustor places in individuals with specific social identities.” These “identity-based” categories include one’s own kin, relatives, friends, clan members, fellow villagers, village officials, people introduced by relatives or friends, frequent business partners, classmates, and “most people in society”, and are used to assess respondents’ levels of interpersonal trust (Yu, 2017)^1^. However, such a definition is clearly tautological.

Moreover, other scholars define interpersonal trust in modern society as “trust that is not restricted to specific targets, not directed toward particular individuals, and oriented toward all members of society,” referring to it as “generalized trust” in contrast to particularized trust (Qi & Zhang, 2022)^2^. Although these studies appear to offer conceptual definitions of interpersonal trust, they fail to capture its substantive core. As a result, they do not offer a sufficiently clear understanding of what trust actually is.

In addition to discussions of the mechanisms of trust formation, existing studies have also distinguished between generalized trust and particularized trust based on the scope of trust targets. Generalized trust refers to a basic tendency to trust general others, including strangers. It reflects an abstract attitude that “most people are generally trustworthy,” independent of specific relationships. In contrast, particularized trust is embedded in concrete social relationships, grounded in the nature and closeness of relational ties, and exhibits a clear differential pattern (Newton & Zmerli, 2011; Uslaner, 2002; Yamagishi et al., 1998).

However, the form of trust examined in this study differs from both generalized trust and particularized trust. On the one hand, in situations involving real stakes and risk, people often rely on relational information or reputational cues to evaluate whether a trustee is reliable. This is a dynamic process that the abstract attitudinal measures of generalized trust cannot fully capture. On the other hand, existing research on particularized trust mainly focuses on the influence of distance on trust, while paying less attention to how trust intentions may change across different situations within the same relationships. As discussed above, even toward the same relational partner, an individual’s trust intention may vary substantially across different contexts. Therefore, we use the term “general others” rather than “strangers” to reflect our focus on trust within minimally established social relationships, which differs from the abstract attitudinal orientation in generalized trust.

### 2.2. Theoretical Framework

Scholars have proposed different understandings of trust. Coleman (1990) regards trust as a rational decision based on cost and benefit calculations. Rotter (1967) defines trust as a generalized expectation regarding the reliability of others’ words, promises, and oral or written statements. In addition, some scholars argue that trust involves three elements. For example, Cook et al. (2007) suggests that we should not simply say “a trusts b”, but rather “a trusts b to do x.” Similarly, Hardin (2002, p. 9) defines trust as “a trusts b to do x”.

Following the views of Cook and Hardin, this study argues that trust involves the trustor (a), the trustee (b), and the entrusted matter (x). On this basis, the study places greater emphasis on the trust intention in specific situations, highlighting the joint influence of relationship type, the entrusted matter, and trustee attributes on trust. Therefore, trust in this study is defined as the trust between a trustor and a trustee regarding a specific matter within a particular relationship. In other words, trust in this study actually includes four core dimensions: trustor (a), trustee (b), relationship type (R), and entrusted matter (x) (J. Liu & Guo, 2024; J. Liu & Li, 2025).

The trustor may be a government, an organization, or a leader, but in most cases refers to a general person. The trustee can also be an individual, an organization, or a government. The entrusted matter can take various forms, such as lending money, conveying information, or transferring rights. Relationship types may involve family members, friends, colleagues, neighbors, and other social ties. Therefore, trust can manifest in many different forms, which also implies that its concretization and operationalization are inherently complex.

To make the above conceptual framework empirically testable, trust needs to be further operationalised into measurable patterns. Based on the dimensions discussed above, the operational model of trust can be expressed as:

T(ab) ⇐ a (economic capacity, sense of security, social value orientation, etc.) × b (ability, degree of integrity, etc.) × x (large or small loan amount) × R(ab) (kin, friends, or business partners).

This model suggests that trust is shaped by the interaction of multiple factors, including the trustor, the trustee, the relationship type, and the importance of the entrusted matter. Specifically, trust intention is influenced by various factors, such as the characteristics of the trustor and the trustee (including cognitive ability, personal experience, values, personal qualities, financial status), the relationship type between the two parties (such as friend, colleague, relative, business partner), the specific context in which they are situated (such as culture, institutions, social environment), and the entrusted matter itself (such as lending money, keeping a secret).

It should be noted that the formation of trust is influenced not only by foundational dimensions such as relationship type, entrusted matter, and trustee attributes, but also by broader social factors including social norms, role expectations, and identity. In modern society, individual identities are multiple and contextually fluid. Interpersonal relationships often simultaneously contain elements of Gemeinschaft, which are based on emotional bonds, and Gesellschaft, which are based on contractual relations and functional differentiation (Tönnies, 2001). Different social structures also shape individuals’ expectations of others and forms of social solidarity (Durkheim & Halls, 1984). Accordingly, individuals may assume different role identities across social contexts, and these identities further influence behavioral norms, interaction expectations, and evaluative standards (Stryker, 1980). The same individual may therefore be assigned different levels of trust across different relational domains.

The relationship type, loan amount, and trustee attributes examined in the present study primarily focus on foundational dimensions of trust judgment within lending contexts. Future research should further incorporate social structure, identity salience, and multiple relational contexts into the analytical framework to more comprehensively understand the mechanisms underlying the formation and transformation of trust in complex social environments.

### 2.3. Research Hypotheses

Trust in Chinese society is closely connected to social relationships and is always embedded in specific relational contexts. Liang (2021) argued that China is a typical “ethics-oriented” society, in which family ethics constitute the starting point of social relations and extend outward to form broader networks of affective ties. Fei (1992, pp. 21–22) introduced the concept of the “differential mode of association” to characterize the structure of Chinese social relations. He compared this structure to ripples spreading outward in water, with the self at the center and concentric circles representing different degrees of intimacy. Different moral principles and behavioral norms apply within different circles. Yang (1995) suggested that Chinese people tend to place unconditional trust in their family while showing relatively low levels of trust toward others. This culturally embedded relationship orientation shapes the logic through which interpersonal trust is formed in Chinese society. Accordingly, this study proposes:

**Hypothesis** **1.** *Under the same entrusted matter, trust intention between the trustor and the trustee varies according to relational closeness. The closer the relationship type, the higher the level of trust intention exhibited by the trustor.*

In addition to relationship type, the entrusted matter also influences the trust intention. In real life, when the entrusted matter is relatively unimportant, the potential loss is limited. Under such conditions, trustors are more likely to base their decisions on relational closeness or the trustee’s personal attributes. In contrast, when the entrusted matter is highly important, the risks and potential losses associated with default increase substantially. In such situations, even when dealing with a closely related trustee, trustors may refuse to entrust out of risk avoidance considerations. For example, trustors are usually willing to lend small amounts of money to friends, but they may refuse when the requested amount becomes very large. Based on this reasoning, this study proposes:

**Hypothesis** **2.** *Within the same relationship type, the greater the importance and risk of the entrusted matter, the lower the level of trust intention that the trustor places in the trustee.*

Trust is not determined by a single factor but results from the interaction of variables. Existing studies have shown that relational resources can reduce uncertainty and promote trust, but their effects do not remain constant across different risk situations. In low-risk situations, potential losses are limited, and individuals are generally more willing to trust others. As a result, the marginal effect of relationship type is relatively weak. Conversely, when the entrusted matter involves extremely high risk, the potential loss may exceed the protection provided by relational norms and reciprocal obligations. Under such conditions, individuals are more likely to rely on risk avoidance considerations when making decisions, which weakens the effect of relationship type (Adobor, 2006; Uzzi, 1997). In contrast, in moderate-risk situations, relational resources may effectively reduce uncertainty without being offset by excessive risk costs. Therefore, the effect of the relationship type may become more pronounced. However, existing research has not yet reached a consistent conclusion on whether strong relationships strengthen or weaken trust under high-risk conditions. Therefore, this nonlinear pattern still requires further empirical testing. Based on this reasoning, this study proposes:

**Hypothesis** **3.** *Relationship type and the importance of the entrusted matter interact to influence trust intention.*

**Hypothesis** **3a** **(Exploratory hypothesis).** *The importance of the entrusted matter moderates the effect of the relationship on trust. Specifically, the effect of relational distance on trust is most pronounced in moderate-risk matters, weaker in low-risk matters, and relatively weakest in high-risk matters. Because the theoretical basis of this pattern still requires further empirical validation, this study presents H3a as an exploratory hypothesis and encourages future research to replicate and further examine this finding.*

Beyond relationship type and the importance of the entrusted matter, scholars have argued that individuals’ perceptions and evaluations of others’ trustworthiness play a crucial role in shaping interpersonal trust (Lewicki et al., 2006). Butler (1991) identified ten factors that influence trust, including openness, acceptance, availability, fairness, loyalty, promise keeping, integrity, competence, consistency, and coherence. Clark and Payne (2006) later summarized these factors into four core dimensions: ability, integrity, fairness, and openness. However, the most influential framework is the integrative trust model proposed by Mayer et al. (1995), which conceptualizes the trustee’s trustworthiness along three dimensions: ability, integrity, and benevolence. Based on this framework, this study proposes:

**Hypothesis** **4.** *Trustee ability and integrity significantly influence the trustor’s trust intention. Compared with trustees characterized by low ability and low integrity, trustees with high ability and high integrity are more likely to earn the trustor’s trust.*

It is important to note that relationship type and the importance of the entrusted matter not only directly influence trust intention, but may also indirectly shape trust intention by affecting how trustors perceive and evaluate trustee attributes. For example, using implicit association tests, some scholars have found significant differences in Chinese participants when associating positive emotional words with different relational targets such as the “self”, “acquaintances”, and “strangers”. Associations with the self and close kin were the fastest, followed by acquaintances, whereas associations with strangers were the slowest (Yuan & Guo, 2017).

Moreover, the importance of the entrusted matter may further moderate these cognitive processes. Under high-risk conditions, trustors are likely to evaluate trustee attributes more cautiously, and the cognitive biases generated by relational closeness may thus be either strengthened or weakened. These suggest that relationship type, entrusted matter, and trustee attributes do not operate independently. Instead, they jointly shape trust intention through dynamic interactions. Accordingly, we propose:

**Hypothesis** **5.** *Relationship type, the importance of the entrusted matter, and trustee attributes interact to jointly influence trust intention.*

## 3. Research Methods

This study employs a situational trust experiment to simulate common entrustment and lending scenarios in everyday life. It systematically examines variations in trust intention across multiple dimensions, including relationship type (kin, acquaintances, and general others), trustee attributes (ability and integrity), and the importance of entrusted matters (loan amount).

### 3.1. Method Selection and Experimental Design

Survey experiments place participants in hypothetical scenarios through carefully designed questionnaires and introduce interventions to examine relationships among variables. In this study, the experimental scenario involves trustees with different relationship types requesting to borrow money from the trustor. Multiple situational conditions are constructed through questionnaire design to implement experimental manipulations. A multi-factor within-subject design is adopted to examine the effects of relationship type, the importance of the entrusted matter, and trustee attributes on trust intention. All participants are required to complete multiple situational tasks across different levels of relational closeness, which enables systematic manipulation and comparison of the independent variables.

To measure relational closeness, this study draws on the concentric circle method proposed by Zhu et al. (2021)^3^, with appropriate modifications to fit the research purpose. Other items are situationally adapted from the classic CGSS question “Do you trust others?” Specifically, the item is operationalized as follows: “If b is your kin, friend, or general other, and b has low/high ability (presented through specific scenarios) and low/high integrity, would you be willing to lend money to b when b requests either a small or a large loan amount?”.

**Figure 1:**
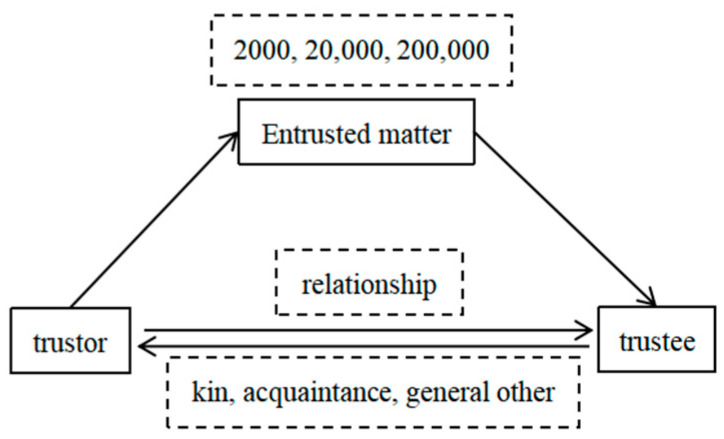
Three-Factor Experimental Design.

The questionnaire consisted of four sections. Section 1 collected demographic information, including gender, age, religious belief, and educational level. Section 2 involved scenario priming and the measurement of relational closeness. During the priming stage, participants were asked to recall one kin, one acquaintance, and one general other, and to record the corresponding terms of address. Subsequently, participants used the concentric circle relationship scale to indicate the positions of these three individuals within their personal relational network centered on themselves. Section 3 measured general trust intention. Participants were asked directly: “Would you be willing to lend the following amounts of money to your kin, acquaintance, or a general other? Please select ‘yes’ or ‘no’.” The loan amounts were set at RMB 2000, RMB 20,000, and RMB 200,000. Section 4 measured trust intention under specific scenarios. By manipulating trustee attributes (ability and integrity) and the entrusted amount (RMB 2000, RMB 20,000, and RMB 200,000), this section examined variations in trust across different situations. For example: “Your acquaintance has a stable job, earns a fixed monthly income, and has never defaulted on a loan. Would you be willing to lend him RMB 20,000?” A total of 18 distinct scenarios were presented, and participants responded by selecting either “yes” or “no”. The experimental design is illustrated in Figure 1.

Regarding the presentation order of the scenarios, this study adopted a fixed blocked design in which scenarios were presented sequentially according to relationship type. The main reason for using a fixed order was that complete randomization could require participants to switch frequently between different relationship types, which might disrupt the stable activation of relationship-related psychological frameworks and thereby weaken the effectiveness of the manipulation of relationship type, the core independent variable in this study. In contrast, the blocked design allowed participants to complete all judgments within the same relationship type under a relatively stable relational psychological representation.

However, the use of a fixed order may introduce certain order effects, such as fatigue effects or practice effects, which constitute a methodological limitation of this study. To partially address this problem, two measures were adopted. First, a pilot study involving 20 participants was conducted before the formal experiment. Based on the pilot results, the wording of questionnaire items and scenario descriptions was refined to improve consistency in task comprehension. Second, attention check items were embedded in the questionnaire to identify and exclude mechanical responses. Future research could further adopt randomized designs or Latin square designs to more rigorously control for order effects and improve external validity.

### 3.2. Variable Measurement and Data Collection

In this study, variables were measured primarily by manipulating the relationship between the trustor and the trustee, trustee attributes, and the importance of the entrusted matter.

(1) Relationship (R)

Interpersonal relationships vary in terms of closeness, and trust levels differ accordingly across relationship types. Previous studies have offered simplified typologies. For example, Chen et al. (2013) classified Chinese interpersonal relationships into three broad categories based on relational closeness: kin, acquaintances, and strangers. Hwang (1987) proposed a threefold typology of affective, mixed, and instrumental relationships, corresponding respectively to kin, acquaintance, and stranger relationships.

Drawing on these perspectives, this study classifies interpersonal relationships into three types according to the degree of closeness. Because this study focuses on trust embedded within minimally established social relationships, the three relationship types are defined as follows: (1) Kin ties: lineal relatives, including parents, siblings, and children. (2) Acquaintance ties: non-blood-related individuals with stable social interactions and emotional bonds, such as friends, teachers, colleagues, classmates, and other collateral relatives. (3) General other ties: weak-tie individuals encountered in daily work or study, involving occasional interaction but lacking deep emotional investment, such as colleagues or classmates with limited contact.

(2) Entrusted Matters (x)

As an important component of trust, the characteristics of entrusted matters, such as their type, importance, and risk level, directly influence the trustor’s trust intention and willingness to entrust. However, research on entrusted matters within trust remains relatively limited. Some scholars have discussed the significance of entrustment, arguing that entrustment involves the trustor voluntarily transferring certain rights or interests to others (Guo, 2016, p. 40). Others have categorized entrusted matters, suggesting they typically involve tangible items, tasks, or affairs, and in some cases may also involve matters related to life or emotions, or psychological well-being (J. Liu & Li, 2025). However, neither of these studies provides an operational framework for measuring entrusted matters.

In the experimental design of this study, two key challenges regarding entrusted matters must be addressed. First, there are substantial individual differences in the perception of the importance and risk of entrusted matters. What may be highly important for one individual may be relatively unimportant for another, making objective measurement difficult. For example, “taking care of a pet” may involve different importance and risk for pet owners and non-pet owners. Second, different entrusted matters often lack comparability. For instance, it is challenging to objectively determine whether “helping take care of a puppy” or “storing important documents for someone” involves greater importance or risk.

To reduce these validity concerns and enhance measurement operability, this study standardizes the entrusted matter as a monetary loan, with the amount manipulated to represent varying levels of importance and risk. This approach offers two main advantages. First, variations in loan amounts (2000 RMB, 20,000 RMB, and 200,000 RMB) directly correspond to a clear risk gradient. Second, money serves as a universal measure of value, which helps reduce the influence of individual preference differences on trust intention. Although this design cannot eliminate all validity concerns, it provides notable benefits in terms of experimental control and result comparability, offering a practical framework for scenario-based measurement of trust intention.

(3) Trustee Attributes (b)

The trustor’s trust intention is also influenced by the personal attributes of the trustee. The construction of attribute dimensions needs to balance theoretical comprehensiveness and experimental feasibility. In prior research, Mayer et al. (1995) proposed an integrative trust model, categorizing trust into ability, benevolence, and integrity. However, scholars differ in how they define “integrity” and “benevolence”. For example, some view integrity as objective honesty and benevolence as subjective goodwill (G. Xu, 2001), while others interpret benevolence as a form of practical rationality manifested in goodwill, honesty, and trustworthiness (Um, 2024). Despite these theoretical differences, integrity and benevolence are closely linked.

In empirical practice, benevolence is often regarded as an internal psychological motivation that is inferred from observable behaviors covered by integrity, such as honesty or keeping promises. As a result, these two dimensions tend to overlap in psychological representation and scale measurement, making them difficult to distinguish statistically. For example, in their review of empirical progress on the integrative model of trust, Schoorman et al. (2007) explicitly noted that research conducted in laboratory settings tends to show high correlations between benevolence and integrity because these relationships have not had time to develop real data on benevolence. By contrast, in long-term field relationships, benevolence and integrity are more likely to emerge as distinguishable dimensions.

This study adopts a short-term, scenario-based, within-subjects experimental design in which participants complete all scenario judgments in a single questionnaire session. This represents a typical research context in which benevolence information has not yet fully developed. In such contexts, maintaining separate measurements for benevolence and integrity is not feasible. Based on these considerations, this study integrates benevolence into the integrity dimension to simplify the variable structure and maintain measurement consistency.

Furthermore, this study focuses on ability and integrity as the two core dimensions of trustee attributes for several reasons. First, these two dimensions represent the fundamental conditions of trust and apply to most trust scenarios. Ability can be measured using objective indicators such as professional background, educational attainment, and professional certifications, while integrity can be evaluated through past behavioral records, such as credit scores or past defaults. Based on this, the trustee’s attributes are classified into two categories: high-level attributes (high ability, high integrity) and low-level attributes (low ability, low integrity), which serve as the experimental manipulation variables^4^.

After designing the experimental questionnaire, this study used G*Power 3.1.9.7 to estimate the required sample size. The analysis was set as an F-test for multiple ANOVA (Fixed effects, special, main effects and interactions) with a medium effect size (Cohen’s f = 0.25), statistical power of 0.8, and a significance level of 0.05. Based on the levels of the three main independent variables in the experimental design (2 × 3 × 3), the degrees of freedom (df) were set to 4. The computation indicated a minimum required sample size of 197. Considering that social science surveys typically contain approximately 10% to 20% invalid responses, this study additionally reserved 20% of the sample size to ensure an adequate number of valid cases.

Data were collected through the Credamo platform, and a total of 325 questionnaires were returned. After quality control procedures, including the exclusion of responses with insufficient completion time, incorrect answers to attention check items, and patterned responding, 252 valid samples were retained, yielding an effective response rate of 77.5%, which meets the statistical power requirement calculated by G*Power.

The demographic characteristics of the sample are presented in Table 1. Among the participants, 56.3% were female, and 43.7% were male. The majority of participants were between 25 and 54 years old (66.7%). In terms of educational attainment, 41.0% had a high school education or below, whereas 46.0% held a bachelor’s degree or above. The occupational distribution included students (21.8%), freelancers or flexible workers (21.4%), and private sector employees (20.2%). Monthly household income was mainly concentrated between RMB 1000 and RMB 5000 (53.9%). Geographically, participants from the Central Inland region accounted for 54.8% of the sample, followed by those from the Eastern Coastal region (27.4%), the Northeast region (10.7%), and the Western region (7.1%).

The sample is relatively diverse in terms of age, occupation, income, and geographic distribution. However, it remains predominantly composed of urban young and middle-aged adults, with limited representativeness of rural and elderly populations. This limitation is addressed further in the discussion section.

## 4. Empirical Results

This study used SPSS 27.0 to analyze the experimental data. First, data cleaning and preprocessing were performed on the valid sample, including variable coding and the treatment of missing values and outliers. Subsequently, the Friedman test and generalized estimating equations (GEE) were employed to systematically examine the hypotheses proposed in this study.

### 4.1. Manipulation Check of Relationship Classification

We classified the relationship type between the trustor and trustee into three types: kin, acquaintance, and general others. To assess whether the trustor’s perceived closeness differs significantly across these relationship types, the Friedman test was used to compare the relational closeness scores among the three groups (higher scores indicate lower perceived closeness). Furthermore, Kendall’s W coefficient was calculated to assess intergroup consistency and effect size. The statistical results are presented in Table 2.

The results revealed significant differences in perceived intimacy across the relationship types (χ^2^(2) = 224.55, *p* < 0.001). The Kendall’s W coefficient of 0.446 indicates a moderate level of agreement among participants in rating the three relationship types. Further comparison of mean ranks reveals that perceived intimacy decreases in the following order: kin (mean rank = 1.36) > acquaintance (mean rank = 1.99) > general others (mean rank = 2.65).

These findings suggest that the relationship classification in this study exhibits clear hierarchical distinctions in perceived intimacy, consistent with theoretical expectations, and thus demonstrates satisfactory manipulation validity. Moreover, the results provide a robust measurement basis for subsequent analyses of the effects of relationship type on trust intention.

### 4.2. Analysis of Main Effects and Interaction Effects

To examine the main effects, two-way interactions, and three-way interactions among relationship type (R), entrusted matter (x), and trustee attributes (b) on trust intention, this study employed Generalized Estimating Equations (GEE). The dependent variable was trust intention measured as hypothetical lending willingness (1 = willing, 0 = unwilling), modeled using a binomial distribution with a logit link function.

The model included the main effects of relationship type (kin, acquaintance and general other), entrusted matter (RMB 2000, RMB 20,000 and RMB 200,000), and trustee attributes (high attributes and low attributes), all two-way interactions (R × x, R × b, x × b), and the three-way interaction (R × x × b). The category “general other × RMB 200,000 × low attributes” served as the reference group. Participant ID was specified as the subject variable, an AR(1) working correlation matrix was assumed, and robust standard errors were used for parameter inference. Control variables included income, gender, and age. The main effect tests are presented in Table 3.

The tests of model effects showed that the main effects of the three core independent variables were all significant: relationship type (Wald χ^2^ = 195.38, *p* < 0.001), entrusted matter (Wald χ^2^ = 262.16, *p* < 0.001), and trustee attributes (Wald χ^2^ = 249.02, *p* < 0.001). All two-way interactions (R × x, R × b, x × b) and the three-way interaction (R × x × b) were also significant (*p* < 0.001). Among the control variables, age was significant at the 0.05 level (Wald χ^2^ = 4.56, *p* = 0.033), whereas income and gender were not significant (*p* > 0.05).

(1) Main Effects Tests

To present the substantive effects of each factor more clearly, Table 4 reports the predicted probabilities (estimated marginal probabilities) at different levels and the marginal effects from pairwise comparisons.

The results in Table 4 show that relationship type had a significant effect on trust intention (Wald χ^2^(2) = 195.38, *p* < 0.001). The estimated marginal probabilities indicate that trust intention was highest toward kin (M = 0.61, 95% CI [0.54, 0.68]), followed by acquaintances (M = 0.35, 95% CI [0.29, 0.42]), and lowest for general others (M = 0.18, 95% CI [0.14, 0.23]). Further pairwise comparisons revealed that, compared with general others, kin increased the probability of trust by an average of 43 percentage points (Δ*p* = +0.43, 95% CI [0.37, 0.50], *p* < 0.001). Compared with acquaintances, kin increased trust intention by 26 percentage points (Δ*p* = +0.26, 95% CI [0.21, 0.32], *p* < 0.001). These findings indicate that the closer the relationship, the stronger the trustor’s trust intention toward the trustee, thereby supporting Hypothesis 1.

Entrusted matter also significantly affected trust intention (Wald χ^2^(2) = 262.16, *p* < 0.001). As the loan amount increased, the probability of trust intention decreased substantially. The predicted probability of trust intention was 0.67 (95% CI [0.60, 0.73]) for RMB 2000, 0.37 (95% CI [0.31, 0.44]) for RMB 20,000, and 0.13 (95% CI [0.10, 0.18]) for RMB 200,000. Pairwise comparisons showed that the probability of trust intention was 53 percentage points higher for RMB 2000 than for RMB 200,000 (Δ*p* = +0.53, 95% CI [0.46, 0.60], *p* < 0.001). Compared with RMB 200,000, the probability of trust intention for RMB 20,000 was 24 percentage points higher (Δ*p* = +0.24, 95% CI [0.18, 0.29], *p* < 0.001). In addition, the probability of trust intention for RMB 2000 was 30 percentage points higher than that for RMB 20,000 (Δ*p* = +0.30, 95% CI [0.25, 0.35], *p* < 0.001). These results indicate that the higher the risk associated with the entrusted matter, the lower the individual’s trust intention, thus supporting H2.

In addition, trustee attributes also significantly influenced trust intention (Wald χ^2^(1) = 249.02, *p* < 0.001). The predicted probability of trust intention under the high attributes condition was 0.59 (95% CI [0.52, 0.65]), which was significantly higher than that under the low attributes condition (M = 0.18, 95% CI [0.14, 0.23]). Pairwise comparisons further showed that the high attributes condition increased the probability of trust by an average of 41 percentage points (Δ*p* = +0.41, 95% CI [0.36, 0.45], *p* < 0.001). Therefore, the higher the trustee’s attributes, the stronger the trustor’s trust intention, thus supporting H4.

(2) Two-Way Interaction Effects

To present the practical effect sizes under each condition, Table 5 reports the predicted probabilities and marginal effects for different combinations of relationship type and entrusted matter.

The GEE results showed a significant interaction between relationship type and entrusted matter (Wald χ^2^(4) = 27.157, *p* < 0.001), indicating that the effect of relational closeness on trust intention varies with the loan amount. Thus, the moderating effect of the entrusted matter on the relational effect is supported (H3).

Simple effect analyses revealed the following. Under the low risk condition (RMB 2000), the predicted probabilities of trust intention for kin, acquaintances, and general others were 0.83 (95% CI [0.78, 0.88]), 0.71 (95% CI [0.63, 0.77]), and 0.40 (95% CI [0.33, 0.48]), respectively. Compared with general others, kin increased the probability of trust intention by 43 percentage points (Δ*p* = +0.43, 95% CI [0.36, 0.50], *p* < 0.001), whereas acquaintances increased it by 30 percentage points (Δ*p* = +0.30, 95% CI [0.23, 0.37], *p* < 0.001).

Under the moderate-risk condition (RMB 20,000), the predicted probabilities of trust intention declined to 0.63 (95% CI [0.56, 0.70]) for kin, 0.36 (95% CI [0.29, 0.44]) for acquaintances, and 0.17 (95% CI [0.13, 0.22]) for general others. Relative to general others, family members raised the lending probability by 47 percentage points (Δ*p* = +0.47, 95% CI [0.39, 0.54], *p* < 0.001), and acquaintances raised it by 19 percentage points (Δ*p* = +0.19, 95% CI [0.14, 0.25], *p* < 0.001).

**Figure 2:**
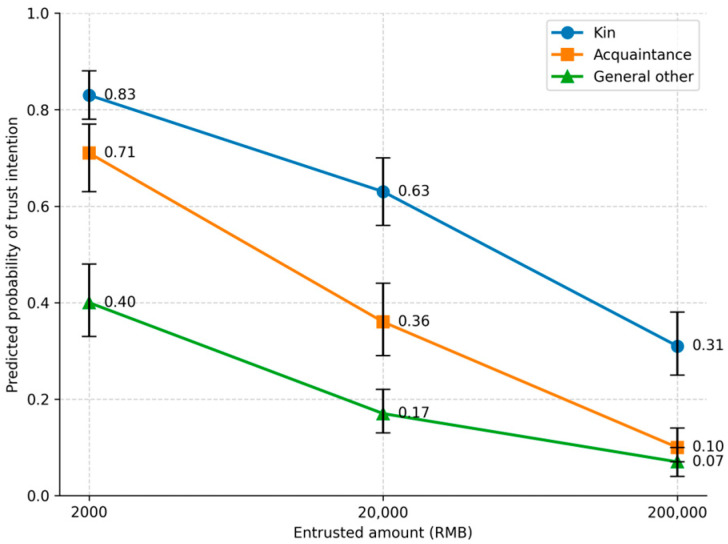
Interactive effect of relationship type and loan amount on trust intention.

Under the high-risk condition (RMB 200,000), the predicted probabilities of expressing trust intention further declined to 0.31 (95% CI [0.25, 0.38]) for kin, 0.10 (95% CI [0.07, 0.14]) for acquaintances, and 0.07 (95% CI [0.04, 0.10]) for general others. At the same time, the trust advantage associated with close relationships weakened substantially. Compared with general others, kin increased the probability of trust intention by 24 percentage points (Δ*p* = +0.24, 95% CI [0.17, 0.31], *p* < 0.001), and acquaintances increased it by 4 percentage points (Δ*p* = +0.04, 95% CI [0.01, 0.06], *p* < 0.001). The overall pattern is illustrated in Figure 2.

Overall, as the loan amount increased from RMB 2000 to RMB 200,000, the predicted probability of trust intention decreased markedly across all relationship types, indicating that higher risk significantly suppresses trust intention. Nevertheless, a stable hierarchical pattern remained. Trust intention was consistently highest toward kin, followed by acquaintances, and lowest toward general others. These findings suggest that relational closeness remains an important factor in trust decisions.

**Figure 3:**
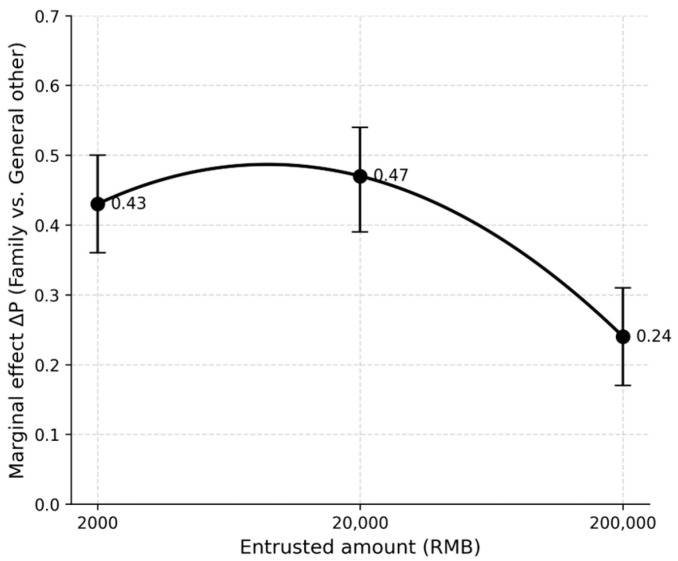
Relationship effect (kin vs. general others) across loan risk levels.

Regarding the exploratory hypothesis H3a, which predicted that the relational effect would be most pronounced under moderate risk, the results showed that the marginal difference between kin and general others was largest under the moderate risk condition (Δ*p* = +0.47), and substantially lower under the low-risk condition (Δ*p* = +0.43), and dropped markedly under the high risk condition (Δ*p* = +0.24). This pattern is consistent with the non-linear trend predicted by H3a (see Figure 3).

However, the marginal effect differed by only four percentage points between the low-risk and moderate-risk conditions, and the corresponding 95% confidence intervals overlapped substantially ([0.36, 0.50] vs. [0.39, 0.54]). This indicates limited statistical evidence for distinguishing between the two conditions. Therefore, Hypothesis 3a receives only preliminary support at the trend level. Future research could increase the number of risk gradients, expand the sample size, or employ continuous risk manipulation to further examine the conditions under which the relational effect reaches its peak.

(3) Three-Way Interaction Test

To present the substantive effects of the three-way interaction, Table 6 reports the predicted probabilities and marginal effects for different combinations of relationship type, entrusted matter, and trustee attributes.

The three-way interaction analysis revealed a significant interaction among relationship type, entrusted matter, and trustee attributes (Wald χ^2^(4) = 36.11, *p* < 0.001). Overall, the probability of trust intention was significantly higher under the high attribute condition than under the low attribute condition, indicating that positive attributes effectively enhance trust intention. However, the effect of trustee attributes was not independent. Instead, it further moderates how the relational effect varies across risk levels.

**Figure 4:**
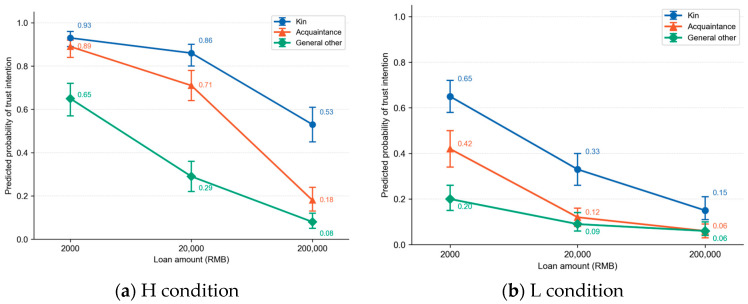
Marginal effect of kin across loan-risk levels under different trustee-attribute conditions (H vs. L).

Under the high attribute condition, the relationship effect followed a non-linear pattern, first increasing and then decreasing as risk increased. The marginal difference between kin and general others increases from 28 percentage points under low risk to 57 percentage points under moderate risk, then falls to 46 percentage points under high risk. The difference between acquaintances and general others showed a similar trend (see Figure 4). This finding suggests that positive trustee attributes and close relationships produce a synergistic effect under moderate risk, jointly promoting trust intention.

Under the low attribute condition, the relational effect declined steadily as risk increased. The difference between kin and general others dropped from 45 percentage points under low risk to 24 percentage points under moderate risk, and further to 9 percentage points under high risk. The difference between acquaintances and general others was even more pronounced, it decreased from 22 percentage points to 2 percentage points and became negligible under the high-risk condition (Δ*p* = +0.00, 95% CI [−0.03, 0.02], *p* = 0.991).

These findings indicate that when high risk is combined with low trustee attributes, the positive effect of relational resources almost completely disappears. Therefore, relationship type, trustee attributes, and entrusted matter importance jointly shape a situated mechanism of trust intention. Thus, Hypothesis 5 is supported.

## 5. Results and Discussion

This study manipulated three variables: relationship type, trustee attributes, and the importance of the entrusted matter, to examine the trust intention of trustors in different situations. The results show that trust is not determined by a single factor. Instead, it is jointly shaped by relational closeness, trustee attributes, and the entrusted matter, demonstrating a clear situational characteristic.

At the main effect level, trust followed a differential pattern: kin > acquaintances > general others. As entrusted matter importance increased, trust intention significantly decreased. High trustee attributes significantly raised the probability of trust.

Regarding interaction effects, there was a significant interaction between relationship type and loan amount, indicating that the relational effect varies with risk level. The three-way interaction further showed that trustee attributes significantly moderate the relationship-risk interaction pattern. Under the high competence and high integrity conditions, the relational effect peaked at moderate risk. Under the low competence and low integrity condition, the relational effect declined steadily as risk increased, approaching zero under high risk. These findings suggest that relationship, trustee attributes, and risk level jointly constitute a situated mechanism of trust judgment.

The following limitations should be addressed in future research.

### 5.1. Ecological Validity

This study used lending money as the operational form of the entrusted matter and distinguished risk levels by loan amount (RMB 2000, 20,000, 200,000). Although money, as a universal measure of value, has strong objectivity and comparability, this design also has limitations in ecological validity.

First, the loan amount reflects not only entrusted matter importance or risk level, but may also involve factors such as affordability, liquidity, and moral expectations. The same amount could imply very different risk perceptions for participants with different income levels and financial capacities. This study did not directly measure participants’ subjective perceptions of risk regarding the three amounts and thus did not provide manipulation check evidence for the amount manipulation. Future research should add manipulation check items after the experiment, such as “How risky do you think this loan would be for you?”, and measure subjective financial capacity to more rigorously assess the validity of the amount manipulation.

Second, the lending scenario is only one type of daily entrusted matter. Whether the relationship-risk-attribute interaction pattern revealed in this study can be generalized to other entrusted matters, such as keeping secrets, seeking emotional support, or entrusting important documents, requires future research using more diverse entrusted scenarios.

### 5.2. Cross-Cultural Generalizability

The sample of this study was drawn entirely from China, and the core theoretical construct is explicitly culture-specific, rooted in the relational logic and particularistic norms of Chinese society. Therefore, generalizing the findings to contexts outside China or to other cultures requires careful distinction.

To some extent, the non-linear interaction pattern between relational closeness and risk level may possess cross-cultural applicability, as it aligns with general theories of social capital, relational embeddedness, and risk perception in Western trust research. The basic phenomenon that individuals allocate trust differently across varying relational distances and risk situations is not unique to Chinese society and thus holds some value for cross-cultural replication.

However, the more culture-specific aspects identified in this study require careful consideration. The concentric circle structure of relational ties described by the differential mode of association, along with the differential moral obligations and particularistic reciprocity, is deeply embedded in the Confucian cultural tradition and differs substantially from the universalistic trust norms prevalent in Western societies. The strong and stable trust advantage associated with kin in this study may be considerably weaker in individualistic cultures or societies with stronger contractual trust norms. Future research should adopt cross-cultural comparative designs, for example, by selecting samples from China, the United States, Germany, or Japan, to examine the boundary conditions of the trust mechanisms revealed in this study.

### 5.3. Behavioral Validity

The dependent variable in this study was participants’ reported trust intention, measured as hypothetical lending willingness, rather than actual lending behavior. In hypothetical scenarios, participants do not bear real economic losses, and the cost of making a decision is virtually zero. This may lead their reported trust intentions to be systematically higher than their trust levels in real behavior (Gneezy & Rustichini, 2000). Moreover, social desirability effects may lead participants to overreport trust intentions when facing close relationships, thereby artificially inflating the estimated relational effect. Therefore, the conclusions of this study should be understood as reflecting cognitive tendencies and intentional patterns of trust judgment rather than direct predictions of real-world lending behavior. Future research could combine incentive-compatible experimental economics paradigms, such as trust games, or natural experimental designs, to more rigorously test whether the mechanisms identified in this study operate similarly in actual behavior.

### 5.4. Sample Representativeness

The participants were recruited through the Credamo online platform. The sample contained relatively high proportions of students (21.8%) and freelancers or flexible workers (21.4%). These two groups may differ systematically from the general population in income stability and risk perception, which may in turn affect their subjective evaluation of the risk levels associated with the loan amounts. In addition, the sample was predominantly from central inland regions (54.8%) and comprised mainly urban young and middle-aged adults, with limited representation of rural residents, elderly populations, and residents of northeast and western regions. Future research could use probability sampling or quota sampling to achieve more balanced geographical and demographic distributions, thereby improving the external validity of the findings.

Finally, this study focused on three independent variables, while other factors that may also influence trust were not incorporated into the analytical framework. These factors include moral emotions, responsibility attribution, and the internalization of social norms. In particular, the ethical obligations and emotional bonding underlying trust in family relationships were not fully explored. Future research could further incorporate these variables to build a more comprehensive model of trust. In addition, this study combined the trustee’s benevolence and integrity into a single “integrity” dimension. This operationalization may sacrifice construct distinctiveness and thus represents a limitation of the present study. Future research could measure benevolence and integrity separately in long-term field studies or longitudinal data to examine their independent effects.

## Figures and Tables

**Table 1:** Descriptive statistics of demographic variables.

Variables	Category	Frequency	Percentage	Mean	SD
Gender	Female	142	56.3%	0.44	0.497
	Male	110	43.7%		
Age	Under 18	8	3.2%	3.7579	1.41454
	19–24	43	17.1%		
	25–34	71	28.2%		
	35–44	47	18.7%		
	45–54	50	19.8%		
	55–64	29	11.5%		
	65+	4	1.6%		
Education	High school or below	103	41%	2.21	1.147
	Junior college	33	13%		
	Bachelor’s	75	30%		
	Master’s or above	41	16%		
Major	No college	104	41.3%	1.21	1.299
	Humanities and social sciences	61	24.2%		
	Economics and management	34	13.5%		
	Natural sciences and engineering	36	14.3%		
	Medicine and health	17	6.7%		
Occupation	Student	55	21.8%	3.51	1.771
	Government/institution staff	32	12.7%		
	State-owned/collective enterprise staff	23	9.1%		
	Private company or self-employed	51	20.2%		
	Freelancer/flexible employment	54	21.4%		
	Unemployed/job-seeking	37	14.7%		
Monthly	<1000 RMB	45	17.9%	2.82	1.465
	1000–3000 RMB	82	32.5%		
	3000–5000 RMB	54	21.4%		
	5000–8000 RMB	32	12.7%		
	8000–12,000 RMB	28	11.1%		
	12,000–15,000 RMB	5	2%		
	>15,000 RMB	6	2.4%		
Region	Central Region	138	54.76%	—	—
	Eastern Coast	69	27.38%		
	Northeast Region	27	10.71%		
	Western Region	18	7.14%		

**Table 2:** Multiple Related-Samples Test.

Relationship	Mean Rank	Chi-Square	Kendall’s W	*p*
Kin	1.36	224.553	0.446	0.001
Acquaintance	1.99			
General others	2.65			

Note: The Friedman test was applied to examine whether overall differences existed among the three relationship categories, while Kendall’s W was used to assess the degree of rating consistency.

**Table 3:** Tests of Model Effects.

Variable	Wald χ^2^	*p*
Intercept	14.455	<0.001
Relationship (R)	195.375	<0.001
Loan amount (x)	262.163	<0.001
Trustee attributes (b)	249.017	<0.001
R × x	27.157	<0.001
R × b	38.129	<0.001
x × b	57.703	<0.001
R × x × b	36.107	<0.001
Income	5.308	0.505
Gender	0.023	0.880
Age	4.516	0.033

**Table 4:** Predicted Probabilities and Marginal Effects by Variable Level.

Var	Level	Predicted Probability M [95% CI]	Marginal Effect Δ*p* [95% CI]	Wald $\chi^{2}$	*p*
R	Kin	0.61 [0.54, 0.68]	—	195.38	<0.001
	Acq	0.35 [0.29, 0.42]	—		
	Gen	0.18 [0.14, 0.23]	—		
	Kin vs. Gen	—	+0.43 [0.37, 0.50]		
	Acq vs. Gen	—	+0.17 [0.13, 0.22]		
	Kin vs. Acq	—	+0.26 [0.21, 0.32]		
x	2000	0.67 [0.60, 0.73]	—	262.16	<0.001
	20,000	0.37 [0.31, 0.44]	—		
	200,000	0.13 [0.10, 0.18]	—		
	2000 vs. 200,000	—	+0.53 [0.46, 0.60]		
	20,000 vs. 200,000	—	+0.24 [0.18, 0.29]		
	2000 vs. 20,000	—	+0.3 [0.25, 0.35]		
b	H	0.59 [0.52, 0.65]	—	249.02	<0.001
	L	0.18 [0.14, 0.23]	—		
	H vs. L	—	+0.41 [0.36, 0.45]		

Note. Age was held constant at 37.04 years. Wald χ^2^ statistics represent omnibus tests for each independent variable in the GEE model. When omnibus effects were significant, Bonferroni-adjusted pairwise comparisons were conducted. Δ*p* indicates the difference in estimated marginal probabilities between conditions. R = relationship type; x = loan amount; b = trustee attributes; H = high competence and high integrity; L = low competence and low integrity.

**Table 5:** Interaction Effects of Relationship Type and Loan Amount.

Entrusted Matter	Relationship	Predicted Probability M [95% CI]	Marginal Effect Δ*p* [95% CI]	Wald $\chi^{2}$	*p*
2000	Kin	0.83 [0.78, 0.88]	+0.43 [0.36, 0.50] ^1^	192.18	<0.001
	Acquaintance	0.71 [0.63, 0.77]	+0.30 [0.23, 0.37] ^2^		
	General	0.40 [0.33, 0.48]	—		
20,000	Kin	0.63 [0.56, 0.70]	+0.47 [0.39, 0.54] ^1^	225.17	<0.001
	Acquaintance	0.36 [0.29, 0.44]	+0.19 [0.14, 0.25] ^2^		
	General	0.17 [0.13, 0.22]	—		
200,000	Kin	0.31 [0.25, 0.38]	+0.24 [0.17, 0.31] ^1^	63.76	<0.001
	Acquaintance	0.10 [0.07, 0.14]	+0.04 [0.01, 0.06] ^2^		
	General	0.07 [0.04, 0.10]	—		

Note. Estimated marginal probabilities were calculated based on the GEE model, with age held constant at 37.04 years. ^1^ Kin vs. general others; ^2^ acquaintances vs. general others. Pairwise comparisons were Bonferroni-adjusted. The Wald χ^2^ omnibus effect of relationship type was significant at each loan amount level.

**Table 6:** Three-Way Interaction Effects: Estimated Marginal Probabilities and Marginal Effects.

Entrusted Matter	Trustee Attributes	Relationship	Predicted Probability M [95% CI]	Marginal Effect Δ*p* [95% CI]	Wald $\chi^{2}$	*p*
2000	H	Kin	0.93 [0.89, 0.96]	+0.28 [0.20, 0.36] ^1^	71.50	<0.001
		Acquaintance	0.89 [0.84, 0.92]	+0.24 [0.16, 0.31] ^2^		
		General	0.65 [0.57, 0.72]	—		
	L	Kin	0.65 [0.58, 0.72]	+0.45 [0.37, 0.54] ^1^	169.40	<0.001
		Acquaintance	0.42 [0.34, 0.50]	+0.22 [0.14, 0.30] ^2^		
		General	0.20 [0.15, 0.26]	—		
20,000	H	Kin	0.86 [0.80, 0.90]	+0.57 [0.49, 0.65] ^1^	292.82	<0.001
		Acquaintance	0.71 [0.64, 0.78]	+0.43 [0.35, 0.50] ^2^		
		General	0.29 [0.22, 0.36]	—		
	L	Kin	0.33 [0.26, 0.40]	+0.24 [0.16, 0.31] ^1^	57.36	<0.001
		Acquaintance	0.12 [0.08, 0.16]	+0.02 [0.00, 0.05] ^2^		
		General	0.09 [0.06, 0.14]	—		
200,000	H	Kin	0.53 [0.45, 0.61]	+0.46 [0.36, 0.55] ^1^	131.64	<0.001
		Acquaintance	0.18 [0.13, 0.24]	+0.10 [0.05, 0.16] ^2^		
		General	0.08 [0.05, 0.12]	—		
	L	Kin	0.15 [0.11, 0.21]	+0.09 [0.03, 0.15] ^1^	17.19	<0.001
		Acquaintance	0.06 [0.03, 0.09]	+0.00 [−0.03, 0.02] ^2^		
		General	0.06 [0.04, 0.10]	—		

Note. Estimated marginal probabilities were derived from the generalized estimating equation (GEE) model. Δ*p* indicates the difference in estimated marginal probabilities between groups. ^1^ Kin vs. general others; ^2^ acquaintances vs. general others. Pairwise comparisons were Bonferroni-adjusted.

## Data Availability

We have uploaded a compressed archive (Supplementary Materials) containing all relevant study materials, including the survey questionnaire, raw and processed datasets, and the complete SPSS analysis outputs.

## Notes

1 Abstract: Based on the analysis of the five waves survey from the 2002–2015 data at 40 villages in Jiangxi province, this paper indicates that interpersonal trust of peasants emerged as a U-shaped change and general increase. Meanwhile, the internal structure of peasants trust demonstrates a pattern of difference sequence. The sequence from inside to outside of interpersonal trust of the peasants is family relationship-embedded relationship-open relationship”. This paper also analyzes the influencing factors of interpersonal trust by its degree and range, and the results suggest that the institutional and cultural factors are more applicable for explaining this interpersonal trust than the rational factor. Therefore, the paper advises that to create and improve the interpersonal trust in the rural area, to improve the quality of government governance and to inherit the excellent traditional culture for creating a harmonious interpersonal environment is necessary. In addition, government trust, the impact of traditional culture on the different structures of interpersonal trust is also worthy of subsequent attention.

2 Abstract: Drawing on data from the 2005 and 2015 Chinese General Social Survey, we examine how interpersonal trust has evolved in transitional China as well as its socioeconomic determinants. Results show that the overall interpersonal trust declined slightly among Chinese residents between 2005 and 2015. Nonetheless, this decline was mainly driven by increasing distrust in specialized connections, while generalized trust increased during the same period, indicating a structural shift in interpersonal trust in contemporary China. We also find that socioeconomic factors have a complicated impact oninterpersonal trust within both periods and discuss potential trends and implications.

3 Abstract: Trust is often exercised with immediate or extended family members in China, which implies that relationship closeness could positively affect interpersonal trust. Renging, a set of Chinese social norms to get along well with others, also has a positive influence on trust. Moreover, an individual’s trustworthiness, including ability, benevolence and integrity, are important antecedents of interpersonal trust. Based on extant theories, we attempt to build a systematic mechanism by investigating the influence of relationship closeness, perceived trustworthiness and Renging orientation on interpersonal trust.

4 In the following sections, “H” refer specifically to high ability and high integrity, whereas “L” denote low ability and low integrity.

## Supplementary Materials

The following supporting information can be downloaded at: https://www.mdpi.com/article/10.3390/bs16061001/s1.
