# Supplementary material for: Trust in Context: A Three-Factor Experimental Study
Source: Behav Sci (Basel). 2026 Jun 15;16(6):1001. doi: 10.3390/bs16061001 (PMC13296098; doi:10.3390/bs16061001)
Supplement: Supplementary file 1 [file behavsci-16-01001-s001.zip › Results of the Three-Way Interaction Experiment(English).pdf]

```
*                    .
GENLIN y (REFERENCE=FIRST) BY R x b gender_g income_g (ORDER=ASCENDING) WITH age
 /MODEL R x b gender_g income_g age R*x R*b x*b R*x*b INTERCEPT=YES
 DISTRIBUTION=BINOMIAL LINK=LOGIT
 /CRITERIA METHOD=FISHER(1) SCALE=1 MAXITERATIONS=100 MAXSTEPHALVING=5 PCONVERGE=1E-
006(ABSOLUTE)
  SINGULAR=1E-012 ANALYSISTYPE=3(WALD) CILEVEL=95 LIKELIHOOD=FULL
 /EMMEANS TABLES=R SCALE=ORIGINAL COMPARE=R CONTRAST=PAIRWISE PADJUST=BONFERRONI
 /EMMEANS TABLES=x SCALE=ORIGINAL COMPARE=x CONTRAST=PAIRWISE PADJUST=BONFERRONI
 /EMMEANS TABLES=b SCALE=ORIGINAL COMPARE=b CONTRAST=PAIRWISE PADJUST=BONFERRONI
 /EMMEANS TABLES=R*x SCALE=ORIGINAL COMPARE=R*x CONTRAST=PAIRWISE PADJUST=BONFERRO
NI
 /EMMEANS TABLES=R*b SCALE=ORIGINAL COMPARE=R*b CONTRAST=PAIRWISE PADJUST=BONFERRO
NI
 /EMMEANS TABLES=x*b SCALE=ORIGINAL COMPARE=x*b CONTRAST=PAIRWISE PADJUST=BONFERRON
I
 /EMMEANS TABLES=R*x*b SCALE=ORIGINAL COMPARE=R*x*b CONTRAST=PAIRWISE PADJUST=BONFER
RONI
 /REPEATED SUBJECT=id WITHINSUBJECT=R*x*b SORT=YES CORRTYPE=AR
(1) ADJUSTCORR=YES COVB=ROBUST
  MAXITERATIONS=100 PCONVERGE=1e-006(ABSOLUTE) UPDATECORR=1
 /MISSING CLASSMISSING=EXCLUDE
 /PRINT CPS DESCRIPTIVES MODELINFO FIT SUMMARY SOLUTION (EXPONENTIATED) WORKINGCORR.
```

Generalized Linear Models

Notes

Output Created		11-JUN-2026 13:39:38
Comments		
Input	Data	E : \ 1 . \ 2 .<br>\ 2 . \ 4 .<br>\ 1 .<br>\behavior sciences\<br>\ .<br>sav
	Active Dataset	1
	Filter	<none>
	Weight	<none>
	Split File	<none>
	N of Rows in Working Data File	4536
Missing Value Handling	Definition of Missing	User-defined missing values for factor, subject and within-subject variables are treated as missing.
	Cases Used	Statistics are based on cases with valid data for all variables in the model.
Weight Handling		not applicable

Notes

Syntax	GENLIN y (REFERENCE=FIRST) BY R x b gender_g income_g (ORDER=ASCENDING) WITH age<br>/MODEL R x b gender_g income_g age R*x R*b x*b R*x*b INTERCEPT=YES<br>DISTRIBUTION=BINOMIAL LINK=LOGIT<br>/CRITERIA METHOD=FISHER(1) SCALE=1 MAXITERATIONS=100 MAXSTEPHALVING=5 PCONVERGE=1E-006 (ABSOLUTE)<br>SINGULAR=1E-012 ANALYSISTYPE=3 (WALD) CILEVEL=95 LIKELIHOOD=FULL<br>/EMMEANS TABLES=R SCALE=ORIGINAL COMPARE=R CONTRAST=PAIRWISE PADJUST=BONFERRONI<br>/EMMEANS TABLES=x SCALE=ORIGINAL COMPARE=x CONTRAST=PAIRWISE PADJUST=BONFERRONI<br>/EMMEANS TABLES=b SCALE=ORIGINAL COMPARE=b CONTRAST=PAIRWISE PADJUST=BONFERRONI<br>/EMMEANS TABLES=R*x SCALE=ORIGINAL COMPARE=R*x CONTRAST=PAIRWISE PADJUST=BONFERRONI<br>/EMMEANS TABLES=R*b SCALE=ORIGINAL COMPARE=R*b CONTRAST=PAIRWISE PADJUST=BONFERRONI<br>/EMMEANS TABLES=x*b SCALE=ORIGINAL COMPARE=x*b CONTRAST=PAIRWISE PADJUST=BONFERRONI<br>/EMMEANS TABLES=R*x*b SCALE=ORIGINAL COMPARE=R*x*b CONTRAST=PAIRWISE PADJUST=BONFERRONI<br>/REPEATED SUBJECT=id WITHINSUBJECT=R*x*b SORT=YES

Notes

Resources	Processor Time	00:00:00.49
	Elapsed Time	00:00:01.12

Model Information

Dependent Variable		y[a]
Probability Distribution		Binomial
Link Function		Logit
Subject Effect	1	id
Within-Subject Effect	1	R
	2	x
	3	b
Working Correlation Matrix Structure		AR(1)

a. The procedure models 1 as the response, treating 0 as the reference category.

Case Processing Summary

	N	Percent
Included	4536	100.0%
Excluded	0	0.0%
Total	4536	100.0%

Correlated Data Summary

Number of Levels	Subject Effect	id	252
	Within-Subject Effect	R	3
		x	3
		b	2
Number of Subjects			252
Number of Measurements per Subject	Minimum		18
	Maximum		18
Correlation Matrix Dimension			18

Categorical Variable Information

			N	Percent
Dependent Variable	y	0	2753	60.7%
		1	1783	39.3%
		Total	4536	100.0%
Factor	R	kin	1512	33.3%
		acquaintance	1512	33.3%
		general other	1512	33.3%
		Total	4536	100.0%
	x	2000	1512	33.3%
		20,000	1512	33.3%
		200,000	1512	33.3%
		Total	4536	100.0%
	b	H	2268	50.0%
		L	2268	50.0%
		Total	4536	100.0%
	gender_g	female	2556	56.3%
		male	1980	43.7%
		Total	4536	100.0%
	income_g	1000RMB	810	17.9%
		1000-3000RMB	1476	32.5%
		3000-5000RMB	972	21.4%
		5000-8000RMB	576	12.7%
		8000-12000RMB	504	11.1%
		12000-15000RMB	90	2.0%
		15000RMB	108	2.4%
		Total	4536	100.0%

Continuous Variable Information

		N	Minimum	Maximum	Mean	Std. Deviation
Covariate	age	4536	16	69	37.04	13.196

Goodness of Fit[a]

	Value
Quasi Likelihood under Independence Model Criterion (QIC)[b]	4256.201
Corrected Quasi Likelihood under Independence Model Criterion (QICC)[b]	4199.005

Dependent Variable: y
Model: (Intercept), R, x, b, gender_g, income_g, age, R * x, R * b, x * b, R * x * b

a. Information criteria are in smaller-is-better form.

b. Computed using the full log quasi-likelihood function.

Tests of Model Effects

Source	Type III		
	Wald Chi-Square	df	Sig.
(Intercept)	14.455	1	.000
R	195.375	2	.000
x	262.163	2	.000
b	249.017	1	.000
gender_g	.023	1	.880
income_g	5.308	6	.505
age	4.561	1	.033
R * x	27.157	4	.000
R * b	38.129	2	.000
x * b	57.703	2	.000
R * x * b	36.107	4	.000

Dependent Variable: y
Model: (Intercept), R, x, b, gender_g, income_g, age, R * x, R * b, x * b, R * x * b

Parameter Estimates

Parameter	B	Std. Error	95% Wald Confidence Interval		Hypothesis Test
			Lower	Upper	Wald Chi-Square
(Intercept)	-3.847	.6805	-5.181	-2.514	31.965
[R=1]	1.012	.2594	.503	1.520	15.204
[R=2]	-.071	.2188	-.499	.358	.104
[R=3]	0[a]	.	.	.	.
[x=1]	1.351	.2379	.885	1.818	32.278
[x=2]	.473	.1826	.115	.830	6.700
[x=3]	0[a]	.	.	.	.
[b=1]	.257	.2199	-.174	.688	1.361
[b=2]	0[a]	.	.	.	.
[gender_g=0]	-.027	.1791	-.378	.324	.023
[gender_g=1]	0[a]	.	.	.	.
[income_g=1]	.330	.6365	-.917	1.578	.269
[income_g=2]	.645	.6169	-.564	1.854	1.094
[income_g=3]	.541	.6146	-.663	1.746	.776
[income_g=4]	.593	.6170	-.616	1.802	.923
[income_g=5]	.450	.6252	-.775	1.675	.519
[income_g=6]	1.426	.7794	-.101	2.954	3.349
[income_g=7]	0[a]	.	.	.	.
age	.015	.0068	.001	.028	4.561
[R=1] * [x=1]	1.019	.2724	.485	1.552	13.978
[R=1] * [x=2]	.555	.2184	.127	.983	6.457
[R=1] * [x=3]	0[a]	.	.	.	.
[R=2] * [x=1]	1.139	.2538	.642	1.637	20.151
[R=2] * [x=2]	.314	.1903	-.059	.686	2.716
[R=2] * [x=3]	0[a]	.	.	.	.
[R=3] * [x=1]	0[a]	.	.	.	.
[R=3] * [x=2]	0[a]	.	.	.	.
[R=3] * [x=3]	0[a]	.	.	.	.
[R=1] * [b=1]	1.619	.2879	1.054	2.183	31.609
[R=1] * [b=2]	0[a]	.	.	.	.
[R=2] * [b=1]	1.053	.3275	.411	1.695	10.344
[R=2] * [b=2]	0[a]	.	.	.	.
[R=3] * [b=1]	0[a]	.	.	.	.
[R=3] * [b=2]	0[a]	.	.	.	.

Parameter Estimates

Parameter	Hypothesis Test		Exp(B)	95% Wald Confidence Interval for Exp(B)	
	df	Sig.		Lower	Upper
(Intercept)	1	.000	.021	.006	.081
[R=1]	1	.000	2.750	1.654	4.573
[R=2]	1	.747	.932	.607	1.431
[R=3]	.	.	1	.	.
[x=1]	1	.000	3.863	2.423	6.157
[x=2]	1	.010	1.604	1.122	2.294
[x=3]	.	.	1	.	.
[b=1]	1	.243	1.292	.840	1.989
[b=2]	.	.	1	.	.
[gender_g=0]	1	.880	.973	.685	1.383
[gender_g=1]	.	.	1	.	.
[income_g=1]	1	.604	1.391	.400	4.844
[income_g=2]	1	.296	1.907	.569	6.389
[income_g=3]	1	.378	1.718	.515	5.731
[income_g=4]	1	.337	1.809	.540	6.063
[income_g=5]	1	.471	1.569	.461	5.341
[income_g=6]	1	.067	4.164	.904	19.185
[income_g=7]	.	.	1	.	.
age	1	.033	1.015	1.001	1.028
[R=1] * [x=1]	1	.000	2.769	1.623	4.723
[R=1] * [x=2]	1	.011	1.742	1.135	2.672
[R=1] * [x=3]	.	.	1	.	.
[R=2] * [x=1]	1	.000	3.125	1.900	5.139
[R=2] * [x=2]	1	.099	1.368	.942	1.987
[R=2] * [x=3]	.	.	1	.	.
[R=3] * [x=1]	.	.	1	.	.
[R=3] * [x=2]	.	.	1	.	.
[R=3] * [x=3]	.	.	1	.	.
[R=1] * [b=1]	1	.000	5.046	2.870	8.871
[R=1] * [b=2]	.	.	1	.	.
[R=2] * [b=1]	1	.001	2.867	1.509	5.448
[R=2] * [b=2]	.	.	1	.	.
[R=3] * [b=1]	.	.	1	.	.
[R=3] * [b=2]	.	.	1	.	.

Parameter Estimates

Parameter	B	Std. Error	95% Wald Confidence Interval		Hypothesis Test
			Lower	Upper	Wald Chi-Square
[x=1] * [b=1]	1.760	.2513	1.268	2.253	49.057
[x=1] * [b=2]	0[a]	.	.	.	.
[x=2] * [b=1]	1.116	.2313	.662	1.569	23.265
[x=2] * [b=2]	0[a]	.	.	.	.
[x=3] * [b=1]	0[a]	.	.	.	.
[x=3] * [b=2]	0[a]	.	.	.	.
[R=1] * [x=1] * [b=1]	-1.679	.3720	-2.408	-.950	20.363
[R=1] * [x=1] * [b=2]	0[a]	.	.	.	.
[R=1] * [x=2] * [b=1]	-.465	.3177	-1.087	.158	2.139
[R=1] * [x=2] * [b=2]	0[a]	.	.	.	.
[R=1] * [x=3] * [b=1]	0[a]	.	.	.	.
[R=1] * [x=3] * [b=2]	0[a]	.	.	.	.
[R=2] * [x=1] * [b=1]	-.658	.3874	-1.418	.101	2.886
[R=2] * [x=1] * [b=2]	0[a]	.	.	.	.
[R=2] * [x=2] * [b=1]	.524	.3314	-.125	1.174	2.504
[R=2] * [x=2] * [b=2]	0[a]	.	.	.	.
[R=2] * [x=3] * [b=1]	0[a]	.	.	.	.
[R=2] * [x=3] * [b=2]	0[a]	.	.	.	.
[R=3] * [x=1] * [b=1]	0[a]	.	.	.	.
[R=3] * [x=1] * [b=2]	0[a]	.	.	.	.
[R=3] * [x=2] * [b=1]	0[a]	.	.	.	.
[R=3] * [x=2] * [b=2]	0[a]	.	.	.	.
[R=3] * [x=3] * [b=1]	0[a]	.	.	.	.
[R=3] * [x=3] * [b=2]	0[a]	.	.	.	.
(Scale)	1				

Parameter Estimates

Parameter	Hypothesis Test		Exp(B)	95% Wald Confidence Interval for Exp(B)	
	df	Sig.		Lower	Upper
[x=1] * [b=1]	1	.000	5.815	3.553	9.517
[x=1] * [b=2]	.	.	1	.	.
[x=2] * [b=1]	1	.000	3.051	1.939	4.802
[x=2] * [b=2]	.	.	1	.	.
[x=3] * [b=1]	.	.	1	.	.
[x=3] * [b=2]	.	.	1	.	.
[R=1] * [x=1] * [b=1]	1	.000	.187	.090	.387
[R=1] * [x=1] * [b=2]	.	.	1	.	.
[R=1] * [x=2] * [b=1]	1	.144	.628	.337	1.171
[R=1] * [x=2] * [b=2]	.	.	1	.	.
[R=1] * [x=3] * [b=1]	.	.	1	.	.
[R=1] * [x=3] * [b=2]	.	.	1	.	.
[R=2] * [x=1] * [b=1]	1	.089	.518	.242	1.106
[R=2] * [x=1] * [b=2]	.	.	1	.	.
[R=2] * [x=2] * [b=1]	1	.114	1.689	.882	3.234
[R=2] * [x=2] * [b=2]	.	.	1	.	.
[R=2] * [x=3] * [b=1]	.	.	1	.	.
[R=2] * [x=3] * [b=2]	.	.	1	.	.
[R=3] * [x=1] * [b=1]	.	.	1	.	.
[R=3] * [x=1] * [b=2]	.	.	1	.	.
[R=3] * [x=2] * [b=1]	.	.	1	.	.
[R=3] * [x=2] * [b=2]	.	.	1	.	.
[R=3] * [x=3] * [b=1]	.	.	1	.	.
[R=3] * [x=3] * [b=2]	.	.	1	.	.
(Scale)					

Dependent Variable: y
Model: (Intercept), R, x, b, gender_g, income_g, age, R * x, R * b, x * b, R * x * b

a. Set to zero because this parameter is redundant.

Working Correlation Matrix[a]

Measurement	Measurement: [R = 1]* [x = 1]* [b = 1]	[R = 1]* [x = 1]* [b = 2]	[R = 1]* [x = 2]* [b = 1]	[R = 1]* [x = 2]* [b = 2]
[R = 1]* [x = 1]* [b = 1]	1.000	.349	.122	.043
[R = 1]* [x = 1]* [b = 2]	.349	1.000	.349	.122
[R = 1]* [x = 2]* [b = 1]	.122	.349	1.000	.349
[R = 1]* [x = 2]* [b = 2]	.043	.122	.349	1.000
[R = 1]* [x = 3]* [b = 1]	.015	.043	.122	.349
[R = 1]* [x = 3]* [b = 2]	.005	.015	.043	.122
[R = 2]* [x = 1]* [b = 1]	.002	.005	.015	.043
[R = 2]* [x = 1]* [b = 2]	.001	.002	.005	.015
[R = 2]* [x = 2]* [b = 1]	.000	.001	.002	.005
[R = 2]* [x = 2]* [b = 2]	.000	.000	.001	.002
[R = 2]* [x = 3]* [b = 1]	.000	.000	.000	.001
[R = 2]* [x = 3]* [b = 2]	.000	.000	.000	.000
[R = 3]* [x = 1]* [b = 1]	.000	.000	.000	.000
[R = 3]* [x = 1]* [b = 2]	.000	.000	.000	.000
[R = 3]* [x = 2]* [b = 1]	.000	.000	.000	.000
[R = 3]* [x = 2]* [b = 2]	.000	.000	.000	.000
[R = 3]* [x = 3]* [b = 1]	.000	.000	.000	.000
[R = 3]* [x = 3]* [b = 2]	.000	.000	.000	.000

Working Correlation Matrix[a]

Measurement	Measurement			
	[R = 1]* [x = 3]* [b = 1]	[R = 1]* [x = 3]* [b = 2]	[R = 2]* [x = 1]* [b = 1]	[R = 2]* [x = 1]* [b = 2]
[R = 1]* [x = 1]* [b = 1]	.015	.005	.002	.001
[R = 1]* [x = 1]* [b = 2]	.043	.015	.005	.002
[R = 1]* [x = 2]* [b = 1]	.122	.043	.015	.005
[R = 1]* [x = 2]* [b = 2]	.349	.122	.043	.015
[R = 1]* [x = 3]* [b = 1]	1.000	.349	.122	.043
[R = 1]* [x = 3]* [b = 2]	.349	1.000	.349	.122
[R = 2]* [x = 1]* [b = 1]	.122	.349	1.000	.349
[R = 2]* [x = 1]* [b = 2]	.043	.122	.349	1.000
[R = 2]* [x = 2]* [b = 1]	.015	.043	.122	.349
[R = 2]* [x = 2]* [b = 2]	.005	.015	.043	.122
[R = 2]* [x = 3]* [b = 1]	.002	.005	.015	.043
[R = 2]* [x = 3]* [b = 2]	.001	.002	.005	.015
[R = 3]* [x = 1]* [b = 1]	.000	.001	.002	.005
[R = 3]* [x = 1]* [b = 2]	.000	.000	.001	.002
[R = 3]* [x = 2]* [b = 1]	.000	.000	.000	.001
[R = 3]* [x = 2]* [b = 2]	.000	.000	.000	.000
[R = 3]* [x = 3]* [b = 1]	.000	.000	.000	.000
[R = 3]* [x = 3]* [b = 2]	.000	.000	.000	.000

Working Correlation Matrix[a]

Measurement	Measurement [R = 2]* [x = 2]* [b = 1]	[R = 2]* [x = 2]* [b = 2]	[R = 2]* [x = 3]* [b = 1]	[R = 2]* [x = 3]* [b = 2]
[R = 1]* [x = 1]* [b = 1]	.000	.000	.000	.000
[R = 1]* [x = 1]* [b = 2]	.001	.000	.000	.000
[R = 1]* [x = 2]* [b = 1]	.002	.001	.000	.000
[R = 1]* [x = 2]* [b = 2]	.005	.002	.001	.000
[R = 1]* [x = 3]* [b = 1]	.015	.005	.002	.001
[R = 1]* [x = 3]* [b = 2]	.043	.015	.005	.002
[R = 2]* [x = 1]* [b = 1]	.122	.043	.015	.005
[R = 2]* [x = 1]* [b = 2]	.349	.122	.043	.015
[R = 2]* [x = 2]* [b = 1]	1.000	.349	.122	.043
[R = 2]* [x = 2]* [b = 2]	.349	1.000	.349	.122
[R = 2]* [x = 3]* [b = 1]	.122	.349	1.000	.349
[R = 2]* [x = 3]* [b = 2]	.043	.122	.349	1.000
[R = 3]* [x = 1]* [b = 1]	.015	.043	.122	.349
[R = 3]* [x = 1]* [b = 2]	.005	.015	.043	.122
[R = 3]* [x = 2]* [b = 1]	.002	.005	.015	.043
[R = 3]* [x = 2]* [b = 2]	.001	.002	.005	.015
[R = 3]* [x = 3]* [b = 1]	.000	.001	.002	.005
[R = 3]* [x = 3]* [b = 2]	.000	.000	.001	.002

Working Correlation Matrix[a]

Measurement	Measurement			
	[R = 3]* [x = 1]* [b = 1]	[R = 3]* [x = 1]* [b = 2]	[R = 3]* [x = 2]* [b = 1]	[R = 3]* [x = 2]* [b = 2]
[R = 1]* [x = 1]* [b = 1]	.000	.000	.000	.000
[R = 1]* [x = 1]* [b = 2]	.000	.000	.000	.000
[R = 1]* [x = 2]* [b = 1]	.000	.000	.000	.000
[R = 1]* [x = 2]* [b = 2]	.000	.000	.000	.000
[R = 1]* [x = 3]* [b = 1]	.000	.000	.000	.000
[R = 1]* [x = 3]* [b = 2]	.001	.000	.000	.000
[R = 2]* [x = 1]* [b = 1]	.002	.001	.000	.000
[R = 2]* [x = 1]* [b = 2]	.005	.002	.001	.000
[R = 2]* [x = 2]* [b = 1]	.015	.005	.002	.001
[R = 2]* [x = 2]* [b = 2]	.043	.015	.005	.002
[R = 2]* [x = 3]* [b = 1]	.122	.043	.015	.005
[R = 2]* [x = 3]* [b = 2]	.349	.122	.043	.015
[R = 3]* [x = 1]* [b = 1]	1.000	.349	.122	.043
[R = 3]* [x = 1]* [b = 2]	.349	1.000	.349	.122
[R = 3]* [x = 2]* [b = 1]	.122	.349	1.000	.349
[R = 3]* [x = 2]* [b = 2]	.043	.122	.349	1.000
[R = 3]* [x = 3]* [b = 1]	.015	.043	.122	.349
[R = 3]* [x = 3]* [b = 2]	.005	.015	.043	.122

Working Correlation Matrix[a]

Measurement	Measurement [R = 3]* [x = 3]* [b = 1]	[R = 3]* [x = 3]* [b = 2]
[R = 1]* [x = 1]* [b = 1]	.000	.000
[R = 1]* [x = 1]* [b = 2]	.000	.000
[R = 1]* [x = 2]* [b = 1]	.000	.000
[R = 1]* [x = 2]* [b = 2]	.000	.000
[R = 1]* [x = 3]* [b = 1]	.000	.000
[R = 1]* [x = 3]* [b = 2]	.000	.000
[R = 2]* [x = 1]* [b = 1]	.000	.000
[R = 2]* [x = 1]* [b = 2]	.000	.000
[R = 2]* [x = 2]* [b = 1]	.000	.000
[R = 2]* [x = 2]* [b = 2]	.001	.000
[R = 2]* [x = 3]* [b = 1]	.002	.001
[R = 2]* [x = 3]* [b = 2]	.005	.002
[R = 3]* [x = 1]* [b = 1]	.015	.005
[R = 3]* [x = 1]* [b = 2]	.043	.015
[R = 3]* [x = 2]* [b = 1]	.122	.043
[R = 3]* [x = 2]* [b = 2]	.349	.122
[R = 3]* [x = 3]* [b = 1]	1.000	.349
[R = 3]* [x = 3]* [b = 2]	.349	1.000

Dependent Variable: y
Model: (Intercept), R, x, b, gender_g, income_g, age, R * x, R * b, x * b, R * x * b

a. The AR(1) working correlation matrix structure is computed assuming the measurements are equally spaced for all subjects.

Estimated Marginal Means 1: R

Estimates

R	Mean	Std. Error	95% Wald Confidence Interval Lower	Upper
kin	.61	.035	.54	.68
acquaintance	.35	.033	.29	.42
general other	.18	.023	.14	.23

Covariates appearing in the model are fixed at the following values: age=37.04

Pairwise Comparisons

(I) R	(J) R	Mean Difference (I-J)	Std. Error	df	Bonferroni Sig.
kin	acquaintance	.26[a]	.023	1	.000
	general other	.43[a]	.029	1	.000
acquaintance	kin	-.26[a]	.023	1	.000
	general other	.17[a]	.019	1	.000
general other	kin	-.43[a]	.029	1	.000
	acquaintance	-.17[a]	.019	1	.000

Pairwise Comparisons

(I) R	(J) R	95% Wald Confidence Interval for Difference	
		Lower	Upper
kin	acquaintance	.21	.32
	general other	.37	.50
acquaintance	kin	-.32	-.21
	general other	.13	.22
general other	kin	-.50	-.37
	acquaintance	-.22	-.13

Pairwise comparisons of estimated marginal means based on the original scale of dependent variable y

a. The mean difference is significant at the .05 level.

Overall Test Results

Wald Chi-Square	df	Sig.
228.196	2	.000

The Wald chi-square tests the effect of R. This test is based on the linearly independent pairwise comparisons among the estimated marginal means.

Estimated Marginal Means 2: x

Estimates

x	Mean	Std. Error	95% Wald Confidence Interval Lower	Upper
2000	.67	.033	.60	.73
20,000	.37	.034	.31	.44
200,000	.13	.019	.10	.18

Covariates appearing in the model are fixed at the following values: age=37.04

Pairwise Comparisons

(I) x	(J) x	Mean Difference (I-J)	Std. Error	df	Bonferroni Sig.	95% Wald Confidence ... Lower
2000	20,000	.30[a]	.022	1	.000	.25
	200,000	.53[a]	.029	1	.000	.46
20,000	2000	-.30[a]	.022	1	.000	-.35
	200,000	.24[a]	.024	1	.000	.18
200,000	2000	-.53[a]	.029	1	.000	-.60
	20,000	-.24[a]	.024	1	.000	-.29

Pairwise Comparisons

(I) x	(J) x	95% Wald Confidence ... Upper
2000	20,000	.35
	200,000	.60
20,000	2000	-.25
	200,000	.29
200,000	2000	-.46
	20,000	-.18

Pairwise comparisons of estimated marginal means based on the original scale of dependent variable y

a. The mean difference is significant at the .05 level.

Overall Test Results

Wald Chi-Square	df	Sig.
356.083	2	.000

The Wald chi-square tests the effect of x. This test is based on the linearly independent pairwise comparisons among the estimated marginal means.

Estimated Marginal Means 3: b

Estimates

b	Mean	Std. Error	95% Wald Confidence Interval	
			Lower	Upper
H	.59	.035	.52	.65
L	.18	.023	.14	.23

Covariates appearing in the model are fixed at the following values: age=37.04

Pairwise Comparisons

(I) b	(J) b	Mean Difference (I-J)	Std. Error	df	Bonferroni Sig.	95% Wald Confidence ...
						Lower
H	L	.41[a]	.025	1	.000	.36
L	H	-.41[a]	.025	1	.000	-.45

Pairwise Comparisons

(I) b	(J) b	95% Wald Confidence ...
		Upper
H	L	.45
L	H	-.36

Pairwise comparisons of estimated marginal means based on the original scale of dependent variable y

a. The mean difference is significant at the .05 level.

Overall Test Results

Wald Chi-Square	df	Sig.
256.157	1	.000

The Wald chi-square tests the effect of b. This test is based on the linearly independent pairwise comparisons among the estimated marginal means.

Estimated Marginal Means 4: R* x

Estimates

R	x	Mean	Std. Error	95% Wald Confidence Interval	
				Lower	Upper
kin	2000	.83	.026	.78	.88
	20,000	.63	.037	.56	.70
	200,000	.31	.034	.25	.38
acquaintance	2000	.71	.034	.63	.77
	20,000	.36	.037	.29	.44
	200,000	.10	.018	.07	.14
general other	2000	.40	.037	.33	.48
	20,000	.17	.025	.13	.22
	200,000	.07	.014	.04	.10

Covariates appearing in the model are fixed at the following values: age=37.04

Pairwise Comparisons

(I) R*x	(J) R*x	Mean Difference (I-J)	Std. Error	df	Bonferroni Sig.	95% Wald Confidence ... Lower
[R=1]*[x=1]	[R=1]*[x=2]	.20[a]	.024	1	.000	.12
	[R=1]*[x=3]	.52[a]	.031	1	.000	.42
	[R=2]*[x=1]	.13[a]	.021	1	.000	.06
	[R=2]*[x=2]	.47[a]	.031	1	.000	.37
	[R=2]*[x=3]	.73[a]	.027	1	.000	.64
	[R=3]*[x=1]	.43[a]	.031	1	.000	.33
	[R=3]*[x=2]	.66[a]	.028	1	.000	.58
	[R=3]*[x=3]	.77[a]	.027	1	.000	.68
[R=1]*[x=2]	[R=1]*[x=1]	-.20[a]	.024	1	.000	-.28
	[R=1]*[x=3]	.32[a]	.028	1	.000	.23
	[R=2]*[x=1]	-.07	.025	1	.141	-.15
	[R=2]*[x=2]	.27[a]	.029	1	.000	.18
	[R=2]*[x=3]	.53[a]	.035	1	.000	.42
	[R=3]*[x=1]	.23[a]	.035	1	.000	.12
	[R=3]*[x=2]	.47[a]	.032	1	.000	.36
	[R=3]*[x=3]	.57[a]	.035	1	.000	.45
[R=1]*[x=3]	[R=1]*[x=1]	-.52[a]	.031	1	.000	-.62
	[R=1]*[x=2]	-.32[a]	.028	1	.000	-.41
	[R=2]*[x=1]	-.40[a]	.032	1	.000	-.50
	[R=2]*[x=2]	-.05	.029	1	1.000	-.15
	[R=2]*[x=3]	.21[a]	.028	1	.000	.12
	[R=3]*[x=1]	-.09	.035	1	.267	-.21
	[R=3]*[x=2]	.14[a]	.028	1	.000	.05
	[R=3]*[x=3]	.24[a]	.030	1	.000	.14
[R=2]*[x=1]	[R=1]*[x=1]	-.13[a]	.021	1	.000	-.20
	[R=1]*[x=2]	.07	.025	1	.141	-.01
	[R=1]*[x=3]	.40[a]	.032	1	.000	.29
	[R=2]*[x=2]	.34[a]	.030	1	.000	.25
	[R=2]*[x=3]	.60[a]	.033	1	.000	.50
	[R=3]*[x=1]	.30[a]	.030	1	.000	.21
	[R=3]*[x=2]	.54[a]	.031	1	.000	.44
	[R=3]*[x=3]	.64[a]	.033	1	.000	.53
[R=2]*[x=2]	[R=1]*[x=1]	-.47[a]	.031	1	.000	-.57
	[R=1]*[x=2]	-.27[a]	.029	1	.000	-.36

Pairwise Comparisons

(I) R*x	(J) R*x	95% Wald Confidence ... Upper
[R=1]*[x=1]	[R=1]*[x=2]	.28
	[R=1]*[x=3]	.62
	[R=2]*[x=1]	.20
	[R=2]*[x=2]	.57
	[R=2]*[x=3]	.82
	[R=3]*[x=1]	.53
	[R=3]*[x=2]	.75
	[R=3]*[x=3]	.85
[R=1]*[x=2]	[R=1]*[x=1]	-.12
	[R=1]*[x=3]	.41
	[R=2]*[x=1]	.01
	[R=2]*[x=2]	.36
	[R=2]*[x=3]	.64
	[R=3]*[x=1]	.34
	[R=3]*[x=2]	.57
	[R=3]*[x=3]	.68
[R=1]*[x=3]	[R=1]*[x=1]	-.42
	[R=1]*[x=2]	-.23
	[R=2]*[x=1]	-.29
	[R=2]*[x=2]	.04
	[R=2]*[x=3]	.30
	[R=3]*[x=1]	.02
	[R=3]*[x=2]	.23
	[R=3]*[x=3]	.34
[R=2]*[x=1]	[R=1]*[x=1]	-.06
	[R=1]*[x=2]	.15
	[R=1]*[x=3]	.50
	[R=2]*[x=2]	.44
	[R=2]*[x=3]	.71
	[R=3]*[x=1]	.40
	[R=3]*[x=2]	.63
	[R=3]*[x=3]	.74
[R=2]*[x=2]	[R=1]*[x=1]	-.37
	[R=1]*[x=2]	-.18

Pairwise Comparisons

(I) R*x	(J) R*x	Mean Difference (I-J)	Std. Error	df	Bonferroni Sig.	95% Wald Confidence ... Lower
	[R=1]*[x=3]	.05	.029	1	1.000	-.04
	[R=2]*[x=1]	-.34[a]	.030	1	.000	-.44
	[R=2]*[x=3]	.26[a]	.029	1	.000	.17
	[R=3]*[x=1]	-.04	.028	1	1.000	-.13
	[R=3]*[x=2]	.19[a]	.022	1	.000	.13
	[R=3]*[x=3]	.30[a]	.031	1	.000	.20
[R=2]*[x=3]	[R=1]*[x=1]	-.73[a]	.027	1	.000	-.82
	[R=1]*[x=2]	-.53[a]	.035	1	.000	-.64
	[R=1]*[x=3]	-.21[a]	.028	1	.000	-.30
	[R=2]*[x=1]	-.60[a]	.033	1	.000	-.71
	[R=2]*[x=2]	-.26[a]	.029	1	.000	-.35
	[R=3]*[x=1]	-.30[a]	.032	1	.000	-.40
	[R=3]*[x=2]	-.07[a]	.019	1	.016	-.13
	[R=3]*[x=3]	.04[a]	.011	1	.048	.00
[R=3]*[x=1]	[R=1]*[x=1]	-.43[a]	.031	1	.000	-.53
	[R=1]*[x=2]	-.23[a]	.035	1	.000	-.34
	[R=1]*[x=3]	.09	.035	1	.267	-.02
	[R=2]*[x=1]	-.30[a]	.030	1	.000	-.40
	[R=2]*[x=2]	.04	.028	1	1.000	-.05
	[R=2]*[x=3]	.30[a]	.032	1	.000	.20
	[R=3]*[x=2]	.23[a]	.025	1	.000	.15
	[R=3]*[x=3]	.34[a]	.033	1	.000	.23
[R=3]*[x=2]	[R=1]*[x=1]	-.66[a]	.028	1	.000	-.75
	[R=1]*[x=2]	-.47[a]	.032	1	.000	-.57
	[R=1]*[x=3]	-.14[a]	.028	1	.000	-.23
	[R=2]*[x=1]	-.54[a]	.031	1	.000	-.63
	[R=2]*[x=2]	-.19[a]	.022	1	.000	-.26
	[R=2]*[x=3]	.07[a]	.019	1	.016	.01
	[R=3]*[x=1]	-.23[a]	.025	1	.000	-.31
	[R=3]*[x=3]	.10[a]	.018	1	.000	.04
[R=3]*[x=3]	[R=1]*[x=1]	-.77[a]	.027	1	.000	-.85
	[R=1]*[x=2]	-.57[a]	.035	1	.000	-.68
	[R=1]*[x=3]	-.24[a]	.030	1	.000	-.34
	[R=2]*[x=1]	-.64[a]	.033	1	.000	-.74

Pairwise Comparisons

(I) R*x	(J) R*x	95% Wald Confidence ... Upper
	[R=1]*[x=3]	.15
	[R=2]*[x=1]	-.25
	[R=2]*[x=3]	.35
	[R=3]*[x=1]	.05
	[R=3]*[x=2]	.26
	[R=3]*[x=3]	.39
[R=2]*[x=3]	[R=1]*[x=1]	-.64
	[R=1]*[x=2]	-.42
	[R=1]*[x=3]	-.12
	[R=2]*[x=1]	-.50
	[R=2]*[x=2]	-.17
	[R=3]*[x=1]	-.20
	[R=3]*[x=2]	-.01
	[R=3]*[x=3]	.07
[R=3]*[x=1]	[R=1]*[x=1]	-.33
	[R=1]*[x=2]	-.12
	[R=1]*[x=3]	.21
	[R=2]*[x=1]	-.21
	[R=2]*[x=2]	.13
	[R=2]*[x=3]	.40
	[R=3]*[x=2]	.31
	[R=3]*[x=3]	.44
[R=3]*[x=2]	[R=1]*[x=1]	-.58
	[R=1]*[x=2]	-.36
	[R=1]*[x=3]	-.05
	[R=2]*[x=1]	-.44
	[R=2]*[x=2]	-.13
	[R=2]*[x=3]	.13
	[R=3]*[x=1]	-.15
	[R=3]*[x=3]	.16
[R=3]*[x=3]	[R=1]*[x=1]	-.68
	[R=1]*[x=2]	-.45
	[R=1]*[x=3]	-.14
	[R=2]*[x=1]	-.53

Pairwise Comparisons

(I) R*x	(J) R*x	Mean Difference (I-J)	Std. Error	df	Bonferroni Sig.	95% Wald Confidence ... Lower
	[R=2]*[x=2]	-.30[a]	.031	1	.000	-.39
	[R=2]*[x=3]	-.04[a]	.011	1	.048	-.07
	[R=3]*[x=1]	-.34[a]	.033	1	.000	-.44
	[R=3]*[x=2]	-.10[a]	.018	1	.000	-.16

Pairwise Comparisons

(I) R*x	(J) R*x	95% Wald Confidence ... Upper
	[R=2]*[x=2]	-.20
	[R=2]*[x=3]	.00
	[R=3]*[x=1]	-.23
	[R=3]*[x=2]	-.04

Pairwise comparisons of estimated marginal means based on the original scale of dependent variable y

a. The mean difference is significant at the .05 level.

Overall Test Results

Wald Chi-Square	df	Sig.
861.688	8	.000

The Wald chi-square tests the effect of R*x. This test is based on the linearly independent pairwise comparisons among the estimated marginal means.

Estimated Marginal Means 5: R* b

Estimates

R	b	Mean	Std. Error	95% Wald Confidence Interval	
				Lower	Upper
kin	H	.82	.027	.76	.87
	L	.35	.034	.29	.42
acquaintance	H	.62	.036	.55	.69
	L	.15	.022	.11	.20
general other	H	.28	.031	.23	.35
	L	.10	.018	.07	.15

Covariates appearing in the model are fixed at the following values: age=37.04

Pairwise Comparisons

(I) R*b	(J) R*b	Mean Difference (I-J)	Std. Error	df	Bonferroni Sig.	95% Wald Confidence ...
						Lower
[R=1]*[b=1]	[R=1]*[b=2]	.47[a]	.029	1	.000	.38
	[R=2]*[b=1]	.20[a]	.023	1	.000	.13
	[R=2]*[b=2]	.67[a]	.028	1	.000	.59
	[R=3]*[b=1]	.54[a]	.030	1	.000	.45
	[R=3]*[b=2]	.71[a]	.027	1	.000	.63
[R=1]*[b=2]	[R=1]*[b=1]	-.47[a]	.029	1	.000	-.55
	[R=2]*[b=1]	-.27[a]	.030	1	.000	-.36
	[R=2]*[b=2]	.20[a]	.026	1	.000	.13
	[R=3]*[b=1]	.07	.031	1	.363	-.02
	[R=3]*[b=2]	.25[a]	.029	1	.000	.16
[R=2]*[b=1]	[R=1]*[b=1]	-.20[a]	.023	1	.000	-.26
	[R=1]*[b=2]	.27[a]	.030	1	.000	.18
	[R=2]*[b=2]	.47[a]	.031	1	.000	.38
	[R=3]*[b=1]	.34[a]	.027	1	.000	.26
	[R=3]*[b=2]	.52[a]	.032	1	.000	.42
[R=2]*[b=2]	[R=1]*[b=1]	-.67[a]	.028	1	.000	-.75
	[R=1]*[b=2]	-.20[a]	.026	1	.000	-.28
	[R=2]*[b=1]	-.47[a]	.031	1	.000	-.56
	[R=3]*[b=1]	-.13[a]	.021	1	.000	-.20
	[R=3]*[b=2]	.05[a]	.014	1	.015	.00
[R=3]*[b=1]	[R=1]*[b=1]	-.54[a]	.030	1	.000	-.62
	[R=1]*[b=2]	-.07	.031	1	.363	-.16
	[R=2]*[b=1]	-.34[a]	.027	1	.000	-.42

Pairwise Comparisons

(I) R*b	(J) R*b	95% Wald Confidence ... Upper
[R=1]*[b=1]	[R=1]*[b=2]	.55
	[R=2]*[b=1]	.26
	[R=2]*[b=2]	.75
	[R=3]*[b=1]	.62
	[R=3]*[b=2]	.79
[R=1]*[b=2]	[R=1]*[b=1]	-.38
	[R=2]*[b=1]	-.18
	[R=2]*[b=2]	.28
	[R=3]*[b=1]	.16
	[R=3]*[b=2]	.33
[R=2]*[b=1]	[R=1]*[b=1]	-.13
	[R=1]*[b=2]	.36
	[R=2]*[b=2]	.56
	[R=3]*[b=1]	.42
	[R=3]*[b=2]	.61
[R=2]*[b=2]	[R=1]*[b=1]	-.59
	[R=1]*[b=2]	-.13
	[R=2]*[b=1]	-.38
	[R=3]*[b=1]	-.07
	[R=3]*[b=2]	.09
[R=3]*[b=1]	[R=1]*[b=1]	-.45
	[R=1]*[b=2]	.02
	[R=2]*[b=1]	-.26

Pairwise Comparisons

(I) R*b	(J) R*b	Mean Difference (I-J)	Std. Error	df	Bonferroni Sig.	95% Wald Confidence ... Lower
	[R=2]*[b=2]	.13[a]	.021	1	.000	.07
	[R=3]*[b=2]	.18[a]	.023	1	.000	.11
[R=3]*[b=2]	[R=1]*[b=1]	-.71[a]	.027	1	.000	-.79
	[R=1]*[b=2]	-.25[a]	.029	1	.000	-.33
	[R=2]*[b=1]	-.52[a]	.032	1	.000	-.61
	[R=2]*[b=2]	-.05[a]	.014	1	.015	-.09
	[R=3]*[b=1]	-.18[a]	.023	1	.000	-.25

Pairwise Comparisons

(I) R*b	(J) R*b	95% Wald Confidence ... Upper
	[R=2]*[b=2]	.20
	[R=3]*[b=2]	.25
[R=3]*[b=2]	[R=1]*[b=1]	-.63
	[R=1]*[b=2]	-.16
	[R=2]*[b=1]	-.42
	[R=2]*[b=2]	.00
	[R=3]*[b=1]	-.11

Pairwise comparisons of estimated marginal means based on the original scale of dependent variable y

a. The mean difference is significant at the .05 level.

Overall Test Results

Wald Chi-Square	df	Sig.
725.280	5	.000

The Wald chi-square tests the effect of R*b. This test is based on the linearly independent pairwise comparisons among the estimated marginal means.

Estimated Marginal Means 6: x* b

Estimates

x	b	Mean	Std. Error	95% Wald Confidence Interval Lower	Upper
2000	H	.85	.024	.80	.89
	L	.41	.034	.34	.48
20,000	H	.65	.035	.58	.71
	L	.16	.023	.12	.21
200,000	H	.22	.027	.17	.27
	L	.08	.015	.06	.11

Covariates appearing in the model are fixed at the following values: age=37.04

Pairwise Comparisons

(I) x*b	(J) x*b	Mean Difference (I-J)	Std. Error	df	Bonferroni Sig.	95% Wald Confidence ... Lower
[x=1]*[b=1]	[x=1]*[b=2]	.44[a]	.028	1	.000	.36
	[x=2]*[b=1]	.21[a]	.021	1	.000	.14
	[x=2]*[b=2]	.70[a]	.026	1	.000	.62
	[x=3]*[b=1]	.64[a]	.027	1	.000	.56
	[x=3]*[b=2]	.77[a]	.025	1	.000	.70
[x=1]*[b=2]	[x=1]*[b=1]	-.44[a]	.028	1	.000	-.53
	[x=2]*[b=1]	-.24[a]	.028	1	.000	-.32
	[x=2]*[b=2]	.25[a]	.024	1	.000	.18
	[x=3]*[b=1]	.19[a]	.029	1	.000	.11
	[x=3]*[b=2]	.33[a]	.030	1	.000	.24
[x=2]*[b=1]	[x=1]*[b=1]	-.21[a]	.021	1	.000	-.27
	[x=1]*[b=2]	.24[a]	.028	1	.000	.16
	[x=2]*[b=2]	.49[a]	.029	1	.000	.40
	[x=3]*[b=1]	.43[a]	.027	1	.000	.35
	[x=3]*[b=2]	.57[a]	.032	1	.000	.47
[x=2]*[b=2]	[x=1]*[b=1]	-.70[a]	.026	1	.000	-.77
	[x=1]*[b=2]	-.25[a]	.024	1	.000	-.32
	[x=2]*[b=1]	-.49[a]	.029	1	.000	-.57
	[x=3]*[b=1]	-.06	.020	1	.061	-.12
	[x=3]*[b=2]	.08[a]	.016	1	.000	.03
[x=3]*[b=1]	[x=1]*[b=1]	-.64[a]	.027	1	.000	-.72
	[x=1]*[b=2]	-.19[a]	.029	1	.000	-.28
	[x=2]*[b=1]	-.43[a]	.027	1	.000	-.51
	[x=2]*[b=2]	.06	.020	1	.061	.00
	[x=3]*[b=2]	.14[a]	.019	1	.000	.08
[x=3]*[b=2]	[x=1]*[b=1]	-.77[a]	.025	1	.000	-.85
	[x=1]*[b=2]	-.33[a]	.030	1	.000	-.42
	[x=2]*[b=1]	-.57[a]	.032	1	.000	-.66
	[x=2]*[b=2]	-.08[a]	.016	1	.000	-.13
	[x=3]*[b=1]	-.14[a]	.019	1	.000	-.19

Pairwise Comparisons

(I) x*b	(J) x*b	95% Wald Confidence ... Upper
[x=1]*[b=1]	[x=1]*[b=2]	.53
	[x=2]*[b=1]	.27
	[x=2]*[b=2]	.77
	[x=3]*[b=1]	.72
	[x=3]*[b=2]	.85
[x=1]*[b=2]	[x=1]*[b=1]	-.36
	[x=2]*[b=1]	-.16
	[x=2]*[b=2]	.32
	[x=3]*[b=1]	.28
	[x=3]*[b=2]	.42
[x=2]*[b=1]	[x=1]*[b=1]	-.14
	[x=1]*[b=2]	.32
	[x=2]*[b=2]	.57
	[x=3]*[b=1]	.51
	[x=3]*[b=2]	.66
[x=2]*[b=2]	[x=1]*[b=1]	-.62
	[x=1]*[b=2]	-.18
	[x=2]*[b=1]	-.40
	[x=3]*[b=1]	.00
	[x=3]*[b=2]	.13
[x=3]*[b=1]	[x=1]*[b=1]	-.56
	[x=1]*[b=2]	-.11
	[x=2]*[b=1]	-.35
	[x=2]*[b=2]	.12
	[x=3]*[b=2]	.19
[x=3]*[b=2]	[x=1]*[b=1]	-.70
	[x=1]*[b=2]	-.24
	[x=2]*[b=1]	-.47
	[x=2]*[b=2]	-.03
	[x=3]*[b=1]	-.08

Pairwise comparisons of estimated marginal means based on the original scale of dependent variable y

a. The mean difference is significant at the .05 level.

Overall Test Results

Wald Chi-Square	df	Sig.
1062.839	5	.000

The Wald chi-square tests the effect of x*b. This test is based on the linearly independent pairwise comparisons among the estimated marginal means.

Estimated Marginal Means 7: R* x* b

Estimates

R	x	b	Mean	Std. Error	95% Wald Confidence Interval	
					Lower	Upper
kin	2000	H	.93	.017	.89	.96
		L	.65	.037	.58	.72
	20,000	H	.86	.025	.80	.90
		L	.33	.036	.26	.40
	200,000	H	.53	.042	.45	.61
		L	.15	.025	.11	.21
acquaintance	2000	H	.89	.022	.84	.92
		L	.42	.040	.34	.50
	20,000	H	.71	.035	.64	.78
		L	.12	.022	.08	.16
	200,000	H	.18	.028	.13	.24
		L	.06	.014	.03	.09
general other	2000	H	.65	.038	.57	.72
		L	.20	.029	.15	.26
	20,000	H	.29	.035	.22	.36
		L	.09	.019	.06	.14
	200,000	H	.08	.017	.05	.12
		L	.06	.015	.04	.10

Covariates appearing in the model are fixed at the following values: age=37.04

Pairwise Comparisons

(I) R*x*b	(J) R*x*b	Mean Difference (I-J)	Std. Error	df	Bonferroni Sig.
[R=1]*[x=1]*[b=1]	[R=1]*[x=1]*[b=2]	.28[a]	.032	1	.000
	[R=1]*[x=2]*[b=1]	.07[a]	.017	1	.004
	[R=1]*[x=2]*[b=2]	.60[a]	.034	1	.000
	[R=1]*[x=3]*[b=1]	.40[a]	.037	1	.000
	[R=1]*[x=3]*[b=2]	.78[a]	.027	1	.000
	[R=2]*[x=1]*[b=1]	.04	.014	1	.499
	[R=2]*[x=1]*[b=2]	.51[a]	.037	1	.000
	[R=2]*[x=2]*[b=1]	.22[a]	.030	1	.000
	[R=2]*[x=2]*[b=2]	.81[a]	.025	1	.000
	[R=2]*[x=3]*[b=1]	.75[a]	.028	1	.000
	[R=2]*[x=3]*[b=2]	.87[a]	.021	1	.000
	[R=3]*[x=1]*[b=1]	.28[a]	.033	1	.000
	[R=3]*[x=1]*[b=2]	.73[a]	.029	1	.000
	[R=3]*[x=2]*[b=1]	.64[a]	.034	1	.000
	[R=3]*[x=2]*[b=2]	.84[a]	.023	1	.000
	[R=3]*[x=3]*[b=1]	.85[a]	.022	1	.000
	[R=3]*[x=3]*[b=2]	.87[a]	.021	1	.000
[R=1]*[x=1]*[b=2]	[R=1]*[x=1]*[b=1]	-.28[a]	.032	1	.000
	[R=1]*[x=2]*[b=1]	-.21[a]	.032	1	.000
	[R=1]*[x=2]*[b=2]	.32[a]	.030	1	.000
	[R=1]*[x=3]*[b=1]	.12	.040	1	.417
	[R=1]*[x=3]*[b=2]	.50[a]	.035	1	.000
	[R=2]*[x=1]*[b=1]	-.24[a]	.032	1	.000
	[R=2]*[x=1]*[b=2]	.23[a]	.034	1	.000
	[R=2]*[x=2]*[b=1]	-.06	.032	1	1.000
	[R=2]*[x=2]*[b=2]	.54[a]	.035	1	.000
	[R=2]*[x=3]*[b=1]	.47[a]	.037	1	.000
	[R=2]*[x=3]*[b=2]	.60[a]	.037	1	.000
	[R=3]*[x=1]*[b=1]	.00	.036	1	1.000
	[R=3]*[x=1]*[b=2]	.45[a]	.035	1	.000
	[R=3]*[x=2]*[b=1]	.36[a]	.037	1	.000
	[R=3]*[x=2]*[b=2]	.56[a]	.035	1	.000
	[R=3]*[x=3]*[b=1]	.58[a]	.038	1	.000
	[R=3]*[x=3]*[b=2]	.59[a]	.037	1	.000

Pairwise Comparisons

(I) R*x*b	(J) R*x*b	95% Wald Confidence Interval for Difference	
		Lower	Upper
[R=1]*[x=1]*[b=1]	[R=1]*[x=1]*[b=2]	.16	.39
	[R=1]*[x=2]*[b=1]	.01	.13
	[R=1]*[x=2]*[b=2]	.48	.72
	[R=1]*[x=3]*[b=1]	.26	.53
	[R=1]*[x=3]*[b=2]	.68	.88
	[R=2]*[x=1]*[b=1]	-.01	.09
	[R=2]*[x=1]*[b=2]	.38	.65
	[R=2]*[x=2]*[b=1]	.11	.32
	[R=2]*[x=2]*[b=2]	.73	.90
	[R=2]*[x=3]*[b=1]	.65	.85
	[R=2]*[x=3]*[b=2]	.80	.95
	[R=3]*[x=1]*[b=1]	.16	.40
	[R=3]*[x=1]*[b=2]	.63	.84
	[R=3]*[x=2]*[b=1]	.52	.76
	[R=3]*[x=2]*[b=2]	.75	.92
	[R=3]*[x=3]*[b=1]	.77	.93
	[R=3]*[x=3]*[b=2]	.79	.95
[R=1]*[x=1]*[b=2]	[R=1]*[x=1]*[b=1]	-.39	-.16
	[R=1]*[x=2]*[b=1]	-.32	-.09
	[R=1]*[x=2]*[b=2]	.22	.43
	[R=1]*[x=3]*[b=1]	-.02	.26
	[R=1]*[x=3]*[b=2]	.38	.63
	[R=2]*[x=1]*[b=1]	-.35	-.12
	[R=2]*[x=1]*[b=2]	.11	.36
	[R=2]*[x=2]*[b=1]	-.18	.05
	[R=2]*[x=2]*[b=2]	.41	.66
	[R=2]*[x=3]*[b=1]	.34	.61
	[R=2]*[x=3]*[b=2]	.46	.73
	[R=3]*[x=1]*[b=1]	-.12	.13
	[R=3]*[x=1]*[b=2]	.33	.58
	[R=3]*[x=2]*[b=1]	.23	.50
	[R=3]*[x=2]*[b=2]	.43	.68
	[R=3]*[x=3]*[b=1]	.44	.71
	[R=3]*[x=3]*[b=2]	.46	.72

Pairwise Comparisons

(I) R*x*b	(J) R*x*b	Mean Difference (I-J)	Std. Error	df	Bonferroni Sig.
[R=1]*[x=2]*[b=1]	[R=1]*[x=1]*[b=1]	-.07[a]	.017	1	.004
	[R=1]*[x=1]*[b=2]	.21[a]	.032	1	.000
	[R=1]*[x=2]*[b=2]	.53[a]	.033	1	.000
	[R=1]*[x=3]*[b=1]	.33[a]	.034	1	.000
	[R=1]*[x=3]*[b=2]	.71[a]	.029	1	.000
	[R=2]*[x=1]*[b=1]	-.03	.017	1	1.000
	[R=2]*[x=1]*[b=2]	.44[a]	.037	1	.000
	[R=2]*[x=2]*[b=1]	.15[a]	.027	1	.000
	[R=2]*[x=2]*[b=2]	.74[a]	.028	1	.000
	[R=2]*[x=3]*[b=1]	.68[a]	.031	1	.000
	[R=2]*[x=3]*[b=2]	.80[a]	.027	1	.000
	[R=3]*[x=1]*[b=1]	.21[a]	.033	1	.000
	[R=3]*[x=1]*[b=2]	.66[a]	.033	1	.000
	[R=3]*[x=2]*[b=1]	.57[a]	.034	1	.000
	[R=3]*[x=2]*[b=2]	.77[a]	.027	1	.000
	[R=3]*[x=3]*[b=1]	.78[a]	.027	1	.000
	[R=3]*[x=3]*[b=2]	.80[a]	.027	1	.000
[R=1]*[x=2]*[b=2]	[R=1]*[x=1]*[b=1]	-.60[a]	.034	1	.000
	[R=1]*[x=1]*[b=2]	-.32[a]	.030	1	.000
	[R=1]*[x=2]*[b=1]	-.53[a]	.033	1	.000
	[R=1]*[x=3]*[b=1]	-.20[a]	.036	1	.000
	[R=1]*[x=3]*[b=2]	.18[a]	.028	1	.000
	[R=2]*[x=1]*[b=1]	-.56[a]	.034	1	.000
	[R=2]*[x=1]*[b=2]	-.09	.032	1	.875
	[R=2]*[x=2]*[b=1]	-.38[a]	.034	1	.000
	[R=2]*[x=2]*[b=2]	.21[a]	.030	1	.000
	[R=2]*[x=3]*[b=1]	.15[a]	.034	1	.002
	[R=2]*[x=3]*[b=2]	.27[a]	.035	1	.000
	[R=3]*[x=1]*[b=1]	-.32[a]	.041	1	.000
	[R=3]*[x=1]*[b=2]	.13[a]	.033	1	.010
	[R=3]*[x=2]*[b=1]	.04	.037	1	1.000
	[R=3]*[x=2]*[b=2]	.24[a]	.031	1	.000
	[R=3]*[x=3]*[b=1]	.25[a]	.034	1	.000
	[R=3]*[x=3]*[b=2]	.27[a]	.035	1	.000

Pairwise Comparisons

(I) R*x*b	(J) R*x*b	95% Wald Confidence Interval for Difference Lower	Upper
[R=1]*[x=2]*[b=1]	[R=1]*[x=1]*[b=1]	-.13	-.01
	[R=1]*[x=1]*[b=2]	.09	.32
	[R=1]*[x=2]*[b=2]	.41	.65
	[R=1]*[x=3]*[b=1]	.20	.45
	[R=1]*[x=3]*[b=2]	.60	.82
	[R=2]*[x=1]*[b=1]	-.09	.03
	[R=2]*[x=1]*[b=2]	.31	.58
	[R=2]*[x=2]*[b=1]	.05	.24
	[R=2]*[x=2]*[b=2]	.64	.85
	[R=2]*[x=3]*[b=1]	.57	.79
	[R=2]*[x=3]*[b=2]	.71	.90
	[R=3]*[x=1]*[b=1]	.09	.33
	[R=3]*[x=1]*[b=2]	.54	.78
	[R=3]*[x=2]*[b=1]	.45	.69
	[R=3]*[x=2]*[b=2]	.67	.86
	[R=3]*[x=3]*[b=1]	.69	.88
	[R=3]*[x=3]*[b=2]	.70	.90
[R=1]*[x=2]*[b=2]	[R=1]*[x=1]*[b=1]	-.72	-.48
	[R=1]*[x=1]*[b=2]	-.43	-.22
	[R=1]*[x=2]*[b=1]	-.65	-.41
	[R=1]*[x=3]*[b=1]	-.33	-.07
	[R=1]*[x=3]*[b=2]	.08	.28
	[R=2]*[x=1]*[b=1]	-.68	-.44
	[R=2]*[x=1]*[b=2]	-.20	.03
	[R=2]*[x=2]*[b=1]	-.51	-.26
	[R=2]*[x=2]*[b=2]	.11	.32
	[R=2]*[x=3]*[b=1]	.03	.27
	[R=2]*[x=3]*[b=2]	.15	.40
	[R=3]*[x=1]*[b=1]	-.47	-.17
	[R=3]*[x=1]*[b=2]	.01	.25
	[R=3]*[x=2]*[b=1]	-.09	.17
	[R=3]*[x=2]*[b=2]	.12	.35
	[R=3]*[x=3]*[b=1]	.13	.38
	[R=3]*[x=3]*[b=2]	.14	.39

Pairwise Comparisons

(I) R*x*b	(J) R*x*b	Mean Difference (I-J)	Std. Error	df	Bonferroni Sig.
[R=1]*[x=3]*[b=1]	[R=1]*[x=1]*[b=1]	-.40[a]	.037	1	.000
	[R=1]*[x=1]*[b=2]	-.12	.040	1	.417
	[R=1]*[x=2]*[b=1]	-.33[a]	.034	1	.000
	[R=1]*[x=2]*[b=2]	.20[a]	.036	1	.000
	[R=1]*[x=3]*[b=2]	.38[a]	.036	1	.000
	[R=2]*[x=1]*[b=1]	-.36[a]	.037	1	.000
	[R=2]*[x=1]*[b=2]	.12	.041	1	.699
	[R=2]*[x=2]*[b=1]	-.18[a]	.035	1	.000
	[R=2]*[x=2]*[b=2]	.42[a]	.038	1	.000
	[R=2]*[x=3]*[b=1]	.35[a]	.035	1	.000
	[R=2]*[x=3]*[b=2]	.48[a]	.041	1	.000
	[R=3]*[x=1]*[b=1]	-.12	.041	1	.661
	[R=3]*[x=1]*[b=2]	.34[a]	.039	1	.000
	[R=3]*[x=2]*[b=1]	.25[a]	.038	1	.000
	[R=3]*[x=2]*[b=2]	.44[a]	.038	1	.000
	[R=3]*[x=3]*[b=1]	.46[a]	.040	1	.000
	[R=3]*[x=3]*[b=2]	.47[a]	.040	1	.000
[R=1]*[x=3]*[b=2]	[R=1]*[x=1]*[b=1]	-.78[a]	.027	1	.000
	[R=1]*[x=1]*[b=2]	-.50[a]	.035	1	.000
	[R=1]*[x=2]*[b=1]	-.71[a]	.029	1	.000
	[R=1]*[x=2]*[b=2]	-.18[a]	.028	1	.000
	[R=1]*[x=3]*[b=1]	-.38[a]	.036	1	.000
	[R=2]*[x=1]*[b=1]	-.74[a]	.029	1	.000
	[R=2]*[x=1]*[b=2]	-.27[a]	.035	1	.000
	[R=2]*[x=2]*[b=1]	-.56[a]	.034	1	.000
	[R=2]*[x=2]*[b=2]	.03	.023	1	1.000
	[R=2]*[x=3]*[b=1]	-.03	.026	1	1.000
	[R=2]*[x=3]*[b=2]	.09[a]	.023	1	.006
	[R=3]*[x=1]*[b=1]	-.50[a]	.039	1	.000
	[R=3]*[x=1]*[b=2]	-.05	.030	1	1.000
	[R=3]*[x=2]*[b=1]	-.14[a]	.035	1	.010
	[R=3]*[x=2]*[b=2]	.06	.024	1	1.000
	[R=3]*[x=3]*[b=1]	.07	.023	1	.281
	[R=3]*[x=3]*[b=2]	.09[a]	.024	1	.026

Pairwise Comparisons

(I) R*x*b	(J) R*x*b	95% Wald Confidence Interval for Difference Lower	Upper
[R=1]*[x=3]*[b=1]	[R=1]*[x=1]*[b=1]	-.53	-.26
	[R=1]*[x=1]*[b=2]	-.26	.02
	[R=1]*[x=2]*[b=1]	-.45	-.20
	[R=1]*[x=2]*[b=2]	.07	.33
	[R=1]*[x=3]*[b=2]	.25	.51
	[R=2]*[x=1]*[b=1]	-.49	-.22
	[R=2]*[x=1]*[b=2]	-.03	.26
	[R=2]*[x=2]*[b=1]	-.31	-.05
	[R=2]*[x=2]*[b=2]	.28	.55
	[R=2]*[x=3]*[b=1]	.23	.48
	[R=2]*[x=3]*[b=2]	.33	.62
	[R=3]*[x=1]*[b=1]	-.26	.03
	[R=3]*[x=1]*[b=2]	.19	.48
	[R=3]*[x=2]*[b=1]	.11	.38
	[R=3]*[x=2]*[b=2]	.30	.58
	[R=3]*[x=3]*[b=1]	.31	.60
	[R=3]*[x=3]*[b=2]	.33	.62
[R=1]*[x=3]*[b=2]	[R=1]*[x=1]*[b=1]	-.88	-.68
	[R=1]*[x=1]*[b=2]	-.63	-.38
	[R=1]*[x=2]*[b=1]	-.82	-.60
	[R=1]*[x=2]*[b=2]	-.28	-.08
	[R=1]*[x=3]*[b=1]	-.51	-.25
	[R=2]*[x=1]*[b=1]	-.84	-.64
	[R=2]*[x=1]*[b=2]	-.39	-.14
	[R=2]*[x=2]*[b=1]	-.69	-.44
	[R=2]*[x=2]*[b=2]	-.05	.12
	[R=2]*[x=3]*[b=1]	-.13	.06
	[R=2]*[x=3]*[b=2]	.01	.17
	[R=3]*[x=1]*[b=1]	-.64	-.36
	[R=3]*[x=1]*[b=2]	-.16	.06
	[R=3]*[x=2]*[b=1]	-.26	-.01
	[R=3]*[x=2]*[b=2]	-.03	.14
	[R=3]*[x=3]*[b=1]	-.01	.16
	[R=3]*[x=3]*[b=2]	.00	.17

Pairwise Comparisons

(I) R*x*b	(J) R*x*b	Mean Difference (I-J)	Std. Error	df	Bonferroni Sig.
[R=2]*[x=1]*[b=1]	[R=1]*[x=1]*[b=1]	-.04	.014	1	.499
	[R=1]*[x=1]*[b=2]	.24[a]	.032	1	.000
	[R=1]*[x=2]*[b=1]	.03	.017	1	1.000
	[R=1]*[x=2]*[b=2]	.56[a]	.034	1	.000
	[R=1]*[x=3]*[b=1]	.36[a]	.037	1	.000
	[R=1]*[x=3]*[b=2]	.74[a]	.029	1	.000
	[R=2]*[x=1]*[b=2]	.47[a]	.036	1	.000
	[R=2]*[x=2]*[b=1]	.18[a]	.027	1	.000
	[R=2]*[x=2]*[b=2]	.77[a]	.027	1	.000
	[R=2]*[x=3]*[b=1]	.71[a]	.029	1	.000
	[R=2]*[x=3]*[b=2]	.83[a]	.025	1	.000
	[R=3]*[x=1]*[b=1]	.24[a]	.031	1	.000
	[R=3]*[x=1]*[b=2]	.69[a]	.030	1	.000
	[R=3]*[x=2]*[b=1]	.60[a]	.033	1	.000
	[R=3]*[x=2]*[b=2]	.80[a]	.025	1	.000
	[R=3]*[x=3]*[b=1]	.81[a]	.025	1	.000
	[R=3]*[x=3]*[b=2]	.83[a]	.024	1	.000
[R=2]*[x=1]*[b=2]	[R=1]*[x=1]*[b=1]	-.51[a]	.037	1	.000
	[R=1]*[x=1]*[b=2]	-.23[a]	.034	1	.000
	[R=1]*[x=2]*[b=1]	-.44[a]	.037	1	.000
	[R=1]*[x=2]*[b=2]	.09	.032	1	.875
	[R=1]*[x=3]*[b=1]	-.12	.041	1	.699
	[R=1]*[x=3]*[b=2]	.27[a]	.035	1	.000
	[R=2]*[x=1]*[b=1]	-.47[a]	.036	1	.000
	[R=2]*[x=2]*[b=1]	-.30[a]	.034	1	.000
	[R=2]*[x=2]*[b=2]	.30[a]	.034	1	.000
	[R=2]*[x=3]*[b=1]	.24[a]	.036	1	.000
	[R=2]*[x=3]*[b=2]	.36[a]	.038	1	.000
	[R=3]*[x=1]*[b=1]	-.23[a]	.038	1	.000
	[R=3]*[x=1]*[b=2]	.22[a]	.033	1	.000
	[R=3]*[x=2]*[b=1]	.13	.039	1	.131
	[R=3]*[x=2]*[b=2]	.32[a]	.035	1	.000
	[R=3]*[x=3]*[b=1]	.34[a]	.038	1	.000
	[R=3]*[x=3]*[b=2]	.36[a]	.039	1	.000

Pairwise Comparisons

(I) R*x*b	(J) R*x*b	95% Wald Confidence Interval for Difference	
		Lower	Upper
[R=2]*[x=1]*[b=1]	[R=1]*[x=1]*[b=1]	-.09	.01
	[R=1]*[x=1]*[b=2]	.12	.35
	[R=1]*[x=2]*[b=1]	-.03	.09
	[R=1]*[x=2]*[b=2]	.44	.68
	[R=1]*[x=3]*[b=1]	.22	.49
	[R=1]*[x=3]*[b=2]	.64	.84
	[R=2]*[x=1]*[b=2]	.34	.60
	[R=2]*[x=2]*[b=1]	.08	.27
	[R=2]*[x=2]*[b=2]	.68	.87
	[R=2]*[x=3]*[b=1]	.60	.81
	[R=2]*[x=3]*[b=2]	.74	.92
	[R=3]*[x=1]*[b=1]	.13	.35
	[R=3]*[x=1]*[b=2]	.58	.80
	[R=3]*[x=2]*[b=1]	.48	.72
	[R=3]*[x=2]*[b=2]	.71	.89
	[R=3]*[x=3]*[b=1]	.72	.90
	[R=3]*[x=3]*[b=2]	.74	.92
[R=2]*[x=1]*[b=2]	[R=1]*[x=1]*[b=1]	-.65	-.38
	[R=1]*[x=1]*[b=2]	-.36	-.11
	[R=1]*[x=2]*[b=1]	-.58	-.31
	[R=1]*[x=2]*[b=2]	-.03	.20
	[R=1]*[x=3]*[b=1]	-.26	.03
	[R=1]*[x=3]*[b=2]	.14	.39
	[R=2]*[x=1]*[b=1]	-.60	-.34
	[R=2]*[x=2]*[b=1]	-.42	-.17
	[R=2]*[x=2]*[b=2]	.18	.42
	[R=2]*[x=3]*[b=1]	.11	.37
	[R=2]*[x=3]*[b=2]	.22	.50
	[R=3]*[x=1]*[b=1]	-.37	-.10
	[R=3]*[x=1]*[b=2]	.10	.34
	[R=3]*[x=2]*[b=1]	-.01	.27
	[R=3]*[x=2]*[b=2]	.20	.45
	[R=3]*[x=3]*[b=1]	.20	.48
	[R=3]*[x=3]*[b=2]	.22	.50

Pairwise Comparisons

(I) R*x*b	(J) R*x*b	Mean Difference (I-J)	Std. Error	df	Bonferroni Sig.
[R=2]*[x=2]*[b=1]	[R=1]*[x=1]*[b=1]	-.22[a]	.030	1	.000
	[R=1]*[x=1]*[b=2]	.06	.032	1	1.000
	[R=1]*[x=2]*[b=1]	-.15[a]	.027	1	.000
	[R=1]*[x=2]*[b=2]	.38[a]	.034	1	.000
	[R=1]*[x=3]*[b=1]	.18[a]	.035	1	.000
	[R=1]*[x=3]*[b=2]	.56[a]	.034	1	.000
	[R=2]*[x=1]*[b=1]	-.18[a]	.027	1	.000
	[R=2]*[x=1]*[b=2]	.30[a]	.034	1	.000
	[R=2]*[x=2]*[b=2]	.60[a]	.033	1	.000
	[R=2]*[x=3]*[b=1]	.53[a]	.032	1	.000
	[R=2]*[x=3]*[b=2]	.66[a]	.035	1	.000
	[R=3]*[x=1]*[b=1]	.06	.028	1	1.000
	[R=3]*[x=1]*[b=2]	.52[a]	.035	1	.000
	[R=3]*[x=2]*[b=1]	.43[a]	.033	1	.000
	[R=3]*[x=2]*[b=2]	.62[a]	.033	1	.000
	[R=3]*[x=3]*[b=1]	.64[a]	.034	1	.000
	[R=3]*[x=3]*[b=2]	.65[a]	.035	1	.000
[R=2]*[x=2]*[b=2]	[R=1]*[x=1]*[b=1]	-.81[a]	.025	1	.000
	[R=1]*[x=1]*[b=2]	-.54[a]	.035	1	.000
	[R=1]*[x=2]*[b=1]	-.74[a]	.028	1	.000
	[R=1]*[x=2]*[b=2]	-.21[a]	.030	1	.000
	[R=1]*[x=3]*[b=1]	-.42[a]	.038	1	.000
	[R=1]*[x=3]*[b=2]	-.03	.023	1	1.000
	[R=2]*[x=1]*[b=1]	-.77[a]	.027	1	.000
	[R=2]*[x=1]*[b=2]	-.30[a]	.034	1	.000
	[R=2]*[x=2]*[b=1]	-.60[a]	.033	1	.000
	[R=2]*[x=3]*[b=1]	-.07	.024	1	.960
	[R=2]*[x=3]*[b=2]	.06	.017	1	.085
	[R=3]*[x=1]*[b=1]	-.53[a]	.036	1	.000
	[R=3]*[x=1]*[b=2]	-.08	.023	1	.062
	[R=3]*[x=2]*[b=1]	-.17[a]	.029	1	.000
	[R=3]*[x=2]*[b=2]	.02	.011	1	1.000
	[R=3]*[x=3]*[b=1]	.04	.019	1	1.000
	[R=3]*[x=3]*[b=2]	.06	.018	1	.242

Pairwise Comparisons

(I) R*x*b	(J) R*x*b	95% Wald Confidence Interval for Difference Lower	Upper
[R=2]*[x=2]*[b=1]	[R=1]*[x=1]*[b=1]	-.32	-.11
	[R=1]*[x=1]*[b=2]	-.05	.18
	[R=1]*[x=2]*[b=1]	-.24	-.05
	[R=1]*[x=2]*[b=2]	.26	.51
	[R=1]*[x=3]*[b=1]	.05	.31
	[R=1]*[x=3]*[b=2]	.44	.69
	[R=2]*[x=1]*[b=1]	-.27	-.08
	[R=2]*[x=1]*[b=2]	.17	.42
	[R=2]*[x=2]*[b=2]	.48	.72
	[R=2]*[x=3]*[b=1]	.42	.65
	[R=2]*[x=3]*[b=2]	.53	.78
	[R=3]*[x=1]*[b=1]	-.04	.17
	[R=3]*[x=1]*[b=2]	.39	.64
	[R=3]*[x=2]*[b=1]	.31	.54
	[R=3]*[x=2]*[b=2]	.50	.74
	[R=3]*[x=3]*[b=1]	.51	.76
	[R=3]*[x=3]*[b=2]	.53	.78
[R=2]*[x=2]*[b=2]	[R=1]*[x=1]*[b=1]	-.90	-.73
	[R=1]*[x=1]*[b=2]	-.66	-.41
	[R=1]*[x=2]*[b=1]	-.85	-.64
	[R=1]*[x=2]*[b=2]	-.32	-.11
	[R=1]*[x=3]*[b=1]	-.55	-.28
	[R=1]*[x=3]*[b=2]	-.12	.05
	[R=2]*[x=1]*[b=1]	-.87	-.68
	[R=2]*[x=1]*[b=2]	-.42	-.18
	[R=2]*[x=2]*[b=1]	-.72	-.48
	[R=2]*[x=3]*[b=1]	-.15	.02
	[R=2]*[x=3]*[b=2]	.00	.12
	[R=3]*[x=1]*[b=1]	-.66	-.40
	[R=3]*[x=1]*[b=2]	-.17	.00
	[R=3]*[x=2]*[b=1]	-.28	-.07
	[R=3]*[x=2]*[b=2]	-.02	.06
	[R=3]*[x=3]*[b=1]	-.03	.11
	[R=3]*[x=3]*[b=2]	-.01	.12

Pairwise Comparisons

(I) R*x*b	(J) R*x*b	Mean Difference (I-J)	Std. Error	df	Bonferroni Sig.
[R=2]*[x=3]*[b=1]	[R=1]*[x=1]*[b=1]	-.75[a]	.028	1	.000
	[R=1]*[x=1]*[b=2]	-.47[a]	.037	1	.000
	[R=1]*[x=2]*[b=1]	-.68[a]	.031	1	.000
	[R=1]*[x=2]*[b=2]	-.15[a]	.034	1	.002
	[R=1]*[x=3]*[b=1]	-.35[a]	.035	1	.000
	[R=1]*[x=3]*[b=2]	.03	.026	1	1.000
	[R=2]*[x=1]*[b=1]	-.71[a]	.029	1	.000
	[R=2]*[x=1]*[b=2]	-.24[a]	.036	1	.000
	[R=2]*[x=2]*[b=1]	-.53[a]	.032	1	.000
	[R=2]*[x=2]*[b=2]	.07	.024	1	.960
	[R=2]*[x=3]*[b=2]	.12[a]	.024	1	.000
	[R=3]*[x=1]*[b=1]	-.47[a]	.036	1	.000
	[R=3]*[x=1]*[b=2]	-.02	.029	1	1.000
	[R=3]*[x=2]*[b=1]	-.11	.030	1	.054
	[R=3]*[x=2]*[b=2]	.09	.025	1	.055
	[R=3]*[x=3]*[b=1]	.10[a]	.023	1	.001
	[R=3]*[x=3]*[b=2]	.12[a]	.025	1	.000
[R=2]*[x=3]*[b=2]	[R=1]*[x=1]*[b=1]	-.87[a]	.021	1	.000
	[R=1]*[x=1]*[b=2]	-.60[a]	.037	1	.000
	[R=1]*[x=2]*[b=1]	-.80[a]	.027	1	.000
	[R=1]*[x=2]*[b=2]	-.27[a]	.035	1	.000
	[R=1]*[x=3]*[b=1]	-.48[a]	.041	1	.000
	[R=1]*[x=3]*[b=2]	-.09[a]	.023	1	.006
	[R=2]*[x=1]*[b=1]	-.83[a]	.025	1	.000
	[R=2]*[x=1]*[b=2]	-.36[a]	.038	1	.000
	[R=2]*[x=2]*[b=1]	-.66[a]	.035	1	.000
	[R=2]*[x=2]*[b=2]	-.06	.017	1	.085
	[R=2]*[x=3]*[b=1]	-.12[a]	.024	1	.000
	[R=3]*[x=1]*[b=1]	-.59[a]	.038	1	.000
	[R=3]*[x=1]*[b=2]	-.14[a]	.027	1	.000
	[R=3]*[x=2]*[b=1]	-.23[a]	.032	1	.000
	[R=3]*[x=2]*[b=2]	-.04	.017	1	1.000
	[R=3]*[x=3]*[b=1]	-.02	.011	1	1.000
	[R=3]*[x=3]*[b=2]	.00	.012	1	1.000

Pairwise Comparisons

(I) R*x*b	(J) R*x*b	95% Wald Confidence Interval for Difference Lower	Upper
[R=2]*[x=3]*[b=1]	[R=1]*[x=1]*[b=1]	-.85	-.65
	[R=1]*[x=1]*[b=2]	-.61	-.34
	[R=1]*[x=2]*[b=1]	-.79	-.57
	[R=1]*[x=2]*[b=2]	-.27	-.03
	[R=1]*[x=3]*[b=1]	-.48	-.23
	[R=1]*[x=3]*[b=2]	-.06	.13
	[R=2]*[x=1]*[b=1]	-.81	-.60
	[R=2]*[x=1]*[b=2]	-.37	-.11
	[R=2]*[x=2]*[b=1]	-.65	-.42
	[R=2]*[x=2]*[b=2]	-.02	.15
	[R=2]*[x=3]*[b=2]	.04	.21
	[R=3]*[x=1]*[b=1]	-.60	-.34
	[R=3]*[x=1]*[b=2]	-.12	.09
	[R=3]*[x=2]*[b=1]	-.21	.00
	[R=3]*[x=2]*[b=2]	.00	.18
	[R=3]*[x=3]*[b=1]	.02	.19
	[R=3]*[x=3]*[b=2]	.03	.21
[R=2]*[x=3]*[b=2]	[R=1]*[x=1]*[b=1]	-.95	-.80
	[R=1]*[x=1]*[b=2]	-.73	-.46
	[R=1]*[x=2]*[b=1]	-.90	-.71
	[R=1]*[x=2]*[b=2]	-.40	-.15
	[R=1]*[x=3]*[b=1]	-.62	-.33
	[R=1]*[x=3]*[b=2]	-.17	-.01
	[R=2]*[x=1]*[b=1]	-.92	-.74
	[R=2]*[x=1]*[b=2]	-.50	-.22
	[R=2]*[x=2]*[b=1]	-.78	-.53
	[R=2]*[x=2]*[b=2]	-.12	.00
	[R=2]*[x=3]*[b=1]	-.21	-.04
	[R=3]*[x=1]*[b=1]	-.73	-.46
	[R=3]*[x=1]*[b=2]	-.24	-.04
	[R=3]*[x=2]*[b=1]	-.35	-.12
	[R=3]*[x=2]*[b=2]	-.10	.02
	[R=3]*[x=3]*[b=1]	-.06	.02
	[R=3]*[x=3]*[b=2]	-.05	.04

Pairwise Comparisons

(I) R*x*b	(J) R*x*b	Mean Difference (I-J)	Std. Error	df	Bonferroni Sig.
[R=3]*[x=1]*[b=1]	[R=1]*[x=1]*[b=1]	-.28[a]	.033	1	.000
	[R=1]*[x=1]*[b=2]	.00	.036	1	1.000
	[R=1]*[x=2]*[b=1]	-.21[a]	.033	1	.000
	[R=1]*[x=2]*[b=2]	.32[a]	.041	1	.000
	[R=1]*[x=3]*[b=1]	.12	.041	1	.661
	[R=1]*[x=3]*[b=2]	.50[a]	.039	1	.000
	[R=2]*[x=1]*[b=1]	-.24[a]	.031	1	.000
	[R=2]*[x=1]*[b=2]	.23[a]	.038	1	.000
	[R=2]*[x=2]*[b=1]	-.06	.028	1	1.000
	[R=2]*[x=2]*[b=2]	.53[a]	.036	1	.000
	[R=2]*[x=3]*[b=1]	.47[a]	.036	1	.000
	[R=2]*[x=3]*[b=2]	.59[a]	.038	1	.000
	[R=3]*[x=1]*[b=2]	.45[a]	.033	1	.000
	[R=3]*[x=2]*[b=1]	.36[a]	.031	1	.000
	[R=3]*[x=2]*[b=2]	.56[a]	.036	1	.000
	[R=3]*[x=3]*[b=1]	.57[a]	.037	1	.000
	[R=3]*[x=3]*[b=2]	.59[a]	.037	1	.000
[R=3]*[x=1]*[b=2]	[R=1]*[x=1]*[b=1]	-.73[a]	.029	1	.000
	[R=1]*[x=1]*[b=2]	-.45[a]	.035	1	.000
	[R=1]*[x=2]*[b=1]	-.66[a]	.033	1	.000
	[R=1]*[x=2]*[b=2]	-.13[a]	.033	1	.010
	[R=1]*[x=3]*[b=1]	-.34[a]	.039	1	.000
	[R=1]*[x=3]*[b=2]	.05	.030	1	1.000
	[R=2]*[x=1]*[b=1]	-.69[a]	.030	1	.000
	[R=2]*[x=1]*[b=2]	-.22[a]	.033	1	.000
	[R=2]*[x=2]*[b=1]	-.52[a]	.035	1	.000
	[R=2]*[x=2]*[b=2]	.08	.023	1	.062
	[R=2]*[x=3]*[b=1]	.02	.029	1	1.000
	[R=2]*[x=3]*[b=2]	.14[a]	.027	1	.000
	[R=3]*[x=1]*[b=1]	-.45[a]	.033	1	.000
	[R=3]*[x=2]*[b=1]	-.09	.030	1	.388
	[R=3]*[x=2]*[b=2]	.10[a]	.021	1	.000
	[R=3]*[x=3]*[b=1]	.12[a]	.027	1	.001
	[R=3]*[x=3]*[b=2]	.14[a]	.026	1	.000

Pairwise Comparisons

(I) R*x*b	(J) R*x*b	95% Wald Confidence Interval for Difference	
		Lower	Upper
[R=3]*[x=1]*[b=1]	[R=1]*[x=1]*[b=1]	-.40	-.16
	[R=1]*[x=1]*[b=2]	-.13	.12
	[R=1]*[x=2]*[b=1]	-.33	-.09
	[R=1]*[x=2]*[b=2]	.17	.47
	[R=1]*[x=3]*[b=1]	-.03	.26
	[R=1]*[x=3]*[b=2]	.36	.64
	[R=2]*[x=1]*[b=1]	-.35	-.13
	[R=2]*[x=1]*[b=2]	.10	.37
	[R=2]*[x=2]*[b=1]	-.17	.04
	[R=2]*[x=2]*[b=2]	.40	.66
	[R=2]*[x=3]*[b=1]	.34	.60
	[R=2]*[x=3]*[b=2]	.46	.73
	[R=3]*[x=1]*[b=2]	.33	.57
	[R=3]*[x=2]*[b=1]	.25	.47
	[R=3]*[x=2]*[b=2]	.43	.68
	[R=3]*[x=3]*[b=1]	.44	.70
	[R=3]*[x=3]*[b=2]	.45	.72
[R=3]*[x=1]*[b=2]	[R=1]*[x=1]*[b=1]	-.84	-.63
	[R=1]*[x=1]*[b=2]	-.58	-.33
	[R=1]*[x=2]*[b=1]	-.78	-.54
	[R=1]*[x=2]*[b=2]	-.25	-.01
	[R=1]*[x=3]*[b=1]	-.48	-.19
	[R=1]*[x=3]*[b=2]	-.06	.16
	[R=2]*[x=1]*[b=1]	-.80	-.58
	[R=2]*[x=1]*[b=2]	-.34	-.10
	[R=2]*[x=2]*[b=1]	-.64	-.39
	[R=2]*[x=2]*[b=2]	.00	.17
	[R=2]*[x=3]*[b=1]	-.09	.12
	[R=2]*[x=3]*[b=2]	.04	.24
	[R=3]*[x=1]*[b=1]	-.57	-.33
	[R=3]*[x=2]*[b=1]	-.20	.02
	[R=3]*[x=2]*[b=2]	.03	.18
	[R=3]*[x=3]*[b=1]	.02	.22
	[R=3]*[x=3]*[b=2]	.05	.23

Pairwise Comparisons

(I) R*x*b	(J) R*x*b	Mean Difference (I-J)	Std. Error	df	Bonferroni Sig.
[R=3]*[x=2]*[b=1]	[R=1]*[x=1]*[b=1]	-.64[a]	.034	1	.000
	[R=1]*[x=1]*[b=2]	-.36[a]	.037	1	.000
	[R=1]*[x=2]*[b=1]	-.57[a]	.034	1	.000
	[R=1]*[x=2]*[b=2]	-.04	.037	1	1.000
	[R=1]*[x=3]*[b=1]	-.25[a]	.038	1	.000
	[R=1]*[x=3]*[b=2]	.14[a]	.035	1	.010
	[R=2]*[x=1]*[b=1]	-.60[a]	.033	1	.000
	[R=2]*[x=1]*[b=2]	-.13	.039	1	.131
	[R=2]*[x=2]*[b=1]	-.43[a]	.033	1	.000
	[R=2]*[x=2]*[b=2]	.17[a]	.029	1	.000
	[R=2]*[x=3]*[b=1]	.11	.030	1	.054
	[R=2]*[x=3]*[b=2]	.23[a]	.032	1	.000
	[R=3]*[x=1]*[b=1]	-.36[a]	.031	1	.000
	[R=3]*[x=1]*[b=2]	.09	.030	1	.388
	[R=3]*[x=2]*[b=2]	.19[a]	.029	1	.000
	[R=3]*[x=3]*[b=1]	.21[a]	.031	1	.000
	[R=3]*[x=3]*[b=2]	.23[a]	.033	1	.000
[R=3]*[x=2]*[b=2]	[R=1]*[x=1]*[b=1]	-.84[a]	.023	1	.000
	[R=1]*[x=1]*[b=2]	-.56[a]	.035	1	.000
	[R=1]*[x=2]*[b=1]	-.77[a]	.027	1	.000
	[R=1]*[x=2]*[b=2]	-.24[a]	.031	1	.000
	[R=1]*[x=3]*[b=1]	-.44[a]	.038	1	.000
	[R=1]*[x=3]*[b=2]	-.06	.024	1	1.000
	[R=2]*[x=1]*[b=1]	-.80[a]	.025	1	.000
	[R=2]*[x=1]*[b=2]	-.32[a]	.035	1	.000
	[R=2]*[x=2]*[b=1]	-.62[a]	.033	1	.000
	[R=2]*[x=2]*[b=2]	-.02	.011	1	1.000
	[R=2]*[x=3]*[b=1]	-.09	.025	1	.055
	[R=2]*[x=3]*[b=2]	.04	.017	1	1.000
	[R=3]*[x=1]*[b=1]	-.56[a]	.036	1	.000
	[R=3]*[x=1]*[b=2]	-.10[a]	.021	1	.000
	[R=3]*[x=2]*[b=1]	-.19[a]	.029	1	.000
	[R=3]*[x=3]*[b=1]	.02	.017	1	1.000
	[R=3]*[x=3]*[b=2]	.03	.013	1	1.000

Pairwise Comparisons

(I) R*x*b	(J) R*x*b	95% Wald Confidence Interval for Difference Lower	Upper
[R=3]*[x=2]*[b=1]	[R=1]*[x=1]*[b=1]	-.76	-.52
	[R=1]*[x=1]*[b=2]	-.50	-.23
	[R=1]*[x=2]*[b=1]	-.69	-.45
	[R=1]*[x=2]*[b=2]	-.17	.09
	[R=1]*[x=3]*[b=1]	-.38	-.11
	[R=1]*[x=3]*[b=2]	.01	.26
	[R=2]*[x=1]*[b=1]	-.72	-.48
	[R=2]*[x=1]*[b=2]	-.27	.01
	[R=2]*[x=2]*[b=1]	-.54	-.31
	[R=2]*[x=2]*[b=2]	.07	.28
	[R=2]*[x=3]*[b=1]	.00	.21
	[R=2]*[x=3]*[b=2]	.12	.35
	[R=3]*[x=1]*[b=1]	-.47	-.25
	[R=3]*[x=1]*[b=2]	-.02	.20
	[R=3]*[x=2]*[b=2]	.09	.30
	[R=3]*[x=3]*[b=1]	.10	.32
	[R=3]*[x=3]*[b=2]	.11	.34
[R=3]*[x=2]*[b=2]	[R=1]*[x=1]*[b=1]	-.92	-.75
	[R=1]*[x=1]*[b=2]	-.68	-.43
	[R=1]*[x=2]*[b=1]	-.86	-.67
	[R=1]*[x=2]*[b=2]	-.35	-.12
	[R=1]*[x=3]*[b=1]	-.58	-.30
	[R=1]*[x=3]*[b=2]	-.14	.03
	[R=2]*[x=1]*[b=1]	-.89	-.71
	[R=2]*[x=1]*[b=2]	-.45	-.20
	[R=2]*[x=2]*[b=1]	-.74	-.50
	[R=2]*[x=2]*[b=2]	-.06	.02
	[R=2]*[x=3]*[b=1]	-.18	.00
	[R=2]*[x=3]*[b=2]	-.02	.10
	[R=3]*[x=1]*[b=1]	-.68	-.43
	[R=3]*[x=1]*[b=2]	-.18	-.03
	[R=3]*[x=2]*[b=1]	-.30	-.09
	[R=3]*[x=3]*[b=1]	-.04	.08
	[R=3]*[x=3]*[b=2]	-.01	.08

Pairwise Comparisons

(I) R*x*b	(J) R*x*b	Mean Difference (I-J)	Std. Error	df	Bonferroni Sig.
[R=3]*[x=3]*[b=1]	[R=1]*[x=1]*[b=1]	-.85[a]	.022	1	.000
	[R=1]*[x=1]*[b=2]	-.58[a]	.038	1	.000
	[R=1]*[x=2]*[b=1]	-.78[a]	.027	1	.000
	[R=1]*[x=2]*[b=2]	-.25[a]	.034	1	.000
	[R=1]*[x=3]*[b=1]	-.46[a]	.040	1	.000
	[R=1]*[x=3]*[b=2]	-.07	.023	1	.281
	[R=2]*[x=1]*[b=1]	-.81[a]	.025	1	.000
	[R=2]*[x=1]*[b=2]	-.34[a]	.038	1	.000
	[R=2]*[x=2]*[b=1]	-.64[a]	.034	1	.000
	[R=2]*[x=2]*[b=2]	-.04	.019	1	1.000
	[R=2]*[x=3]*[b=1]	-.10[a]	.023	1	.001
	[R=2]*[x=3]*[b=2]	.02	.011	1	1.000
	[R=3]*[x=1]*[b=1]	-.57[a]	.037	1	.000
	[R=3]*[x=1]*[b=2]	-.12[a]	.027	1	.001
	[R=3]*[x=2]*[b=1]	-.21[a]	.031	1	.000
	[R=3]*[x=2]*[b=2]	-.02	.017	1	1.000
	[R=3]*[x=3]*[b=2]	.02	.014	1	1.000
[R=3]*[x=3]*[b=2]	[R=1]*[x=1]*[b=1]	-.87[a]	.021	1	.000
	[R=1]*[x=1]*[b=2]	-.59[a]	.037	1	.000
	[R=1]*[x=2]*[b=1]	-.80[a]	.027	1	.000
	[R=1]*[x=2]*[b=2]	-.27[a]	.035	1	.000
	[R=1]*[x=3]*[b=1]	-.47[a]	.040	1	.000
	[R=1]*[x=3]*[b=2]	-.09[a]	.024	1	.026
	[R=2]*[x=1]*[b=1]	-.83[a]	.024	1	.000
	[R=2]*[x=1]*[b=2]	-.36[a]	.039	1	.000
	[R=2]*[x=2]*[b=1]	-.65[a]	.035	1	.000
	[R=2]*[x=2]*[b=2]	-.06	.018	1	.242
	[R=2]*[x=3]*[b=1]	-.12[a]	.025	1	.000
	[R=2]*[x=3]*[b=2]	.00	.012	1	1.000
	[R=3]*[x=1]*[b=1]	-.59[a]	.037	1	.000
	[R=3]*[x=1]*[b=2]	-.14[a]	.026	1	.000
	[R=3]*[x=2]*[b=1]	-.23[a]	.033	1	.000
	[R=3]*[x=2]*[b=2]	-.03	.013	1	1.000
	[R=3]*[x=3]*[b=1]	-.02	.014	1	1.000

Pairwise Comparisons

(I) R*x*b	(J) R*x*b	95% Wald Confidence Interval for Difference	
		Lower	Upper
[R=3]*[x=3]*[b=1]	[R=1]*[x=1]*[b=1]	-.93	-.77
	[R=1]*[x=1]*[b=2]	-.71	-.44
	[R=1]*[x=2]*[b=1]	-.88	-.69
	[R=1]*[x=2]*[b=2]	-.38	-.13
	[R=1]*[x=3]*[b=1]	-.60	-.31
	[R=1]*[x=3]*[b=2]	-.16	.01
	[R=2]*[x=1]*[b=1]	-.90	-.72
	[R=2]*[x=1]*[b=2]	-.48	-.20
	[R=2]*[x=2]*[b=1]	-.76	-.51
	[R=2]*[x=2]*[b=2]	-.11	.03
	[R=2]*[x=3]*[b=1]	-.19	-.02
	[R=2]*[x=3]*[b=2]	-.02	.06
	[R=3]*[x=1]*[b=1]	-.70	-.44
	[R=3]*[x=1]*[b=2]	-.22	-.02
	[R=3]*[x=2]*[b=1]	-.32	-.10
	[R=3]*[x=2]*[b=2]	-.08	.04
	[R=3]*[x=3]*[b=2]	-.03	.07
[R=3]*[x=3]*[b=2]	[R=1]*[x=1]*[b=1]	-.95	-.79
	[R=1]*[x=1]*[b=2]	-.72	-.46
	[R=1]*[x=2]*[b=1]	-.90	-.70
	[R=1]*[x=2]*[b=2]	-.39	-.14
	[R=1]*[x=3]*[b=1]	-.62	-.33
	[R=1]*[x=3]*[b=2]	-.17	.00
	[R=2]*[x=1]*[b=1]	-.92	-.74
	[R=2]*[x=1]*[b=2]	-.50	-.22
	[R=2]*[x=2]*[b=1]	-.78	-.53
	[R=2]*[x=2]*[b=2]	-.12	.01
	[R=2]*[x=3]*[b=1]	-.21	-.03
	[R=2]*[x=3]*[b=2]	-.04	.05
	[R=3]*[x=1]*[b=1]	-.72	-.45
	[R=3]*[x=1]*[b=2]	-.23	-.05
	[R=3]*[x=2]*[b=1]	-.34	-.11
	[R=3]*[x=2]*[b=2]	-.08	.01
	[R=3]*[x=3]*[b=1]	-.07	.03

Pairwise comparisons of estimated marginal means based on the original scale of dependent variable y

a. The mean difference is significant at the .05 level.

Overall Test Results

Wald Chi-Square	df	Sig.
1900.488	17	.000

The Wald chi-square tests the effect of R*x*b. This test is based on the linearly independent pairwise comparisons among the estimated marginal means.

```
GENLIN y (REFERENCE=FIRST) BY R x b gender_g income_g (ORDER=ASCENDING) WITH age
/MODEL R x b gender_g income_g age R*x R*x*b INTERCEPT=YES
DISTRIBUTION=BINOMIAL LINK=LOGIT
/CRITERIA METHOD=FISHER(1) SCALE=1 MAXITERATIONS=100 MAXSTEPHALVING=5 PCONVERGE=1E-006 (ABSOLUTE)
SINGULAR=1E-012 ANALYSISTYPE=3(WALD) CILEVEL=95 LIKELIHOOD=FULL
/EMMEANS TABLES=R SCALE=ORIGINAL COMPARE=R CONTRAST=PAIRWISE PADJUST=BONFERRONI
/EMMEANS TABLES=x SCALE=ORIGINAL COMPARE=x CONTRAST=PAIRWISE PADJUST=BONFERRONI
/EMMEANS TABLES=b SCALE=ORIGINAL COMPARE=b CONTRAST=PAIRWISE PADJUST=BONFERRONI
/EMMEANS TABLES=R*x SCALE=ORIGINAL COMPARE=R CONTRAST=PAIRWISE PADJUST=BONFERRONI
/EMMEANS TABLES=R*x SCALE=ORIGINAL COMPARE=x CONTRAST=PAIRWISE PADJUST=BONFERRONI
/EMMEANS TABLES=R*x*b SCALE=ORIGINAL COMPARE=R*x*b CONTRAST=PAIRWISE PADJUST=BONFERRONI
/REPEATED SUBJECT=id WITHINSUBJECT=R*x*b SORT=YES CORRTYPE=AR(1) ADJUSTCORR=YES COVB=ROBUST
MAXITERATIONS=100 PCONVERGE=1e-006 (ABSOLUTE) UPDATECORR=1
/MISSING CLASSMISSING=EXCLUDE
/PRINT CPS DESCRIPTIVES MODELINFO FIT SUMMARY SOLUTION (EXPONENTIATED) WORKINGCORR.
```

Generalized Linear Models

Notes

Output Created		11-JUN-2026 13:41:51
Comments		
Input	Data	E : \ 1 . \ 2 .<br>\ 2 . \ 4 .<br>\ 1 .<br>\behavior sciences\<br>\ .<br>sav
	Active Dataset	1
	Filter	<none>
	Weight	<none>
	Split File	<none>
	N of Rows in Working Data File	4536
Missing Value Handling	Definition of Missing	User-defined missing values for factor, subject and within-subject variables are treated as missing.
	Cases Used	Statistics are based on cases with valid data for all variables in the model.
Weight Handling		not applicable

Notes

Syntax	GENLIN y (REFERENCE=FIRST) BY R x b gender_g income_g (ORDER=ASCENDING) WITH age<br>/MODEL R x b gender_g income_g age R*x R*x*b INTERCEPT=YES<br><br>DISTRIBUTION=BINOMIAL LINK=LOGIT<br>/CRITERIA METHOD=FISHER(1) SCALE=1 MAXITERATIONS=100 MAXSTEPHALVING=5 PCONVERGE=1E-006 (ABSOLUTE)<br>SINGULAR=1E-012 ANALYSISTYPE=3 (WALD) CILEVEL=95 LIKELIHOOD=FULL<br>/EMMEANS TABLES=R SCALE=ORIGINAL COMPARE=R CONTRAST=PAIRWISE PADJUST=BONFERRONI<br>/EMMEANS TABLES=x SCALE=ORIGINAL COMPARE=x CONTRAST=PAIRWISE PADJUST=BONFERRONI<br>/EMMEANS TABLES=b SCALE=ORIGINAL COMPARE=b CONTRAST=PAIRWISE PADJUST=BONFERRONI<br>/EMMEANS TABLES=R*x SCALE=ORIGINAL COMPARE=R CONTRAST=PAIRWISE PADJUST=BONFERRONI<br>/EMMEANS TABLES=R*x SCALE=ORIGINAL COMPARE=x CONTRAST=PAIRWISE PADJUST=BONFERRONI<br>/EMMEANS TABLES=R*x*b SCALE=ORIGINAL COMPARE=R*x*b CONTRAST=PAIRWISE PADJUST=BONFERRONI<br>/REPEATED SUBJECT=id WITHINSUBJECT=R*x*b SORT=YES CORRTYPE=AR(1) ADJUSTCORR=YES COVB=ROBUST<br>MAXITERATIONS=100 PCONVERGE=1e-006 (ABSOLUTE)

Notes

Resources	Processor Time	00:00:00.33
	Elapsed Time	00:00:00.84

Model Information

Dependent Variable		y[a]
Probability Distribution		Binomial
Link Function		Logit
Subject Effect	1	id
Within-Subject Effect	1	R
	2	x
	3	b
Working Correlation Matrix Structure		AR(1)

a. The procedure models 1 as the response, treating 0 as the reference category.

Case Processing Summary

	N	Percent
Included	4536	100.0%
Excluded	0	0.0%
Total	4536	100.0%

Correlated Data Summary

Number of Levels	Subject Effect	id	252
	Within-Subject Effect	R	3
		x	3
		b	2
Number of Subjects			252
Number of Measurements per Subject	Minimum		18
	Maximum		18
Correlation Matrix Dimension			18

Categorical Variable Information

			N	Percent
Dependent Variable	y	0	2753	60.7%
		1	1783	39.3%
		Total	4536	100.0%
Factor	R	kin	1512	33.3%
		acquaintance	1512	33.3%
		general other	1512	33.3%
		Total	4536	100.0%
	x	2000	1512	33.3%
		20,000	1512	33.3%
		200,000	1512	33.3%
		Total	4536	100.0%
	b	H	2268	50.0%
		L	2268	50.0%
		Total	4536	100.0%
	gender_g	female	2556	56.3%
		male	1980	43.7%
		Total	4536	100.0%
	income_g	1000RMB	810	17.9%
		1000-3000RMB	1476	32.5%
		3000-5000RMB	972	21.4%
		5000-8000RMB	576	12.7%
		8000-12000RMB	504	11.1%
		12000-15000RMB	90	2.0%
		15000RMB	108	2.4%
		Total	4536	100.0%

Continuous Variable Information

		N	Minimum	Maximum	Mean	Std. Deviation
Covariate	age	4536	16	69	37.04	13.196

Goodness of Fit[a]

	Value
Quasi Likelihood under Independence Model Criterion (QIC)[b]	4256.201
Corrected Quasi Likelihood under Independence Model Criterion (QICC)[b]	4199.005

Dependent Variable: y
Model: (Intercept), R, x, b, gender_g, income_g, age, R * x, R * x * b

a. Information criteria are in smaller-is-better form.

b. Computed using the full log quasi-likelihood function.

Tests of Model Effects

Source	Type III		
	Wald Chi-Square	df	Sig.
(Intercept)	14.455	1	.000
R	195.375	2	.000
x	262.163	2	.000
b	249.017	1	.000
gender_g	.023	1	.880
income_g	5.308	6	.505
age	4.561	1	.033
R * x	27.157	4	.000
R * x * b	124.298	8	.000

Dependent Variable: y
Model: (Intercept), R, x, b, gender_g, income_g, age, R * x, R * x * b

Parameter Estimates

Parameter	B	Std. Error	95% Wald Confidence Interval		Hypothesis Test
			Lower	Upper	Wald Chi-Square
(Intercept)	-3.847	.6805	-5.181	-2.514	31.965
[R=1]	1.012	.2594	.503	1.520	15.204
[R=2]	-.071	.2188	-.499	.358	.104
[R=3]	0[a]	.	.	.	.
[x=1]	1.351	.2379	.885	1.818	32.278
[x=2]	.473	.1826	.115	.830	6.700
[x=3]	0[a]	.	.	.	.
[b=1]	.257	.2199	-.174	.688	1.361
[b=2]	0[a]	.	.	.	.
[gender_g=0]	-.027	.1791	-.378	.324	.023
[gender_g=1]	0[a]	.	.	.	.
[income_g=1]	.330	.6365	-.917	1.578	.269
[income_g=2]	.645	.6169	-.564	1.854	1.094
[income_g=3]	.541	.6146	-.663	1.746	.776
[income_g=4]	.593	.6170	-.616	1.802	.923
[income_g=5]	.450	.6252	-.775	1.675	.519
[income_g=6]	1.426	.7794	-.101	2.954	3.349
[income_g=7]	0[a]	.	.	.	.
age	.015	.0068	.001	.028	4.561
[R=1] * [x=1]	1.019	.2724	.485	1.552	13.978
[R=1] * [x=2]	.555	.2184	.127	.983	6.457
[R=1] * [x=3]	0[a]	.	.	.	.
[R=2] * [x=1]	1.139	.2538	.642	1.637	20.151
[R=2] * [x=2]	.314	.1903	-.059	.686	2.716
[R=2] * [x=3]	0[a]	.	.	.	.
[R=3] * [x=1]	0[a]	.	.	.	.
[R=3] * [x=2]	0[a]	.	.	.	.
[R=3] * [x=3]	0[a]	.	.	.	.
[R=1] * [x=1] * [b=1]	1.700	.3111	1.091	2.310	29.885
[R=1] * [x=1] * [b=2]	0[a]	.	.	.	.
[R=1] * [x=2] * [b=1]	2.270	.2912	1.699	2.840	60.751
[R=1] * [x=2] * [b=2]	0[a]	.	.	.	.
[R=1] * [x=3] * [b=1]	1.619	.2879	1.054	2.183	31.609
[R=1] * [x=3] * [b=2]	0[a]	.	.	.	.

Parameter Estimates

Parameter	Hypothesis Test		Exp(B)	95% Wald Confidence Interval for Exp(B)	
	df	Sig.		Lower	Upper
(Intercept)	1	.000	.021	.006	.081
[R=1]	1	.000	2.750	1.654	4.573
[R=2]	1	.747	.932	.607	1.431
[R=3]	.	.	1	.	.
[x=1]	1	.000	3.863	2.423	6.157
[x=2]	1	.010	1.604	1.122	2.294
[x=3]	.	.	1	.	.
[b=1]	1	.243	1.292	.840	1.989
[b=2]	.	.	1	.	.
[gender_g=0]	1	.880	.973	.685	1.383
[gender_g=1]	.	.	1	.	.
[income_g=1]	1	.604	1.391	.400	4.844
[income_g=2]	1	.296	1.907	.569	6.389
[income_g=3]	1	.378	1.718	.515	5.731
[income_g=4]	1	.337	1.809	.540	6.063
[income_g=5]	1	.471	1.569	.461	5.341
[income_g=6]	1	.067	4.164	.904	19.185
[income_g=7]	.	.	1	.	.
age	1	.033	1.015	1.001	1.028
[R=1] * [x=1]	1	.000	2.769	1.623	4.723
[R=1] * [x=2]	1	.011	1.742	1.135	2.672
[R=1] * [x=3]	.	.	1	.	.
[R=2] * [x=1]	1	.000	3.125	1.900	5.139
[R=2] * [x=2]	1	.099	1.368	.942	1.987
[R=2] * [x=3]	.	.	1	.	.
[R=3] * [x=1]	.	.	1	.	.
[R=3] * [x=2]	.	.	1	.	.
[R=3] * [x=3]	.	.	1	.	.
[R=1] * [x=1] * [b=1]	1	.000	5.476	2.977	10.075
[R=1] * [x=1] * [b=2]	.	.	1	.	.
[R=1] * [x=2] * [b=1]	1	.000	9.675	5.467	17.119
[R=1] * [x=2] * [b=2]	.	.	1	.	.
[R=1] * [x=3] * [b=1]	1	.000	5.046	2.870	8.871
[R=1] * [x=3] * [b=2]	.	.	1	.	.

Parameter Estimates

Parameter	B	Std. Error	95% Wald Confidence Interval Lower	95% Wald Confidence Interval Upper	Hypothesis Test Wald Chi-Square
[R=2] * [x=1] * [b=1]	2.156	.3046	1.559	2.753	50.080
[R=2] * [x=1] * [b=2]	0[a]	.	.	.	.
[R=2] * [x=2] * [b=1]	2.693	.2819	2.141	3.246	91.267
[R=2] * [x=2] * [b=2]	0[a]	.	.	.	.
[R=2] * [x=3] * [b=1]	1.053	.3275	.411	1.695	10.344
[R=2] * [x=3] * [b=2]	0[a]	.	.	.	.
[R=3] * [x=1] * [b=1]	1.760	.2513	1.268	2.253	49.057
[R=3] * [x=1] * [b=2]	0[a]	.	.	.	.
[R=3] * [x=2] * [b=1]	1.116	.2313	.662	1.569	23.265
[R=3] * [x=2] * [b=2]	0[a]	.	.	.	.
[R=3] * [x=3] * [b=1]	0[a]	.	.	.	.
[R=3] * [x=3] * [b=2]	0[a]	.	.	.	.
(Scale)	1				

Parameter Estimates

Parameter	Hypothesis Test df	Hypothesis Test Sig.	Exp(B)	95% Wald Confidence Interval for Exp(B) Lower	95% Wald Confidence Interval for Exp(B) Upper
[R=2] * [x=1] * [b=1]	1	.000	8.633	4.752	15.683
[R=2] * [x=1] * [b=2]	.	.	1	.	.
[R=2] * [x=2] * [b=1]	1	.000	14.780	8.505	25.682
[R=2] * [x=2] * [b=2]	.	.	1	.	.
[R=2] * [x=3] * [b=1]	1	.001	2.867	1.509	5.448
[R=2] * [x=3] * [b=2]	.	.	1	.	.
[R=3] * [x=1] * [b=1]	1	.000	5.815	3.553	9.517
[R=3] * [x=1] * [b=2]	.	.	1	.	.
[R=3] * [x=2] * [b=1]	1	.000	3.051	1.939	4.802
[R=3] * [x=2] * [b=2]	.	.	1	.	.
[R=3] * [x=3] * [b=1]	.	.	1	.	.
[R=3] * [x=3] * [b=2]	.	.	1	.	.
(Scale)					

Dependent Variable: y
Model: (Intercept), R, x, b, gender_g, income_g, age, R * x, R * x * b

a. Set to zero because this parameter is redundant.

Working Correlation Matrix[a]

Measurement	Measurement [R = 1]* [x = 1]* [b = 1]	[R = 1]* [x = 1]* [b = 2]	[R = 1]* [x = 2]* [b = 1]	[R = 1]* [x = 2]* [b = 2]
[R = 1]* [x = 1]* [b = 1]	1.000	.349	.122	.043
[R = 1]* [x = 1]* [b = 2]	.349	1.000	.349	.122
[R = 1]* [x = 2]* [b = 1]	.122	.349	1.000	.349
[R = 1]* [x = 2]* [b = 2]	.043	.122	.349	1.000
[R = 1]* [x = 3]* [b = 1]	.015	.043	.122	.349
[R = 1]* [x = 3]* [b = 2]	.005	.015	.043	.122
[R = 2]* [x = 1]* [b = 1]	.002	.005	.015	.043
[R = 2]* [x = 1]* [b = 2]	.001	.002	.005	.015
[R = 2]* [x = 2]* [b = 1]	.000	.001	.002	.005
[R = 2]* [x = 2]* [b = 2]	.000	.000	.001	.002
[R = 2]* [x = 3]* [b = 1]	.000	.000	.000	.001
[R = 2]* [x = 3]* [b = 2]	.000	.000	.000	.000
[R = 3]* [x = 1]* [b = 1]	.000	.000	.000	.000
[R = 3]* [x = 1]* [b = 2]	.000	.000	.000	.000
[R = 3]* [x = 2]* [b = 1]	.000	.000	.000	.000
[R = 3]* [x = 2]* [b = 2]	.000	.000	.000	.000
[R = 3]* [x = 3]* [b = 1]	.000	.000	.000	.000
[R = 3]* [x = 3]* [b = 2]	.000	.000	.000	.000

Working Correlation Matrix[a]

Measurement	Measurement: [R = 1]* [x = 3]* [b = 1]	[R = 1]* [x = 3]* [b = 2]	[R = 2]* [x = 1]* [b = 1]	[R = 2]* [x = 1]* [b = 2]
[R = 1]* [x = 1]* [b = 1]	.015	.005	.002	.001
[R = 1]* [x = 1]* [b = 2]	.043	.015	.005	.002
[R = 1]* [x = 2]* [b = 1]	.122	.043	.015	.005
[R = 1]* [x = 2]* [b = 2]	.349	.122	.043	.015
[R = 1]* [x = 3]* [b = 1]	1.000	.349	.122	.043
[R = 1]* [x = 3]* [b = 2]	.349	1.000	.349	.122
[R = 2]* [x = 1]* [b = 1]	.122	.349	1.000	.349
[R = 2]* [x = 1]* [b = 2]	.043	.122	.349	1.000
[R = 2]* [x = 2]* [b = 1]	.015	.043	.122	.349
[R = 2]* [x = 2]* [b = 2]	.005	.015	.043	.122
[R = 2]* [x = 3]* [b = 1]	.002	.005	.015	.043
[R = 2]* [x = 3]* [b = 2]	.001	.002	.005	.015
[R = 3]* [x = 1]* [b = 1]	.000	.001	.002	.005
[R = 3]* [x = 1]* [b = 2]	.000	.000	.001	.002
[R = 3]* [x = 2]* [b = 1]	.000	.000	.000	.001
[R = 3]* [x = 2]* [b = 2]	.000	.000	.000	.000
[R = 3]* [x = 3]* [b = 1]	.000	.000	.000	.000
[R = 3]* [x = 3]* [b = 2]	.000	.000	.000	.000

Working Correlation Matrix[a]

Measurement	Measurement [R = 2]* [x = 2]* [b = 1]	[R = 2]* [x = 2]* [b = 2]	[R = 2]* [x = 3]* [b = 1]	[R = 2]* [x = 3]* [b = 2]
[R = 1]* [x = 1]* [b = 1]	.000	.000	.000	.000
[R = 1]* [x = 1]* [b = 2]	.001	.000	.000	.000
[R = 1]* [x = 2]* [b = 1]	.002	.001	.000	.000
[R = 1]* [x = 2]* [b = 2]	.005	.002	.001	.000
[R = 1]* [x = 3]* [b = 1]	.015	.005	.002	.001
[R = 1]* [x = 3]* [b = 2]	.043	.015	.005	.002
[R = 2]* [x = 1]* [b = 1]	.122	.043	.015	.005
[R = 2]* [x = 1]* [b = 2]	.349	.122	.043	.015
[R = 2]* [x = 2]* [b = 1]	1.000	.349	.122	.043
[R = 2]* [x = 2]* [b = 2]	.349	1.000	.349	.122
[R = 2]* [x = 3]* [b = 1]	.122	.349	1.000	.349
[R = 2]* [x = 3]* [b = 2]	.043	.122	.349	1.000
[R = 3]* [x = 1]* [b = 1]	.015	.043	.122	.349
[R = 3]* [x = 1]* [b = 2]	.005	.015	.043	.122
[R = 3]* [x = 2]* [b = 1]	.002	.005	.015	.043
[R = 3]* [x = 2]* [b = 2]	.001	.002	.005	.015
[R = 3]* [x = 3]* [b = 1]	.000	.001	.002	.005
[R = 3]* [x = 3]* [b = 2]	.000	.000	.001	.002

Working Correlation Matrix[a]

Measurement	Measurement			
	[R = 3]* [x = 1]* [b = 1]	[R = 3]* [x = 1]* [b = 2]	[R = 3]* [x = 2]* [b = 1]	[R = 3]* [x = 2]* [b = 2]
[R = 1]* [x = 1]* [b = 1]	.000	.000	.000	.000
[R = 1]* [x = 1]* [b = 2]	.000	.000	.000	.000
[R = 1]* [x = 2]* [b = 1]	.000	.000	.000	.000
[R = 1]* [x = 2]* [b = 2]	.000	.000	.000	.000
[R = 1]* [x = 3]* [b = 1]	.000	.000	.000	.000
[R = 1]* [x = 3]* [b = 2]	.001	.000	.000	.000
[R = 2]* [x = 1]* [b = 1]	.002	.001	.000	.000
[R = 2]* [x = 1]* [b = 2]	.005	.002	.001	.000
[R = 2]* [x = 2]* [b = 1]	.015	.005	.002	.001
[R = 2]* [x = 2]* [b = 2]	.043	.015	.005	.002
[R = 2]* [x = 3]* [b = 1]	.122	.043	.015	.005
[R = 2]* [x = 3]* [b = 2]	.349	.122	.043	.015
[R = 3]* [x = 1]* [b = 1]	1.000	.349	.122	.043
[R = 3]* [x = 1]* [b = 2]	.349	1.000	.349	.122
[R = 3]* [x = 2]* [b = 1]	.122	.349	1.000	.349
[R = 3]* [x = 2]* [b = 2]	.043	.122	.349	1.000
[R = 3]* [x = 3]* [b = 1]	.015	.043	.122	.349
[R = 3]* [x = 3]* [b = 2]	.005	.015	.043	.122

Working Correlation Matrix[a]

Measurement	Measurement<br>[R = 3]* [x = 3]* [b = 1]	 [R = 3]* [x = 3]* [b = 2]
[R = 1]* [x = 1]* [b = 1]	.000	.000
[R = 1]* [x = 1]* [b = 2]	.000	.000
[R = 1]* [x = 2]* [b = 1]	.000	.000
[R = 1]* [x = 2]* [b = 2]	.000	.000
[R = 1]* [x = 3]* [b = 1]	.000	.000
[R = 1]* [x = 3]* [b = 2]	.000	.000
[R = 2]* [x = 1]* [b = 1]	.000	.000
[R = 2]* [x = 1]* [b = 2]	.000	.000
[R = 2]* [x = 2]* [b = 1]	.000	.000
[R = 2]* [x = 2]* [b = 2]	.001	.000
[R = 2]* [x = 3]* [b = 1]	.002	.001
[R = 2]* [x = 3]* [b = 2]	.005	.002
[R = 3]* [x = 1]* [b = 1]	.015	.005
[R = 3]* [x = 1]* [b = 2]	.043	.015
[R = 3]* [x = 2]* [b = 1]	.122	.043
[R = 3]* [x = 2]* [b = 2]	.349	.122
[R = 3]* [x = 3]* [b = 1]	1.000	.349
[R = 3]* [x = 3]* [b = 2]	.349	1.000

Dependent Variable: y
Model: (Intercept), R, x, b, gender_g, income_g, age, R * x, R * x * b

a. The AR(1) working correlation matrix structure is computed assuming the measurements are equally spaced for all subjects.

Estimated Marginal Means 1: R

Estimates

R	Mean	Std. Error	95% Wald Confidence Interval<br>Lower	 Upper
kin	.61	.035	.54	.68
acquaintance	.35	.033	.29	.42
general other	.18	.023	.14	.23

Covariates appearing in the model are fixed at the following values: age=37.04

Pairwise Comparisons

(I) R	(J) R	Mean Difference (I-J)	Std. Error	df	Bonferroni Sig.
kin	acquaintance	.26[a]	.023	1	.000
	general other	.43[a]	.029	1	.000
acquaintance	kin	-.26[a]	.023	1	.000
	general other	.17[a]	.019	1	.000
general other	kin	-.43[a]	.029	1	.000
	acquaintance	-.17[a]	.019	1	.000

Pairwise Comparisons

		95% Wald Confidence Interval for Difference	
(I) R	(J) R	Lower	Upper
kin	acquaintance	.21	.32
	general other	.37	.50
acquaintance	kin	-.32	-.21
	general other	.13	.22
general other	kin	-.50	-.37
	acquaintance	-.22	-.13

Pairwise comparisons of estimated marginal means based on the original scale of dependent variable y

a. The mean difference is significant at the .05 level.

Overall Test Results

Wald Chi-Square	df	Sig.
228.196	2	.000

The Wald chi-square tests the effect of R. This test is based on the linearly independent pairwise comparisons among the estimated marginal means.

Estimated Marginal Means 2: x

Estimates

x	Mean	Std. Error	95% Wald Confidence Interval	
			Lower	Upper
2000	.67	.033	.60	.73
20,000	.37	.034	.31	.44
200,000	.13	.019	.10	.18

Covariates appearing in the model are fixed at the following values: age=37.04

Pairwise Comparisons

(I) x	(J) x	Mean Difference (I-J)	Std. Error	df	Bonferroni Sig.	95% Wald Confidence ...
						Lower
2000	20,000	.30[a]	.022	1	.000	.25
	200,000	.53[a]	.029	1	.000	.46
20,000	2000	-.30[a]	.022	1	.000	-.35
	200,000	.24[a]	.024	1	.000	.18
200,000	2000	-.53[a]	.029	1	.000	-.60
	20,000	-.24[a]	.024	1	.000	-.29

Pairwise Comparisons

(I) x	(J) x	95% Wald Confidence ...
		Upper
2000	20,000	.35
	200,000	.60
20,000	2000	-.25
	200,000	.29
200,000	2000	-.46
	20,000	-.18

Pairwise comparisons of estimated marginal means based on the original scale of dependent variable y

a. The mean difference is significant at the .05 level.

Overall Test Results

Wald Chi-Square	df	Sig.
356.083	2	.000

The Wald chi-square tests the effect of x. This test is based on the linearly independent pairwise comparisons among the estimated marginal means.

Estimated Marginal Means 3: b

Estimates

b	Mean	Std. Error	95% Wald Confidence Interval	
			Lower	Upper
H	.59	.035	.52	.65
L	.18	.023	.14	.23

Covariates appearing in the model are fixed at the following values: age=37.04

Pairwise Comparisons

(I) b	(J) b	Mean Difference (I-J)	Std. Error	df	Bonferroni Sig.	95% Wald Confidence ...
						Lower
H	L	.41[a]	.025	1	.000	.36
L	H	-.41[a]	.025	1	.000	-.45

Pairwise Comparisons

(I) b	(J) b	95% Wald Confidence ...
		Upper
H	L	.45
L	H	-.36

Pairwise comparisons of estimated marginal means based on the original scale of dependent variable y

a. The mean difference is significant at the .05 level.

Overall Test Results

Wald Chi-Square	df	Sig.
256.157	1	.000

The Wald chi-square tests the effect of b. This test is based on the linearly independent pairwise comparisons among the estimated marginal means.

Estimated Marginal Means 4: R* x

Estimates

R	x	Mean	Std. Error	95% Wald Confidence Interval	
				Lower	Upper
kin	2000	.83	.026	.78	.88
	20,000	.63	.037	.56	.70
	200,000	.31	.034	.25	.38
acquaintance	2000	.71	.034	.63	.77
	20,000	.36	.037	.29	.44
	200,000	.10	.018	.07	.14
general other	2000	.40	.037	.33	.48
	20,000	.17	.025	.13	.22
	200,000	.07	.014	.04	.10

Covariates appearing in the model are fixed at the following values: age=37.04

Pairwise Comparisons

x	(I) R	(J) R	Mean Difference (I-J)	Std. Error	df	Bonferroni Sig.
2000	kin	acquaintance	.13[a]	.021	1	.000
		general other	.43[a]	.031	1	.000
	acquaintance	kin	-.13[a]	.021	1	.000
		general other	.30[a]	.030	1	.000
	general other	kin	-.43[a]	.031	1	.000
		acquaintance	-.30[a]	.030	1	.000
20,000	kin	acquaintance	.27[a]	.029	1	.000
		general other	.47[a]	.032	1	.000
	acquaintance	kin	-.27[a]	.029	1	.000
		general other	.19[a]	.022	1	.000
	general other	kin	-.47[a]	.032	1	.000
		acquaintance	-.19[a]	.022	1	.000
200,000	kin	acquaintance	.21[a]	.028	1	.000
		general other	.24[a]	.030	1	.000
	acquaintance	kin	-.21[a]	.028	1	.000
		general other	.04[a]	.011	1	.004
	general other	kin	-.24[a]	.030	1	.000
		acquaintance	-.04[a]	.011	1	.004

Pairwise Comparisons

x	(I) R	(J) R	95% Wald Confidence Interval for Difference Lower	Upper
2000	kin	acquaintance	.08	.18
		general other	.36	.50
	acquaintance	kin	-.18	-.08
		general other	.23	.37
	general other	kin	-.50	-.36
		acquaintance	-.37	-.23
20,000	kin	acquaintance	.20	.34
		general other	.39	.54
	acquaintance	kin	-.34	-.20
		general other	.14	.25
	general other	kin	-.54	-.39
		acquaintance	-.25	-.14
200,000	kin	acquaintance	.14	.27
		general other	.17	.31
	acquaintance	kin	-.27	-.14
		general other	.01	.06
	general other	kin	-.31	-.17
		acquaintance	-.06	-.01

Pairwise comparisons of estimated marginal means based on the original scale of dependent variable y

a. The mean difference is significant at the .05 level.

Overall Test Results

x	Wald Chi-Square	df	Sig.
2000	192.182	2	.000
20,000	225.168	2	.000
200,000	63.760	2	.000

Each Wald chi-square tests the simple effects of R within each level combination of the other factors shown. These tests are based on the linearly independent pairwise comparisons among the estimated marginal means.

Estimated Marginal Means 5: R* x

Estimates

R	x	Mean	Std. Error	95% Wald Confidence Interval Lower	Upper
kin	2000	.83	.026	.78	.88
	20,000	.63	.037	.56	.70
	200,000	.31	.034	.25	.38
acquaintance	2000	.71	.034	.63	.77
	20,000	.36	.037	.29	.44
	200,000	.10	.018	.07	.14
general other	2000	.40	.037	.33	.48
	20,000	.17	.025	.13	.22
	200,000	.07	.014	.04	.10

Covariates appearing in the model are fixed at the following values: age=37.04

Pairwise Comparisons

R	(I) x	(J) x	Mean Difference (I-J)	Std. Error	df	Bonferroni Sig.
kin	2000	20,000	.20[a]	.024	1	.000
		200,000	.52[a]	.031	1	.000
	20,000	2000	-.20[a]	.024	1	.000
		200,000	.32[a]	.028	1	.000
	200,000	2000	-.52[a]	.031	1	.000
		20,000	-.32[a]	.028	1	.000
acquaintance	2000	20,000	.34[a]	.030	1	.000
		200,000	.60[a]	.033	1	.000
	20,000	2000	-.34[a]	.030	1	.000
		200,000	.26[a]	.029	1	.000
	200,000	2000	-.60[a]	.033	1	.000
		20,000	-.26[a]	.029	1	.000
general other	2000	20,000	.23[a]	.025	1	.000
		200,000	.34[a]	.033	1	.000
	20,000	2000	-.23[a]	.025	1	.000
		200,000	.10[a]	.018	1	.000
	200,000	2000	-.34[a]	.033	1	.000
		20,000	-.10[a]	.018	1	.000

Pairwise Comparisons

R	(I) x	(J) x	95% Wald Confidence Interval for Difference Lower	Upper
kin	2000	20,000	.14	.26
		200,000	.45	.60
	20,000	2000	-.26	-.14
		200,000	.26	.39
	200,000	2000	-.60	-.45
		20,000	-.39	-.26
acquaintance	2000	20,000	.27	.41
		200,000	.52	.68
	20,000	2000	-.41	-.27
		200,000	.19	.33
	200,000	2000	-.68	-.52
		20,000	-.33	-.19
general other	2000	20,000	.17	.29
		200,000	.26	.41
	20,000	2000	-.29	-.17
		200,000	.06	.14
	200,000	2000	-.41	-.26
		20,000	-.14	-.06

Pairwise comparisons of estimated marginal means based on the original scale of dependent variable y

a. The mean difference is significant at the .05 level.

Overall Test Results

R	Wald Chi-Square	df	Sig.
kin	284.948	2	.000
acquaintance	341.270	2	.000
general other	105.749	2	.000

Each Wald chi-square tests the simple effects of x within each level combination of the other factors shown. These tests are based on the linearly independent pairwise comparisons among the estimated marginal means.

Estimated Marginal Means 6: R* x* b

Estimates

R	x	b	Mean	Std. Error	95% Wald Confidence Interval	
					Lower	Upper
kin	2000	H	.93	.017	.89	.96
		L	.65	.037	.58	.72
	20,000	H	.86	.025	.80	.90
		L	.33	.036	.26	.40
	200,000	H	.53	.042	.45	.61
		L	.15	.025	.11	.21
acquaintance	2000	H	.89	.022	.84	.92
		L	.42	.040	.34	.50
	20,000	H	.71	.035	.64	.78
		L	.12	.022	.08	.16
	200,000	H	.18	.028	.13	.24
		L	.06	.014	.03	.09
general other	2000	H	.65	.038	.57	.72
		L	.20	.029	.15	.26
	20,000	H	.29	.035	.22	.36
		L	.09	.019	.06	.14
	200,000	H	.08	.017	.05	.12
		L	.06	.015	.04	.10

Covariates appearing in the model are fixed at the following values: age=37.04

Pairwise Comparisons

(I) R*x*b	(J) R*x*b	Mean Difference (I-J)	Std. Error	df	Bonferroni Sig.
[R=1]*[x=1]*[b=1]	[R=1]*[x=1]*[b=2]	.28[a]	.032	1	.000
	[R=1]*[x=2]*[b=1]	.07[a]	.017	1	.004
	[R=1]*[x=2]*[b=2]	.60[a]	.034	1	.000
	[R=1]*[x=3]*[b=1]	.40[a]	.037	1	.000
	[R=1]*[x=3]*[b=2]	.78[a]	.027	1	.000
	[R=2]*[x=1]*[b=1]	.04	.014	1	.499
	[R=2]*[x=1]*[b=2]	.51[a]	.037	1	.000
	[R=2]*[x=2]*[b=1]	.22[a]	.030	1	.000
	[R=2]*[x=2]*[b=2]	.81[a]	.025	1	.000
	[R=2]*[x=3]*[b=1]	.75[a]	.028	1	.000
	[R=2]*[x=3]*[b=2]	.87[a]	.021	1	.000
	[R=3]*[x=1]*[b=1]	.28[a]	.033	1	.000
	[R=3]*[x=1]*[b=2]	.73[a]	.029	1	.000
	[R=3]*[x=2]*[b=1]	.64[a]	.034	1	.000
	[R=3]*[x=2]*[b=2]	.84[a]	.023	1	.000
	[R=3]*[x=3]*[b=1]	.85[a]	.022	1	.000
	[R=3]*[x=3]*[b=2]	.87[a]	.021	1	.000
[R=1]*[x=1]*[b=2]	[R=1]*[x=1]*[b=1]	-.28[a]	.032	1	.000
	[R=1]*[x=2]*[b=1]	-.21[a]	.032	1	.000
	[R=1]*[x=2]*[b=2]	.32[a]	.030	1	.000
	[R=1]*[x=3]*[b=1]	.12	.040	1	.417
	[R=1]*[x=3]*[b=2]	.50[a]	.035	1	.000
	[R=2]*[x=1]*[b=1]	-.24[a]	.032	1	.000
	[R=2]*[x=1]*[b=2]	.23[a]	.034	1	.000
	[R=2]*[x=2]*[b=1]	-.06	.032	1	1.000
	[R=2]*[x=2]*[b=2]	.54[a]	.035	1	.000
	[R=2]*[x=3]*[b=1]	.47[a]	.037	1	.000
	[R=2]*[x=3]*[b=2]	.60[a]	.037	1	.000
	[R=3]*[x=1]*[b=1]	.00	.036	1	1.000
	[R=3]*[x=1]*[b=2]	.45[a]	.035	1	.000
	[R=3]*[x=2]*[b=1]	.36[a]	.037	1	.000
	[R=3]*[x=2]*[b=2]	.56[a]	.035	1	.000
	[R=3]*[x=3]*[b=1]	.58[a]	.038	1	.000
	[R=3]*[x=3]*[b=2]	.59[a]	.037	1	.000

Pairwise Comparisons

(I) R*x*b	(J) R*x*b	95% Wald Confidence Interval for Difference Lower	Upper
[R=1]*[x=1]*[b=1]	[R=1]*[x=1]*[b=2]	.16	.39
	[R=1]*[x=2]*[b=1]	.01	.13
	[R=1]*[x=2]*[b=2]	.48	.72
	[R=1]*[x=3]*[b=1]	.26	.53
	[R=1]*[x=3]*[b=2]	.68	.88
	[R=2]*[x=1]*[b=1]	-.01	.09
	[R=2]*[x=1]*[b=2]	.38	.65
	[R=2]*[x=2]*[b=1]	.11	.32
	[R=2]*[x=2]*[b=2]	.73	.90
	[R=2]*[x=3]*[b=1]	.65	.85
	[R=2]*[x=3]*[b=2]	.80	.95
	[R=3]*[x=1]*[b=1]	.16	.40
	[R=3]*[x=1]*[b=2]	.63	.84
	[R=3]*[x=2]*[b=1]	.52	.76
	[R=3]*[x=2]*[b=2]	.75	.92
	[R=3]*[x=3]*[b=1]	.77	.93
	[R=3]*[x=3]*[b=2]	.79	.95
[R=1]*[x=1]*[b=2]	[R=1]*[x=1]*[b=1]	-.39	-.16
	[R=1]*[x=2]*[b=1]	-.32	-.09
	[R=1]*[x=2]*[b=2]	.22	.43
	[R=1]*[x=3]*[b=1]	-.02	.26
	[R=1]*[x=3]*[b=2]	.38	.63
	[R=2]*[x=1]*[b=1]	-.35	-.12
	[R=2]*[x=1]*[b=2]	.11	.36
	[R=2]*[x=2]*[b=1]	-.18	.05
	[R=2]*[x=2]*[b=2]	.41	.66
	[R=2]*[x=3]*[b=1]	.34	.61
	[R=2]*[x=3]*[b=2]	.46	.73
	[R=3]*[x=1]*[b=1]	-.12	.13
	[R=3]*[x=1]*[b=2]	.33	.58
	[R=3]*[x=2]*[b=1]	.23	.50
	[R=3]*[x=2]*[b=2]	.43	.68
	[R=3]*[x=3]*[b=1]	.44	.71
	[R=3]*[x=3]*[b=2]	.46	.72

Pairwise Comparisons

(I) R*x*b	(J) R*x*b	Mean Difference (I-J)	Std. Error	df	Bonferroni Sig.
[R=1]*[x=2]*[b=1]	[R=1]*[x=1]*[b=1]	-.07[a]	.017	1	.004
	[R=1]*[x=1]*[b=2]	.21[a]	.032	1	.000
	[R=1]*[x=2]*[b=2]	.53[a]	.033	1	.000
	[R=1]*[x=3]*[b=1]	.33[a]	.034	1	.000
	[R=1]*[x=3]*[b=2]	.71[a]	.029	1	.000
	[R=2]*[x=1]*[b=1]	-.03	.017	1	1.000
	[R=2]*[x=1]*[b=2]	.44[a]	.037	1	.000
	[R=2]*[x=2]*[b=1]	.15[a]	.027	1	.000
	[R=2]*[x=2]*[b=2]	.74[a]	.028	1	.000
	[R=2]*[x=3]*[b=1]	.68[a]	.031	1	.000
	[R=2]*[x=3]*[b=2]	.80[a]	.027	1	.000
	[R=3]*[x=1]*[b=1]	.21[a]	.033	1	.000
	[R=3]*[x=1]*[b=2]	.66[a]	.033	1	.000
	[R=3]*[x=2]*[b=1]	.57[a]	.034	1	.000
	[R=3]*[x=2]*[b=2]	.77[a]	.027	1	.000
	[R=3]*[x=3]*[b=1]	.78[a]	.027	1	.000
	[R=3]*[x=3]*[b=2]	.80[a]	.027	1	.000
[R=1]*[x=2]*[b=2]	[R=1]*[x=1]*[b=1]	-.60[a]	.034	1	.000
	[R=1]*[x=1]*[b=2]	-.32[a]	.030	1	.000
	[R=1]*[x=2]*[b=1]	-.53[a]	.033	1	.000
	[R=1]*[x=3]*[b=1]	-.20[a]	.036	1	.000
	[R=1]*[x=3]*[b=2]	.18[a]	.028	1	.000
	[R=2]*[x=1]*[b=1]	-.56[a]	.034	1	.000
	[R=2]*[x=1]*[b=2]	-.09	.032	1	.875
	[R=2]*[x=2]*[b=1]	-.38[a]	.034	1	.000
	[R=2]*[x=2]*[b=2]	.21[a]	.030	1	.000
	[R=2]*[x=3]*[b=1]	.15[a]	.034	1	.002
	[R=2]*[x=3]*[b=2]	.27[a]	.035	1	.000
	[R=3]*[x=1]*[b=1]	-.32[a]	.041	1	.000
	[R=3]*[x=1]*[b=2]	.13[a]	.033	1	.010
	[R=3]*[x=2]*[b=1]	.04	.037	1	1.000
	[R=3]*[x=2]*[b=2]	.24[a]	.031	1	.000
	[R=3]*[x=3]*[b=1]	.25[a]	.034	1	.000
	[R=3]*[x=3]*[b=2]	.27[a]	.035	1	.000

Pairwise Comparisons

(I) R*x*b	(J) R*x*b	95% Wald Confidence Interval for Difference	
		Lower	Upper
[R=1]*[x=2]*[b=1]	[R=1]*[x=1]*[b=1]	-.13	-.01
	[R=1]*[x=1]*[b=2]	.09	.32
	[R=1]*[x=2]*[b=2]	.41	.65
	[R=1]*[x=3]*[b=1]	.20	.45
	[R=1]*[x=3]*[b=2]	.60	.82
	[R=2]*[x=1]*[b=1]	-.09	.03
	[R=2]*[x=1]*[b=2]	.31	.58
	[R=2]*[x=2]*[b=1]	.05	.24
	[R=2]*[x=2]*[b=2]	.64	.85
	[R=2]*[x=3]*[b=1]	.57	.79
	[R=2]*[x=3]*[b=2]	.71	.90
	[R=3]*[x=1]*[b=1]	.09	.33
	[R=3]*[x=1]*[b=2]	.54	.78
	[R=3]*[x=2]*[b=1]	.45	.69
	[R=3]*[x=2]*[b=2]	.67	.86
	[R=3]*[x=3]*[b=1]	.69	.88
	[R=3]*[x=3]*[b=2]	.70	.90
[R=1]*[x=2]*[b=2]	[R=1]*[x=1]*[b=1]	-.72	-.48
	[R=1]*[x=1]*[b=2]	-.43	-.22
	[R=1]*[x=2]*[b=1]	-.65	-.41
	[R=1]*[x=3]*[b=1]	-.33	-.07
	[R=1]*[x=3]*[b=2]	.08	.28
	[R=2]*[x=1]*[b=1]	-.68	-.44
	[R=2]*[x=1]*[b=2]	-.20	.03
	[R=2]*[x=2]*[b=1]	-.51	-.26
	[R=2]*[x=2]*[b=2]	.11	.32
	[R=2]*[x=3]*[b=1]	.03	.27
	[R=2]*[x=3]*[b=2]	.15	.40
	[R=3]*[x=1]*[b=1]	-.47	-.17
	[R=3]*[x=1]*[b=2]	.01	.25
	[R=3]*[x=2]*[b=1]	-.09	.17
	[R=3]*[x=2]*[b=2]	.12	.35
	[R=3]*[x=3]*[b=1]	.13	.38
	[R=3]*[x=3]*[b=2]	.14	.39

Pairwise Comparisons

(I) R*x*b	(J) R*x*b	Mean Difference (I-J)	Std. Error	df	Bonferroni Sig.
[R=1]*[x=3]*[b=1]	[R=1]*[x=1]*[b=1]	-.40[a]	.037	1	.000
	[R=1]*[x=1]*[b=2]	-.12	.040	1	.417
	[R=1]*[x=2]*[b=1]	-.33[a]	.034	1	.000
	[R=1]*[x=2]*[b=2]	.20[a]	.036	1	.000
	[R=1]*[x=3]*[b=2]	.38[a]	.036	1	.000
	[R=2]*[x=1]*[b=1]	-.36[a]	.037	1	.000
	[R=2]*[x=1]*[b=2]	.12	.041	1	.699
	[R=2]*[x=2]*[b=1]	-.18[a]	.035	1	.000
	[R=2]*[x=2]*[b=2]	.42[a]	.038	1	.000
	[R=2]*[x=3]*[b=1]	.35[a]	.035	1	.000
	[R=2]*[x=3]*[b=2]	.48[a]	.041	1	.000
	[R=3]*[x=1]*[b=1]	-.12	.041	1	.661
	[R=3]*[x=1]*[b=2]	.34[a]	.039	1	.000
	[R=3]*[x=2]*[b=1]	.25[a]	.038	1	.000
	[R=3]*[x=2]*[b=2]	.44[a]	.038	1	.000
	[R=3]*[x=3]*[b=1]	.46[a]	.040	1	.000
	[R=3]*[x=3]*[b=2]	.47[a]	.040	1	.000
[R=1]*[x=3]*[b=2]	[R=1]*[x=1]*[b=1]	-.78[a]	.027	1	.000
	[R=1]*[x=1]*[b=2]	-.50[a]	.035	1	.000
	[R=1]*[x=2]*[b=1]	-.71[a]	.029	1	.000
	[R=1]*[x=2]*[b=2]	-.18[a]	.028	1	.000
	[R=1]*[x=3]*[b=1]	-.38[a]	.036	1	.000
	[R=2]*[x=1]*[b=1]	-.74[a]	.029	1	.000
	[R=2]*[x=1]*[b=2]	-.27[a]	.035	1	.000
	[R=2]*[x=2]*[b=1]	-.56[a]	.034	1	.000
	[R=2]*[x=2]*[b=2]	.03	.023	1	1.000
	[R=2]*[x=3]*[b=1]	-.03	.026	1	1.000
	[R=2]*[x=3]*[b=2]	.09[a]	.023	1	.006
	[R=3]*[x=1]*[b=1]	-.50[a]	.039	1	.000
	[R=3]*[x=1]*[b=2]	-.05	.030	1	1.000
	[R=3]*[x=2]*[b=1]	-.14[a]	.035	1	.010
	[R=3]*[x=2]*[b=2]	.06	.024	1	1.000
	[R=3]*[x=3]*[b=1]	.07	.023	1	.281
	[R=3]*[x=3]*[b=2]	.09[a]	.024	1	.026

Pairwise Comparisons

(I) R*x*b	(J) R*x*b	95% Wald Confidence Interval for Difference Lower	Upper
[R=1]*[x=3]*[b=1]	[R=1]*[x=1]*[b=1]	-.53	-.26
	[R=1]*[x=1]*[b=2]	-.26	.02
	[R=1]*[x=2]*[b=1]	-.45	-.20
	[R=1]*[x=2]*[b=2]	.07	.33
	[R=1]*[x=3]*[b=2]	.25	.51
	[R=2]*[x=1]*[b=1]	-.49	-.22
	[R=2]*[x=1]*[b=2]	-.03	.26
	[R=2]*[x=2]*[b=1]	-.31	-.05
	[R=2]*[x=2]*[b=2]	.28	.55
	[R=2]*[x=3]*[b=1]	.23	.48
	[R=2]*[x=3]*[b=2]	.33	.62
	[R=3]*[x=1]*[b=1]	-.26	.03
	[R=3]*[x=1]*[b=2]	.19	.48
	[R=3]*[x=2]*[b=1]	.11	.38
	[R=3]*[x=2]*[b=2]	.30	.58
	[R=3]*[x=3]*[b=1]	.31	.60
	[R=3]*[x=3]*[b=2]	.33	.62
[R=1]*[x=3]*[b=2]	[R=1]*[x=1]*[b=1]	-.88	-.68
	[R=1]*[x=1]*[b=2]	-.63	-.38
	[R=1]*[x=2]*[b=1]	-.82	-.60
	[R=1]*[x=2]*[b=2]	-.28	-.08
	[R=1]*[x=3]*[b=1]	-.51	-.25
	[R=2]*[x=1]*[b=1]	-.84	-.64
	[R=2]*[x=1]*[b=2]	-.39	-.14
	[R=2]*[x=2]*[b=1]	-.69	-.44
	[R=2]*[x=2]*[b=2]	-.05	.12
	[R=2]*[x=3]*[b=1]	-.13	.06
	[R=2]*[x=3]*[b=2]	.01	.17
	[R=3]*[x=1]*[b=1]	-.64	-.36
	[R=3]*[x=1]*[b=2]	-.16	.06
	[R=3]*[x=2]*[b=1]	-.26	-.01
	[R=3]*[x=2]*[b=2]	-.03	.14
	[R=3]*[x=3]*[b=1]	-.01	.16
	[R=3]*[x=3]*[b=2]	.00	.17

Pairwise Comparisons

(I) R*x*b	(J) R*x*b	Mean Difference (I-J)	Std. Error	df	Bonferroni Sig.
[R=2]*[x=1]*[b=1]	[R=1]*[x=1]*[b=1]	-.04	.014	1	.499
	[R=1]*[x=1]*[b=2]	.24[a]	.032	1	.000
	[R=1]*[x=2]*[b=1]	.03	.017	1	1.000
	[R=1]*[x=2]*[b=2]	.56[a]	.034	1	.000
	[R=1]*[x=3]*[b=1]	.36[a]	.037	1	.000
	[R=1]*[x=3]*[b=2]	.74[a]	.029	1	.000
	[R=2]*[x=1]*[b=2]	.47[a]	.036	1	.000
	[R=2]*[x=2]*[b=1]	.18[a]	.027	1	.000
	[R=2]*[x=2]*[b=2]	.77[a]	.027	1	.000
	[R=2]*[x=3]*[b=1]	.71[a]	.029	1	.000
	[R=2]*[x=3]*[b=2]	.83[a]	.025	1	.000
	[R=3]*[x=1]*[b=1]	.24[a]	.031	1	.000
	[R=3]*[x=1]*[b=2]	.69[a]	.030	1	.000
	[R=3]*[x=2]*[b=1]	.60[a]	.033	1	.000
	[R=3]*[x=2]*[b=2]	.80[a]	.025	1	.000
	[R=3]*[x=3]*[b=1]	.81[a]	.025	1	.000
	[R=3]*[x=3]*[b=2]	.83[a]	.024	1	.000
[R=2]*[x=1]*[b=2]	[R=1]*[x=1]*[b=1]	-.51[a]	.037	1	.000
	[R=1]*[x=1]*[b=2]	-.23[a]	.034	1	.000
	[R=1]*[x=2]*[b=1]	-.44[a]	.037	1	.000
	[R=1]*[x=2]*[b=2]	.09	.032	1	.875
	[R=1]*[x=3]*[b=1]	-.12	.041	1	.699
	[R=1]*[x=3]*[b=2]	.27[a]	.035	1	.000
	[R=2]*[x=1]*[b=1]	-.47[a]	.036	1	.000
	[R=2]*[x=2]*[b=1]	-.30[a]	.034	1	.000
	[R=2]*[x=2]*[b=2]	.30[a]	.034	1	.000
	[R=2]*[x=3]*[b=1]	.24[a]	.036	1	.000
	[R=2]*[x=3]*[b=2]	.36[a]	.038	1	.000
	[R=3]*[x=1]*[b=1]	-.23[a]	.038	1	.000
	[R=3]*[x=1]*[b=2]	.22[a]	.033	1	.000
	[R=3]*[x=2]*[b=1]	.13	.039	1	.131
	[R=3]*[x=2]*[b=2]	.32[a]	.035	1	.000
	[R=3]*[x=3]*[b=1]	.34[a]	.038	1	.000
	[R=3]*[x=3]*[b=2]	.36[a]	.039	1	.000

Pairwise Comparisons

(I) R*x*b	(J) R*x*b	95% Wald Confidence Interval for Difference	
		Lower	Upper
[R=2]*[x=1]*[b=1]	[R=1]*[x=1]*[b=1]	-.09	.01
	[R=1]*[x=1]*[b=2]	.12	.35
	[R=1]*[x=2]*[b=1]	-.03	.09
	[R=1]*[x=2]*[b=2]	.44	.68
	[R=1]*[x=3]*[b=1]	.22	.49
	[R=1]*[x=3]*[b=2]	.64	.84
	[R=2]*[x=1]*[b=2]	.34	.60
	[R=2]*[x=2]*[b=1]	.08	.27
	[R=2]*[x=2]*[b=2]	.68	.87
	[R=2]*[x=3]*[b=1]	.60	.81
	[R=2]*[x=3]*[b=2]	.74	.92
	[R=3]*[x=1]*[b=1]	.13	.35
	[R=3]*[x=1]*[b=2]	.58	.80
	[R=3]*[x=2]*[b=1]	.48	.72
	[R=3]*[x=2]*[b=2]	.71	.89
	[R=3]*[x=3]*[b=1]	.72	.90
	[R=3]*[x=3]*[b=2]	.74	.92
[R=2]*[x=1]*[b=2]	[R=1]*[x=1]*[b=1]	-.65	-.38
	[R=1]*[x=1]*[b=2]	-.36	-.11
	[R=1]*[x=2]*[b=1]	-.58	-.31
	[R=1]*[x=2]*[b=2]	-.03	.20
	[R=1]*[x=3]*[b=1]	-.26	.03
	[R=1]*[x=3]*[b=2]	.14	.39
	[R=2]*[x=1]*[b=1]	-.60	-.34
	[R=2]*[x=2]*[b=1]	-.42	-.17
	[R=2]*[x=2]*[b=2]	.18	.42
	[R=2]*[x=3]*[b=1]	.11	.37
	[R=2]*[x=3]*[b=2]	.22	.50
	[R=3]*[x=1]*[b=1]	-.37	-.10
	[R=3]*[x=1]*[b=2]	.10	.34
	[R=3]*[x=2]*[b=1]	-.01	.27
	[R=3]*[x=2]*[b=2]	.20	.45
	[R=3]*[x=3]*[b=1]	.20	.48
	[R=3]*[x=3]*[b=2]	.22	.50

Pairwise Comparisons

(I) R*x*b	(J) R*x*b	Mean Difference (I-J)	Std. Error	df	Bonferroni Sig.
[R=2]*[x=2]*[b=1]	[R=1]*[x=1]*[b=1]	-.22[a]	.030	1	.000
	[R=1]*[x=1]*[b=2]	.06	.032	1	1.000
	[R=1]*[x=2]*[b=1]	-.15[a]	.027	1	.000
	[R=1]*[x=2]*[b=2]	.38[a]	.034	1	.000
	[R=1]*[x=3]*[b=1]	.18[a]	.035	1	.000
	[R=1]*[x=3]*[b=2]	.56[a]	.034	1	.000
	[R=2]*[x=1]*[b=1]	-.18[a]	.027	1	.000
	[R=2]*[x=1]*[b=2]	.30[a]	.034	1	.000
	[R=2]*[x=2]*[b=2]	.60[a]	.033	1	.000
	[R=2]*[x=3]*[b=1]	.53[a]	.032	1	.000
	[R=2]*[x=3]*[b=2]	.66[a]	.035	1	.000
	[R=3]*[x=1]*[b=1]	.06	.028	1	1.000
	[R=3]*[x=1]*[b=2]	.52[a]	.035	1	.000
	[R=3]*[x=2]*[b=1]	.43[a]	.033	1	.000
	[R=3]*[x=2]*[b=2]	.62[a]	.033	1	.000
	[R=3]*[x=3]*[b=1]	.64[a]	.034	1	.000
	[R=3]*[x=3]*[b=2]	.65[a]	.035	1	.000
[R=2]*[x=2]*[b=2]	[R=1]*[x=1]*[b=1]	-.81[a]	.025	1	.000
	[R=1]*[x=1]*[b=2]	-.54[a]	.035	1	.000
	[R=1]*[x=2]*[b=1]	-.74[a]	.028	1	.000
	[R=1]*[x=2]*[b=2]	-.21[a]	.030	1	.000
	[R=1]*[x=3]*[b=1]	-.42[a]	.038	1	.000
	[R=1]*[x=3]*[b=2]	-.03	.023	1	1.000
	[R=2]*[x=1]*[b=1]	-.77[a]	.027	1	.000
	[R=2]*[x=1]*[b=2]	-.30[a]	.034	1	.000
	[R=2]*[x=2]*[b=1]	-.60[a]	.033	1	.000
	[R=2]*[x=3]*[b=1]	-.07	.024	1	.960
	[R=2]*[x=3]*[b=2]	.06	.017	1	.085
	[R=3]*[x=1]*[b=1]	-.53[a]	.036	1	.000
	[R=3]*[x=1]*[b=2]	-.08	.023	1	.062
	[R=3]*[x=2]*[b=1]	-.17[a]	.029	1	.000
	[R=3]*[x=2]*[b=2]	.02	.011	1	1.000
	[R=3]*[x=3]*[b=1]	.04	.019	1	1.000
	[R=3]*[x=3]*[b=2]	.06	.018	1	.242

Pairwise Comparisons

(I) R*x*b	(J) R*x*b	95% Wald Confidence Interval for Difference	
		Lower	Upper
[R=2]*[x=2]*[b=1]	[R=1]*[x=1]*[b=1]	-.32	-.11
	[R=1]*[x=1]*[b=2]	-.05	.18
	[R=1]*[x=2]*[b=1]	-.24	-.05
	[R=1]*[x=2]*[b=2]	.26	.51
	[R=1]*[x=3]*[b=1]	.05	.31
	[R=1]*[x=3]*[b=2]	.44	.69
	[R=2]*[x=1]*[b=1]	-.27	-.08
	[R=2]*[x=1]*[b=2]	.17	.42
	[R=2]*[x=2]*[b=2]	.48	.72
	[R=2]*[x=3]*[b=1]	.42	.65
	[R=2]*[x=3]*[b=2]	.53	.78
	[R=3]*[x=1]*[b=1]	-.04	.17
	[R=3]*[x=1]*[b=2]	.39	.64
	[R=3]*[x=2]*[b=1]	.31	.54
	[R=3]*[x=2]*[b=2]	.50	.74
	[R=3]*[x=3]*[b=1]	.51	.76
	[R=3]*[x=3]*[b=2]	.53	.78
[R=2]*[x=2]*[b=2]	[R=1]*[x=1]*[b=1]	-.90	-.73
	[R=1]*[x=1]*[b=2]	-.66	-.41
	[R=1]*[x=2]*[b=1]	-.85	-.64
	[R=1]*[x=2]*[b=2]	-.32	-.11
	[R=1]*[x=3]*[b=1]	-.55	-.28
	[R=1]*[x=3]*[b=2]	-.12	.05
	[R=2]*[x=1]*[b=1]	-.87	-.68
	[R=2]*[x=1]*[b=2]	-.42	-.18
	[R=2]*[x=2]*[b=1]	-.72	-.48
	[R=2]*[x=3]*[b=1]	-.15	.02
	[R=2]*[x=3]*[b=2]	.00	.12
	[R=3]*[x=1]*[b=1]	-.66	-.40
	[R=3]*[x=1]*[b=2]	-.17	.00
	[R=3]*[x=2]*[b=1]	-.28	-.07
	[R=3]*[x=2]*[b=2]	-.02	.06
	[R=3]*[x=3]*[b=1]	-.03	.11
	[R=3]*[x=3]*[b=2]	-.01	.12

Pairwise Comparisons

(I) R*x*b	(J) R*x*b	Mean Difference (I-J)	Std. Error	df	Bonferroni Sig.
[R=2]*[x=3]*[b=1]	[R=1]*[x=1]*[b=1]	-.75[a]	.028	1	.000
	[R=1]*[x=1]*[b=2]	-.47[a]	.037	1	.000
	[R=1]*[x=2]*[b=1]	-.68[a]	.031	1	.000
	[R=1]*[x=2]*[b=2]	-.15[a]	.034	1	.002
	[R=1]*[x=3]*[b=1]	-.35[a]	.035	1	.000
	[R=1]*[x=3]*[b=2]	.03	.026	1	1.000
	[R=2]*[x=1]*[b=1]	-.71[a]	.029	1	.000
	[R=2]*[x=1]*[b=2]	-.24[a]	.036	1	.000
	[R=2]*[x=2]*[b=1]	-.53[a]	.032	1	.000
	[R=2]*[x=2]*[b=2]	.07	.024	1	.960
	[R=2]*[x=3]*[b=2]	.12[a]	.024	1	.000
	[R=3]*[x=1]*[b=1]	-.47[a]	.036	1	.000
	[R=3]*[x=1]*[b=2]	-.02	.029	1	1.000
	[R=3]*[x=2]*[b=1]	-.11	.030	1	.054
	[R=3]*[x=2]*[b=2]	.09	.025	1	.055
	[R=3]*[x=3]*[b=1]	.10[a]	.023	1	.001
	[R=3]*[x=3]*[b=2]	.12[a]	.025	1	.000
[R=2]*[x=3]*[b=2]	[R=1]*[x=1]*[b=1]	-.87[a]	.021	1	.000
	[R=1]*[x=1]*[b=2]	-.60[a]	.037	1	.000
	[R=1]*[x=2]*[b=1]	-.80[a]	.027	1	.000
	[R=1]*[x=2]*[b=2]	-.27[a]	.035	1	.000
	[R=1]*[x=3]*[b=1]	-.48[a]	.041	1	.000
	[R=1]*[x=3]*[b=2]	-.09[a]	.023	1	.006
	[R=2]*[x=1]*[b=1]	-.83[a]	.025	1	.000
	[R=2]*[x=1]*[b=2]	-.36[a]	.038	1	.000
	[R=2]*[x=2]*[b=1]	-.66[a]	.035	1	.000
	[R=2]*[x=2]*[b=2]	-.06	.017	1	.085
	[R=2]*[x=3]*[b=1]	-.12[a]	.024	1	.000
	[R=3]*[x=1]*[b=1]	-.59[a]	.038	1	.000
	[R=3]*[x=1]*[b=2]	-.14[a]	.027	1	.000
	[R=3]*[x=2]*[b=1]	-.23[a]	.032	1	.000
	[R=3]*[x=2]*[b=2]	-.04	.017	1	1.000
	[R=3]*[x=3]*[b=1]	-.02	.011	1	1.000
	[R=3]*[x=3]*[b=2]	.00	.012	1	1.000

Pairwise Comparisons

(I) R*x*b	(J) R*x*b	95% Wald Confidence Interval for Difference	
		Lower	Upper
[R=2]*[x=3]*[b=1]	[R=1]*[x=1]*[b=1]	-.85	-.65
	[R=1]*[x=1]*[b=2]	-.61	-.34
	[R=1]*[x=2]*[b=1]	-.79	-.57
	[R=1]*[x=2]*[b=2]	-.27	-.03
	[R=1]*[x=3]*[b=1]	-.48	-.23
	[R=1]*[x=3]*[b=2]	-.06	.13
	[R=2]*[x=1]*[b=1]	-.81	-.60
	[R=2]*[x=1]*[b=2]	-.37	-.11
	[R=2]*[x=2]*[b=1]	-.65	-.42
	[R=2]*[x=2]*[b=2]	-.02	.15
	[R=2]*[x=3]*[b=2]	.04	.21
	[R=3]*[x=1]*[b=1]	-.60	-.34
	[R=3]*[x=1]*[b=2]	-.12	.09
	[R=3]*[x=2]*[b=1]	-.21	.00
	[R=3]*[x=2]*[b=2]	.00	.18
	[R=3]*[x=3]*[b=1]	.02	.19
	[R=3]*[x=3]*[b=2]	.03	.21
[R=2]*[x=3]*[b=2]	[R=1]*[x=1]*[b=1]	-.95	-.80
	[R=1]*[x=1]*[b=2]	-.73	-.46
	[R=1]*[x=2]*[b=1]	-.90	-.71
	[R=1]*[x=2]*[b=2]	-.40	-.15
	[R=1]*[x=3]*[b=1]	-.62	-.33
	[R=1]*[x=3]*[b=2]	-.17	-.01
	[R=2]*[x=1]*[b=1]	-.92	-.74
	[R=2]*[x=1]*[b=2]	-.50	-.22
	[R=2]*[x=2]*[b=1]	-.78	-.53
	[R=2]*[x=2]*[b=2]	-.12	.00
	[R=2]*[x=3]*[b=1]	-.21	-.04
	[R=3]*[x=1]*[b=1]	-.73	-.46
	[R=3]*[x=1]*[b=2]	-.24	-.04
	[R=3]*[x=2]*[b=1]	-.35	-.12
	[R=3]*[x=2]*[b=2]	-.10	.02
	[R=3]*[x=3]*[b=1]	-.06	.02
	[R=3]*[x=3]*[b=2]	-.05	.04

Pairwise Comparisons

(I) R*x*b	(J) R*x*b	Mean Difference (I-J)	Std. Error	df	Bonferroni Sig.
[R=3]*[x=1]*[b=1]	[R=1]*[x=1]*[b=1]	-.28[a]	.033	1	.000
	[R=1]*[x=1]*[b=2]	.00	.036	1	1.000
	[R=1]*[x=2]*[b=1]	-.21[a]	.033	1	.000
	[R=1]*[x=2]*[b=2]	.32[a]	.041	1	.000
	[R=1]*[x=3]*[b=1]	.12	.041	1	.661
	[R=1]*[x=3]*[b=2]	.50[a]	.039	1	.000
	[R=2]*[x=1]*[b=1]	-.24[a]	.031	1	.000
	[R=2]*[x=1]*[b=2]	.23[a]	.038	1	.000
	[R=2]*[x=2]*[b=1]	-.06	.028	1	1.000
	[R=2]*[x=2]*[b=2]	.53[a]	.036	1	.000
	[R=2]*[x=3]*[b=1]	.47[a]	.036	1	.000
	[R=2]*[x=3]*[b=2]	.59[a]	.038	1	.000
	[R=3]*[x=1]*[b=2]	.45[a]	.033	1	.000
	[R=3]*[x=2]*[b=1]	.36[a]	.031	1	.000
	[R=3]*[x=2]*[b=2]	.56[a]	.036	1	.000
	[R=3]*[x=3]*[b=1]	.57[a]	.037	1	.000
	[R=3]*[x=3]*[b=2]	.59[a]	.037	1	.000
[R=3]*[x=1]*[b=2]	[R=1]*[x=1]*[b=1]	-.73[a]	.029	1	.000
	[R=1]*[x=1]*[b=2]	-.45[a]	.035	1	.000
	[R=1]*[x=2]*[b=1]	-.66[a]	.033	1	.000
	[R=1]*[x=2]*[b=2]	-.13[a]	.033	1	.010
	[R=1]*[x=3]*[b=1]	-.34[a]	.039	1	.000
	[R=1]*[x=3]*[b=2]	.05	.030	1	1.000
	[R=2]*[x=1]*[b=1]	-.69[a]	.030	1	.000
	[R=2]*[x=1]*[b=2]	-.22[a]	.033	1	.000
	[R=2]*[x=2]*[b=1]	-.52[a]	.035	1	.000
	[R=2]*[x=2]*[b=2]	.08	.023	1	.062
	[R=2]*[x=3]*[b=1]	.02	.029	1	1.000
	[R=2]*[x=3]*[b=2]	.14[a]	.027	1	.000
	[R=3]*[x=1]*[b=1]	-.45[a]	.033	1	.000
	[R=3]*[x=2]*[b=1]	-.09	.030	1	.388
	[R=3]*[x=2]*[b=2]	.10[a]	.021	1	.000
	[R=3]*[x=3]*[b=1]	.12[a]	.027	1	.001
	[R=3]*[x=3]*[b=2]	.14[a]	.026	1	.000

Pairwise Comparisons

(I) R*x*b	(J) R*x*b	95% Wald Confidence Interval for Difference	
		Lower	Upper
[R=3]*[x=1]*[b=1]	[R=1]*[x=1]*[b=1]	-.40	-.16
	[R=1]*[x=1]*[b=2]	-.13	.12
	[R=1]*[x=2]*[b=1]	-.33	-.09
	[R=1]*[x=2]*[b=2]	.17	.47
	[R=1]*[x=3]*[b=1]	-.03	.26
	[R=1]*[x=3]*[b=2]	.36	.64
	[R=2]*[x=1]*[b=1]	-.35	-.13
	[R=2]*[x=1]*[b=2]	.10	.37
	[R=2]*[x=2]*[b=1]	-.17	.04
	[R=2]*[x=2]*[b=2]	.40	.66
	[R=2]*[x=3]*[b=1]	.34	.60
	[R=2]*[x=3]*[b=2]	.46	.73
	[R=3]*[x=1]*[b=2]	.33	.57
	[R=3]*[x=2]*[b=1]	.25	.47
	[R=3]*[x=2]*[b=2]	.43	.68
	[R=3]*[x=3]*[b=1]	.44	.70
	[R=3]*[x=3]*[b=2]	.45	.72
[R=3]*[x=1]*[b=2]	[R=1]*[x=1]*[b=1]	-.84	-.63
	[R=1]*[x=1]*[b=2]	-.58	-.33
	[R=1]*[x=2]*[b=1]	-.78	-.54
	[R=1]*[x=2]*[b=2]	-.25	-.01
	[R=1]*[x=3]*[b=1]	-.48	-.19
	[R=1]*[x=3]*[b=2]	-.06	.16
	[R=2]*[x=1]*[b=1]	-.80	-.58
	[R=2]*[x=1]*[b=2]	-.34	-.10
	[R=2]*[x=2]*[b=1]	-.64	-.39
	[R=2]*[x=2]*[b=2]	.00	.17
	[R=2]*[x=3]*[b=1]	-.09	.12
	[R=2]*[x=3]*[b=2]	.04	.24
	[R=3]*[x=1]*[b=1]	-.57	-.33
	[R=3]*[x=2]*[b=1]	-.20	.02
	[R=3]*[x=2]*[b=2]	.03	.18
	[R=3]*[x=3]*[b=1]	.02	.22
	[R=3]*[x=3]*[b=2]	.05	.23

Pairwise Comparisons

(I) R*x*b	(J) R*x*b	Mean Difference (I-J)	Std. Error	df	Bonferroni Sig.
[R=3]*[x=2]*[b=1]	[R=1]*[x=1]*[b=1]	-.64[a]	.034	1	.000
	[R=1]*[x=1]*[b=2]	-.36[a]	.037	1	.000
	[R=1]*[x=2]*[b=1]	-.57[a]	.034	1	.000
	[R=1]*[x=2]*[b=2]	-.04	.037	1	1.000
	[R=1]*[x=3]*[b=1]	-.25[a]	.038	1	.000
	[R=1]*[x=3]*[b=2]	.14[a]	.035	1	.010
	[R=2]*[x=1]*[b=1]	-.60[a]	.033	1	.000
	[R=2]*[x=1]*[b=2]	-.13	.039	1	.131
	[R=2]*[x=2]*[b=1]	-.43[a]	.033	1	.000
	[R=2]*[x=2]*[b=2]	.17[a]	.029	1	.000
	[R=2]*[x=3]*[b=1]	.11	.030	1	.054
	[R=2]*[x=3]*[b=2]	.23[a]	.032	1	.000
	[R=3]*[x=1]*[b=1]	-.36[a]	.031	1	.000
	[R=3]*[x=1]*[b=2]	.09	.030	1	.388
	[R=3]*[x=2]*[b=2]	.19[a]	.029	1	.000
	[R=3]*[x=3]*[b=1]	.21[a]	.031	1	.000
	[R=3]*[x=3]*[b=2]	.23[a]	.033	1	.000
[R=3]*[x=2]*[b=2]	[R=1]*[x=1]*[b=1]	-.84[a]	.023	1	.000
	[R=1]*[x=1]*[b=2]	-.56[a]	.035	1	.000
	[R=1]*[x=2]*[b=1]	-.77[a]	.027	1	.000
	[R=1]*[x=2]*[b=2]	-.24[a]	.031	1	.000
	[R=1]*[x=3]*[b=1]	-.44[a]	.038	1	.000
	[R=1]*[x=3]*[b=2]	-.06	.024	1	1.000
	[R=2]*[x=1]*[b=1]	-.80[a]	.025	1	.000
	[R=2]*[x=1]*[b=2]	-.32[a]	.035	1	.000
	[R=2]*[x=2]*[b=1]	-.62[a]	.033	1	.000
	[R=2]*[x=2]*[b=2]	-.02	.011	1	1.000
	[R=2]*[x=3]*[b=1]	-.09	.025	1	.055
	[R=2]*[x=3]*[b=2]	.04	.017	1	1.000
	[R=3]*[x=1]*[b=1]	-.56[a]	.036	1	.000
	[R=3]*[x=1]*[b=2]	-.10[a]	.021	1	.000
	[R=3]*[x=2]*[b=1]	-.19[a]	.029	1	.000
	[R=3]*[x=3]*[b=1]	.02	.017	1	1.000
	[R=3]*[x=3]*[b=2]	.03	.013	1	1.000

Pairwise Comparisons

(I) R*x*b	(J) R*x*b	95% Wald Confidence Interval for Difference	
		Lower	Upper
[R=3]*[x=2]*[b=1]	[R=1]*[x=1]*[b=1]	-.76	-.52
	[R=1]*[x=1]*[b=2]	-.50	-.23
	[R=1]*[x=2]*[b=1]	-.69	-.45
	[R=1]*[x=2]*[b=2]	-.17	.09
	[R=1]*[x=3]*[b=1]	-.38	-.11
	[R=1]*[x=3]*[b=2]	.01	.26
	[R=2]*[x=1]*[b=1]	-.72	-.48
	[R=2]*[x=1]*[b=2]	-.27	.01
	[R=2]*[x=2]*[b=1]	-.54	-.31
	[R=2]*[x=2]*[b=2]	.07	.28
	[R=2]*[x=3]*[b=1]	.00	.21
	[R=2]*[x=3]*[b=2]	.12	.35
	[R=3]*[x=1]*[b=1]	-.47	-.25
	[R=3]*[x=1]*[b=2]	-.02	.20
	[R=3]*[x=2]*[b=2]	.09	.30
	[R=3]*[x=3]*[b=1]	.10	.32
	[R=3]*[x=3]*[b=2]	.11	.34
[R=3]*[x=2]*[b=2]	[R=1]*[x=1]*[b=1]	-.92	-.75
	[R=1]*[x=1]*[b=2]	-.68	-.43
	[R=1]*[x=2]*[b=1]	-.86	-.67
	[R=1]*[x=2]*[b=2]	-.35	-.12
	[R=1]*[x=3]*[b=1]	-.58	-.30
	[R=1]*[x=3]*[b=2]	-.14	.03
	[R=2]*[x=1]*[b=1]	-.89	-.71
	[R=2]*[x=1]*[b=2]	-.45	-.20
	[R=2]*[x=2]*[b=1]	-.74	-.50
	[R=2]*[x=2]*[b=2]	-.06	.02
	[R=2]*[x=3]*[b=1]	-.18	.00
	[R=2]*[x=3]*[b=2]	-.02	.10
	[R=3]*[x=1]*[b=1]	-.68	-.43
	[R=3]*[x=1]*[b=2]	-.18	-.03
	[R=3]*[x=2]*[b=1]	-.30	-.09
	[R=3]*[x=3]*[b=1]	-.04	.08
	[R=3]*[x=3]*[b=2]	-.01	.08

Pairwise Comparisons

(I) R*x*b	(J) R*x*b	Mean Difference (I-J)	Std. Error	df	Bonferroni Sig.
[R=3]*[x=3]*[b=1]	[R=1]*[x=1]*[b=1]	-.85[a]	.022	1	.000
	[R=1]*[x=1]*[b=2]	-.58[a]	.038	1	.000
	[R=1]*[x=2]*[b=1]	-.78[a]	.027	1	.000
	[R=1]*[x=2]*[b=2]	-.25[a]	.034	1	.000
	[R=1]*[x=3]*[b=1]	-.46[a]	.040	1	.000
	[R=1]*[x=3]*[b=2]	-.07	.023	1	.281
	[R=2]*[x=1]*[b=1]	-.81[a]	.025	1	.000
	[R=2]*[x=1]*[b=2]	-.34[a]	.038	1	.000
	[R=2]*[x=2]*[b=1]	-.64[a]	.034	1	.000
	[R=2]*[x=2]*[b=2]	-.04	.019	1	1.000
	[R=2]*[x=3]*[b=1]	-.10[a]	.023	1	.001
	[R=2]*[x=3]*[b=2]	.02	.011	1	1.000
	[R=3]*[x=1]*[b=1]	-.57[a]	.037	1	.000
	[R=3]*[x=1]*[b=2]	-.12[a]	.027	1	.001
	[R=3]*[x=2]*[b=1]	-.21[a]	.031	1	.000
	[R=3]*[x=2]*[b=2]	-.02	.017	1	1.000
	[R=3]*[x=3]*[b=2]	.02	.014	1	1.000
[R=3]*[x=3]*[b=2]	[R=1]*[x=1]*[b=1]	-.87[a]	.021	1	.000
	[R=1]*[x=1]*[b=2]	-.59[a]	.037	1	.000
	[R=1]*[x=2]*[b=1]	-.80[a]	.027	1	.000
	[R=1]*[x=2]*[b=2]	-.27[a]	.035	1	.000
	[R=1]*[x=3]*[b=1]	-.47[a]	.040	1	.000
	[R=1]*[x=3]*[b=2]	-.09[a]	.024	1	.026
	[R=2]*[x=1]*[b=1]	-.83[a]	.024	1	.000
	[R=2]*[x=1]*[b=2]	-.36[a]	.039	1	.000
	[R=2]*[x=2]*[b=1]	-.65[a]	.035	1	.000
	[R=2]*[x=2]*[b=2]	-.06	.018	1	.242
	[R=2]*[x=3]*[b=1]	-.12[a]	.025	1	.000
	[R=2]*[x=3]*[b=2]	.00	.012	1	1.000
	[R=3]*[x=1]*[b=1]	-.59[a]	.037	1	.000
	[R=3]*[x=1]*[b=2]	-.14[a]	.026	1	.000
	[R=3]*[x=2]*[b=1]	-.23[a]	.033	1	.000
	[R=3]*[x=2]*[b=2]	-.03	.013	1	1.000
	[R=3]*[x=3]*[b=1]	-.02	.014	1	1.000

Pairwise Comparisons

(I) R*x*b	(J) R*x*b	95% Wald Confidence Interval for Difference	
		Lower	Upper
[R=3]*[x=3]*[b=1]	[R=1]*[x=1]*[b=1]	-.93	-.77
	[R=1]*[x=1]*[b=2]	-.71	-.44
	[R=1]*[x=2]*[b=1]	-.88	-.69
	[R=1]*[x=2]*[b=2]	-.38	-.13
	[R=1]*[x=3]*[b=1]	-.60	-.31
	[R=1]*[x=3]*[b=2]	-.16	.01
	[R=2]*[x=1]*[b=1]	-.90	-.72
	[R=2]*[x=1]*[b=2]	-.48	-.20
	[R=2]*[x=2]*[b=1]	-.76	-.51
	[R=2]*[x=2]*[b=2]	-.11	.03
	[R=2]*[x=3]*[b=1]	-.19	-.02
	[R=2]*[x=3]*[b=2]	-.02	.06
	[R=3]*[x=1]*[b=1]	-.70	-.44
	[R=3]*[x=1]*[b=2]	-.22	-.02
	[R=3]*[x=2]*[b=1]	-.32	-.10
	[R=3]*[x=2]*[b=2]	-.08	.04
	[R=3]*[x=3]*[b=2]	-.03	.07
[R=3]*[x=3]*[b=2]	[R=1]*[x=1]*[b=1]	-.95	-.79
	[R=1]*[x=1]*[b=2]	-.72	-.46
	[R=1]*[x=2]*[b=1]	-.90	-.70
	[R=1]*[x=2]*[b=2]	-.39	-.14
	[R=1]*[x=3]*[b=1]	-.62	-.33
	[R=1]*[x=3]*[b=2]	-.17	.00
	[R=2]*[x=1]*[b=1]	-.92	-.74
	[R=2]*[x=1]*[b=2]	-.50	-.22
	[R=2]*[x=2]*[b=1]	-.78	-.53
	[R=2]*[x=2]*[b=2]	-.12	.01
	[R=2]*[x=3]*[b=1]	-.21	-.03
	[R=2]*[x=3]*[b=2]	-.04	.05
	[R=3]*[x=1]*[b=1]	-.72	-.45
	[R=3]*[x=1]*[b=2]	-.23	-.05
	[R=3]*[x=2]*[b=1]	-.34	-.11
	[R=3]*[x=2]*[b=2]	-.08	.01
	[R=3]*[x=3]*[b=1]	-.07	.03

Pairwise comparisons of estimated marginal means based on the original scale of dependent variable y

a. The mean difference is significant at the .05 level.

Overall Test Results

Wald Chi-Square	df	Sig.
1900.488	17	.000

The Wald chi-square tests the effect of R*x*b. This test is based on the linearly independent pairwise comparisons among the estimated marginal means.
